# Control of COVID‐19 Outbreaks under Stochastic Community Dynamics, Bimodality, or Limited Vaccination

**DOI:** 10.1002/advs.202200088

**Published:** 2022-05-23

**Authors:** Björn Goldenbogen, Stephan O. Adler, Oliver Bodeit, Judith A. H. Wodke, Ximena Escalera‐Fanjul, Aviv Korman, Maria Krantz, Lasse Bonn, Rafael Morán‐Torres, Johanna E. L. Haffner, Maxim Karnetzki, Ivo Maintz, Lisa Mallis, Hannah Prawitz, Patrick S. Segelitz, Martin Seeger, Rune Linding, Edda Klipp

**Affiliations:** ^1^ Theoretical Biophysics Humboldt‐Universität zu Berlin Invalidenstr. 42 Berlin 10115 Germany; ^2^ Institute of Biochemistry Charité – Universitätsmedizin Berlin Virchowweg 6 Berlin 10117 Germany; ^3^ Institute of Quantitative and Theoretical Biology Heinrich‐Heine‐Universität Universitätsstraße 1 Düsseldorf 40225 Germany; ^4^ Rewire Tx Humboldt‐Universität zu Berlin Invalidenstr. 42 Berlin 10115 Germany

**Keywords:** bimodality, COVID‐19, epidemiology, respiratory diseases, stochastic agent‐based modeling

## Abstract

Reaching population immunity against COVID‐19 is proving difficult even in countries with high vaccination levels. Thus, it is critical to identify limits of control and effective measures against future outbreaks. The effects of nonpharmaceutical interventions (NPIs) and vaccination strategies are analyzed with a detailed community‐specific agent‐based model (ABM). The authors demonstrate that the threshold for population immunity is not a unique number, but depends on the vaccination strategy. Prioritizing highly interactive people diminishes the risk for an infection wave, while prioritizing the elderly minimizes fatalities when vaccinations are low. Control over COVID‐19 outbreaks requires adaptive combination of NPIs and targeted vaccination, exemplified for Germany for January–September 2021. Bimodality emerges from the heterogeneity and stochasticity of community‐specific human–human interactions and infection networks, which can render the effects of limited NPIs uncertain. The authors' simulation platform can process and analyze dynamic COVID‐19 epidemiological situations in diverse communities worldwide to predict pathways to population immunity even with limited vaccination.

## Graphical abstract

The limits of control and the effect of measures against future COVID‐19 outbreaks are identified using a detailed community‐specific ABM. The demographic and geographic structure of assessed communities influence the pattern of infection spreading and the resulting *R*‐values. Stochastic community dynamics and limited vaccination can lead to bimodal outcomes, rendering predictions about infection spreading and effects of nonpharmaceutical interventions uncertain.

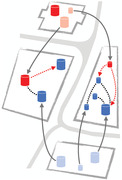

## 1 Introduction

In response to the COVID‐19 pandemic, major efforts have been carried out to provide models and data‐driven support for public health and government decision making. The scientific community has made great efforts to analyze the available data,^[^ ^1^, ^2^, ^3^, ^4^, ^5^, ^6^ ^]^ to predict virus propagation and the influence of different societal aspects such as age‐dependent contact levels,^[^ ^7^, ^8^, ^9^ ^]^ and to provide scientific evidence for the efficacy of different mitigation measures, testing or vaccination strategies.^[^ ^10^, ^11^, ^12^ ^]^ The GLEaM framework integrates worldwide high‐resolution demographic and mobility data to simulate disease spread on the global scale.^[^ ^13^ ^]^

Some studies focused on individual countries,^[^ ^8^, ^14^, ^15^, ^16^, ^17^, ^18^, ^19^, ^20^, ^21^ ^]^ others aimed to integrate worldwide high‐resolution demographic and mobility data to simulate the disease.^[^ ^22^, ^23^ ^]^ Still, in a comment, Bouffanais and Lim pinpointed the discrepancy between the detail levels of available data and mathematical models versus the requested information to effectively prevent uncontrollable virus spreading.^[^ ^24^ ^]^ There is often a discrepancy between what is required for specific, effective, and fast decision making and what models can actually offer.^[^ ^8^, ^21^ ^]^ One potential reason is that many models are based on population‐wide assertions and not on human individuals.^[^ ^25^, ^26^ ^]^

Accordingly, stochastic ABMs became a useful tool for epidemiologic simulations,^[^ ^18^, ^27^, ^28^, ^29^, ^30^, ^31^, ^32^ ^]^ being particularly relevant at initial stages of outbreaks when incidence numbers are low.^[^ ^33^ ^]^ Currently, with an increasing proportion of immunized in the population and new virus variants emerging, it is vital to capture stochasticity and structural heterogeneity within the system. Many recently published ABMs on COVID‐19 focused on individual countries,^[^ ^14^, ^16^, ^17^, ^18^, ^34^ ^]^ some showed the relevance of age‐dependent social mixing,^[^ ^4^, ^5^, ^9^, ^20^, ^35^, ^36^ ^]^ location and travels,^[^ ^16^, ^19^ ^]^ or the effect of presymptomatic and nondiagnosed transmissions^[^ ^8^, ^21^, ^37^ ^]^ on viral spreading. With respect to data usage some of the models reflect the demographics of the population,^[^ ^18^, ^30^ ^]^ others integrated detailed geographic data, either for locations or for mobility information.^[^ ^16^, ^19^, ^28^ ^]^ However, a combination of detailed geographic and demographic data is necessary when striving for simulating contacts in realistic communities. Further, to predict virus propagation on a more specific, not necessarily global level, we require heterogeneous models taking into account not only age cohorts and social activity levels but also other types of heterogeneities such as households characteristics or different locations types like schools or workplaces.^[^ ^8^, ^37^ ^]^

Spreading of SARS‐CoV‐2 in the population is a complex system and must be analyzed accordingly. Viral outbreaks are nonlinear, stochastic, network‐based, and localized. They depend on human behavior as well as on contact patterns^[^ ^8^, ^37^ ^]^ between groups of individuals.^[^ ^35^, ^36^ ^]^ We explore whether essential aspects of the system's behavior (e.g., stochasticity and bimodality) may be missed if human behavior is not sufficiently considered or if the system is assumed to be homogeneous or modeled deterministically.

Human behavior is perhaps most important to consider in the pursuit of population immunity for COVID‐19 as defined by WHO: “'Herd immunity', also known as 'population immunity', is the indirect protection from an infectious disease that happens when a population is immune either through vaccination or immunity developed through previous infection.” (who.int/news‐room/q‐a‐detail/herd‐immunity‐lockdowns‐and‐COVID‐19). Even in countries like Israel with relatively high vaccine uptake^[^ ^38^ ^]^ reaching population immunity appears elusive;^[^ ^39^ ^]^ or the Seychelles, a nation among the fastest to vaccinate its population (>70% received at least one vaccine dose by May 12th 2021), had subsequently experienced a surge in infection cases.^[^ ^6^ ^]^ These challenges are further emboldened by the relatively high attack‐rate demonstrated for SARS‐CoV‐2,^[^ ^40^ ^]^ and the fact that many countries are still faced with limited vaccine supply or vaccine hesitancy.

Thus, it is critical to further explore the potential for epidemiological governance based on community‐specific spatio‐temporal adaptive combinations of NPIs and targeted vaccination.^[^ ^41^ ^]^ To keep vulnerable citizens, heterogeneous, and diverse societies safe, for example, from new variants, while allowing economic and social activity to resume, NPIs and vaccination strategies have to be continuously adapted and combined to reach and maintain population immunity and minimize fatalities.

We present a platform to analyze virus spreading as well as the effects of NPIs and adaptive population immunity strategies in different communities, considering their specific demographic and geographic characteristics. Based on precision simulation of individualized, real‐world, spatio‐temporal SARS‐CoV‐2 transmission networks the methodology can determine adaptive combinations and optimality in intervention strategies. By predicting the effects of combining vaccination strategies with NPIs in an adaptive, context‐specific manner, we demonstrate how to effectively strive for population immunity and control outbreaks. The emergence of bimodality is critical for correct interpretation of real‐world results from NPIs and in forward‐looking decision processes. The platform is open source and readily applicable to diverse communities worldwide, and offers the ability to identify key aspects of COVID‐19 outbreaks that neither epidemiological homogenous mixing nor deterministic models can detect.

## 2 Results

### 2.1 Heterogeneous Model of Human–Human Interactions Enables Precision‐Simulation of COVID‐19 Outbreaks

**Figure 1:**
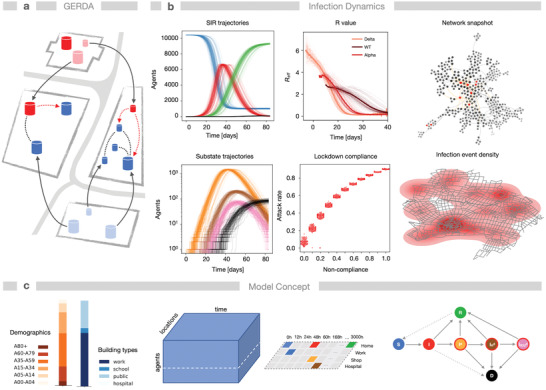
The network effects of SARS‐CoV‐2 outbreaks are quantified with heterogeneous spatio‐temporal models of individual human behavior. Human–human interactions create dynamic stochastic networks in space and time. a) The model (GERDA) describes potentially infectious interactions of individuals (red/blue shapes) of a simulated population inside real‐world buildings (gray squares) and the resulting disease progression. b) The model monitors trajectories of infection states: susceptible S (blue), infected I (red), recovered R (green), dead D (black), and substates diagnosed I^d^ (orange), hospitalized I^d^_H_, (brown), or admitted to ICU I^d^_ICU_ (as in [c]). Results stem from repeated simulations of the baseline scenario (Gangelt, Germany with 10.351 individuals, *N* = 96 simulations, no measures), starting from four infected individuals. The emerging dynamics of the effective reproduction number *R*_eff_ is shown for different virus strains. The performance of NPIs is exemplarily shown for noncompliance (as ratio of population *F*_c_) with a lockdown leading to higher attack rates. Right column: snapshot of the interaction and infection network after 15 h. Below: geographical infection density of a real‐world community, shown as kernel density of simulated infection events in Gangelt in 1 week, see also (Movie S1, Supporting Information). Parameter values: infectivity *k*_I_ = 0.14, interaction frequency *μ* = 2. c) The model incorporates demographic, geographic data, and individual daily routines of the members of the simulated population, thereby creating temporal contact networks (b). Bottom right: state transitions for disease progression.

Encounters between individuals in time and space form human–human interaction networks (HHIN) which depend on human behavior (**Figure** **1**). Spreading of respiratory diseases, such as COVID‐19, creates subnetworks of infection HHIN (iHHIN). These networks are stochastic and evolve over time. Their analysis requires models that can capture this complexity of human–human interactions. We built a detailed ABM,^[^ ^42^ ^]^ in which HHIN realizations are results of simulations instead of an input. Every agent represents a human individual within a real‐world community (Figure 1 and Experimental Section and Supporting Information and Figures S1–S69 and Tables S1–S27, Supporting Information). The community is characterized by its geographic information,^[^ ^43^, ^44^ ^]^ demographic data,^[^ ^43^, ^44^ ^]^ and realistic occupations and weekly schedules of the agents (Figure S6, Supporting Information). Agents can meet at different places in the community and their interactions create time‐dependent HHINs. We complemented the approach with a classical SIR model for the states of individual agents extended by clinically described stages of SARS‐CoV‐2 infection^[^ ^45^ ^]^ and COVID‐19 (Figure 1c and Experimental Section). While interactions within locations at a time point result in random graphs, reflecting airborne transmission, HHINs emerging over time are heterogeneous. Infection transmission among agents during the simulation yields the respective iHHIN (Figure 1b). This enables us to simulate the dynamics of the pandemic for realistic scenarios and realistic populations. The model was parameterized for communities in Germany, Israel, and Mexico. The German town Gangelt, which witnessed one of the first COVID‐19 outbreaks,^[^ ^46^ ^]^ serves as a running example.

We compared simulations of the baseline scenario, that is, the infection spread from a few individuals through the population without any NPIs, with simulations of various NPIs: full lockdown, closure and reopening of selected locations, noncompliance with interventions as well as the effect of different values of infectivity (where lowering mimics social distancing or mask wearing, while new strains such as Alpha or Delta lead to an increase). Classical parameters of epidemiological models such as attack rate (AR, ratio of total number of infected to total number of susceptible individuals at the start) and *R*‐value (the average of the number of secondary cases) were derived from simulations to compare the resulting insights into the pandemics. To represent the ancestral strain (wild type, WT) and the virus variants of concern (VOC), B.1.1.7 (Alpha) and B 1.617.2 (Delta), we varied the infectivity (*k*_I_‐values of 0.14, 0.24 and 0.34) and obtained average *R*_0_‐values (the average of the number of secondary cases, assuming the population is completely susceptible, calculated from the first 1% infected per simulation) of 2.83, 4.28, and 5.58, respectively, which compare to reported values.^[^ ^47^, ^48^ ^]^ We analyzed the effect of different vaccination strategies as well as the combination of NPIs and vaccination on infection and disease dynamics, considering also the prevalence of different variants of SARS‐CoV‐2.

### 2.2 Community Specifics Alter Dynamics and Patterns of SARS‐CoV‐2 Outbreaks

We set out to determine how infection networks vary as the result of community‐specific interaction networks and how they can be quantified to enable the differentiation of characteristic patterns of community‐based viral spreading routes. The HHINs encompass three different classes of interactions, namely those that: 1) cannot lead to infection transmission (e.g., between two S), 2) can potentially result in transmission (interaction between S and I), and 3) result in infection transmission from I to S defining the iHHIN (Figure S37, Supporting Information).

**Figure 2:**
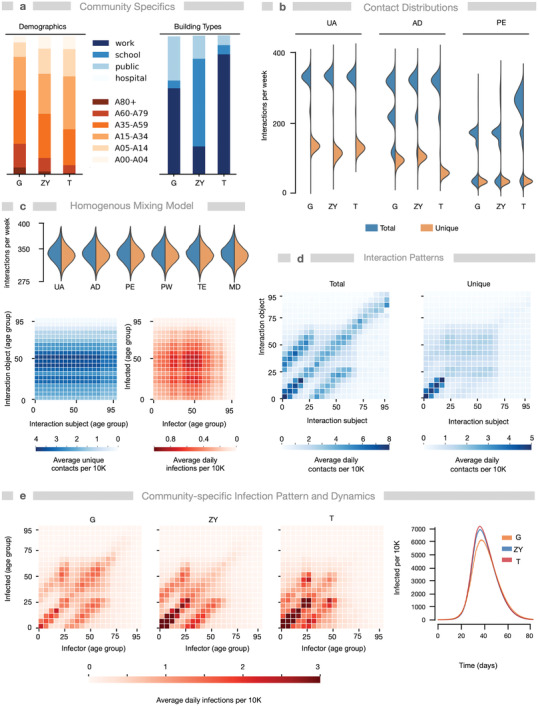
Geolocation and demographics determine interaction patterns and routes of SARS‐CoV‐2 spreading. Simulations of Gangelt (G, Germany, *N* = 10.296 agents), Zikhron Ya'akov (ZY, Israel, *N* = 17.764), and Tepoztlán (T, Mexico, *N* = 13.806) predict different infection spreads despite similar initial conditions and parameters, due to different demographics and number/types of geolocations. a) Distributions of building types (excluding homes) and age groups in G, ZY, and T. b) Distribution of average weekly total (blue) and unique (orange) interactions for underaged (UA), adults (AD), and pensioners (PE). c) Results of a homogenous mixing model (neglecting daily routines, using demographics of Gangelt) for comparison: total and unique interactions and age‐specific total interaction and infection patterns. d) Patterns of average daily unique and total contacts for G (ZY and T: Figure S13, Supporting Information). Age‐groups are 5‐year‐cohorts. e) Averaged age‐specific infection patterns and time courses for infected individuals in G, ZY, and T. Parameters: *k*_I_ = 0.14 (WT), *μ* = 2, *N* = 96 simulations.

We simulated the baseline scenario without NPIs for three communities belonging to three different continents: the German community Gangelt (G), the Mexican community Tepoztlán (T), and the Israeli community Zikhron Ya'akov (ZY), to analyze the dynamic networks and find community‐specific differences (**Figure** **2** and Section S4.4, Supporting Information). While Germany and Israel belong to high‐income countries, Mexico belongs to middle‐income countries with much lower income per capita (https://data.worldbank.org/indicator/NY.GNP.PCAP.CD?view=map). The three communities are comparable in population size, but differ with respect to their demographics (Figure 2a): age distribution, number of children, and household sizes as well as household generational overlap. In agreement, the average household size in Germany is 1.9, it is 3.2 in Israel, and 3.6 in Mexico. The communities also exhibit different geographies and composition of locations (Figure 2a).

HHIN and iHHIN are age‐ and occupation‐dependent. For the three communities (G, T, ZY), we analyzed the interactions, discriminating also between total (all encounters) and unique interactions (number of different individuals met). We found that individuals meet household members or colleagues often, while other community members potentially only once or never. Although we assumed similar interaction frequency for different age cohorts, we found that the segregation into different households together with age‐specific daily routines already structures the daily interactions of the cohorts into realistic patterns. In particular, in all three towns underaged (<20 years old) have more interactions than other groups, because they typically live in bigger households (i.e., at least with one parent) than other age cohorts (Figure 2b). Due to larger household sizes in ZY and T compared to G, corresponding to multigenerational households, adults and pensioners show more total interactions in those communities. In T, adults have less unique interactions than in G and ZY, in agreement with location‐related differences such as higher number of workplaces (Figure 2b), while public workers, teachers, and medical professionals have similar contact distributions in all three towns (Figure S16, Supporting Information). Figure 2c provides interaction and infection patterns for a homogeneous mixing scenario as comparison to appreciate the location‐ and demographics‐based effects.

Analysis of total and unique age‐specific interactions reveals different contact patterns (Figure 2d,e). Contact patterns for total interactions are dominated by the specific household structure. In contrast, the patterns for unique interactions are predominantly defined by the daily routines of the individuals and the number and size of their assigned location types such as workplaces or schools. For example, contacts between underaged at schools or adults at workplaces result in rectangular patterns of those cohorts, while inner‐household contacts result in the typical diagonal patterns^[^ ^7^, ^49^, ^50^ ^]^ representing contacts with individuals in partnerships of similar age and parent–child relationships. We potentially slightly overestimate the contacts between working adults, because we consider isolated communities and neglect commuting individuals. Since the time spent in households exceeds the time spent elsewhere, the reoccurring contacts between household members dominate the contact pattern of all interactions, but are under‐represented when considering only unique interactions. The differences in household size and location composition between G, ZY, and T are reflected in their respective contact patterns. Unique age‐specific interactions are comparable for ZY and T.

Since ZY and T have more multigenerational households than G, interactions between partners in older age cohorts are less frequent in ZY or T than in G (Figures S13 and S14, Supporting Information). Consequently, total age‐specific interactions are less pronounced in the upper off‐diagonal and stronger in the lower one, in ZY and T compared to G, that is, children and parents tend to interact more with their siblings/children than with their parents/partners. The observed unique interactions of underaged individuals and the relative interactions within their own cohort in G correspond to previous findings for contacts in German schools^[^ ^50^, ^51^ ^]^ (Figures S14 and S34, Supporting Information).

Noteworthy, age‐specific infection patterns resulting from baseline scenarios are neither completely defined by the total nor by the unique interaction patterns, but rather by a superposition of both, though with a stronger impact of the total interactions (Figure 2d,e). That shows that infection patterns are strongly coupled to household compositions. In comparison, the infection patterns (like the interactions) in homogeneous mixing are solely determined by the prevalence of different age‐cohorts (Figure 2c). Simulations of the baseline scenario with similar initial conditions lead to slightly different infection dynamics with higher AR for ZY and T compared to G (Figure 2e).

The stochasticity of HHINs are evident from the impact of an individual infection event, which may either not give rise to further infection events or further grow the network: 70% of infections originate from only 20% of the infected population and 70% of infected do not spread the infection further. This agrees with previous findings known as a possible result of overdispersion in statistical models for static interaction networks with 100% susceptibility,^[^ ^52^, ^53^, ^54^ ^]^ although we derived it from the dynamic network with decreasing amount of susceptibles, which might explain the slightly lower number.

### 2.3 Outcomes from Interventions Can Be like Flipping a Coin

We hypothesized that the stochastic nature of infections may create basins of attractions or attractor states that are highly context dependent. This would mean that even with identical initial conditions the same community may with a certain likelihood end up with opposite outcomes following NPIs or vaccination or combinations thereof.

This possibility is perhaps best illustrated in the case of airliners: The NPIs on most flights are identical (negative tests, masks, air filtering etc.). Consequently, most flights neither contribute significantly to new outbreaks nor see dramatic transmission onboard—but not all flights. There are several well‐known incidents that are due to the stochastic nature of who sits next to whom and even within the controlled environment of an airliner major transmission networks emerge as a result of the flight.^[^ ^55^ ^]^ This unique phenomenon in complex systems is known as bimodality,^[^ ^56^ ^]^ the corresponding distributions are poorly characterized by canonical statistical metrics like mean and variance.^[^ ^57^ ^]^

**Figure 3:**
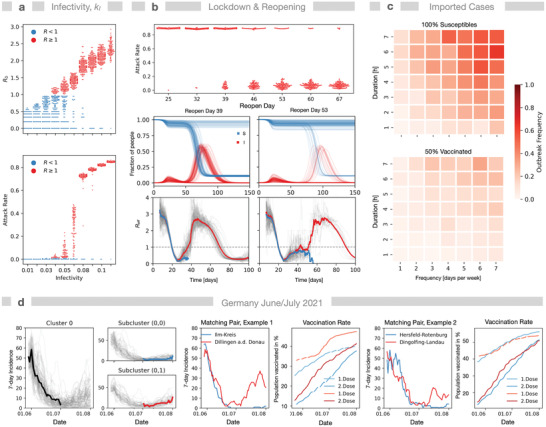
Bimodality in simulations and real world. a) *R*_0_ shows bimodality upon variation of infectivity *k*_I_, that is, same infectivity leads to *R*_0_‐values above and below 1 (baseline scenario, *N* = 96 simulations). AR also shows bimodality and high variance in range 0.03 < *k*_I_ < 0.08. b) NPI performance: Simulation of a lockdown at 18 days after first infection and a reopening. AR is given for different reopening times (25–67 days). Below: time courses for susceptible and infected individuals for days 38 and 53 (*N* = 96). Respective *R*_eff_‐time courses below discriminate between simulations with (red) and without (blue) a second outbreak; gray: all *R*_eff_‐time courses; gray dashed line: *R* = 1. *R*‐values are assigned to the infection time point of the index case. c) Robustness against commuting infectors of a community (Gangelt) with either 100% susceptible (*F*_v_ = 0) or 50% vaccinated individuals (*F*_v_ = 0). Potential infectors were introduced to a random location between 8 a.m. and 8 p.m. for different durations *τ*_i_ (1–7h) and with varying frequency *ν*_i_ (1–7 times per week) during 2 weeks while the infection dynamics was monitored for an additional 8 weeks. Heat maps show outbreak frequencies (*N* = 96 each combination). d) Indications for bimodality during the low incidence phase from June to July 2021 in German districts from serial dynamic time warp K‐Means clustering of district‐resolved time courses and subsequent matching for population density and vaccination rates. All cluster members (gray) and dynamic time warping Barycenter Average: black—before July 2021, red—outbreaks, blue—no outbreaks. Cluster 0 (1 out of 4) of the 7‐day incidence from May 29th—to July 2nd (*n*_district_ = 294 districts). Subcluster (0,0) and (0,1) from reclustering (*n* = 2) of Cluster 0 according to the incidence trend from July 2nd to August 1st, revealing districts with either stable or increasing incidence. Members of subcluster (0,1) were matched with members of subcluster (0,0) with respect to population density and vaccination rate.

Our model predicts bimodality in multiple situations: First, simulating the baseline scenario (unlimited spread of infection, neither NPI nor vaccination) we derive *R* and *R*_0_‐values. When varying the infectivity parameter *k*_I_, we find a large spread of *R*_0_ for lower *k*_I_‐values and two different modes for larger *k*_I_ (**Figure** **3**a and Figure S26, Supporting Information). Also, AR shows bimodality for varying *k*_I_. As expected, only simulations with *R*_0_ > 1 exhibit outbreaks, however, the regime of bimodality in AR extends to average *R*_0_‐values up to 2.5. For example, simulations with *k*_I_ = 0.1 and average *R*_0_‐values below 2.2, which is only slightly below the reported values of *R*_0_ = 2.79 for the ancestral strain of SARS‐Cov2,^[^ ^47^ ^]^ still did not always show an outbreak. Note that the product of the infectivity parameter *k*_I_ and the global interaction parameter *μ* is an invariant of the infection process (Figure S28, Supporting Information), hence variation of *μ* at constant *k*_I_ will show similar results. In conclusion, we find that for small incidence values *R*_0_ is not a suitable predictor for the system behavior.

A second example for bimodality also occurs for small infection numbers: after reopening of a strict lockdown of a given period *T*, we observe either outbreak termination or—the opposite—a subsequent infection wave (Figure 3b). This bimodality after lockdown lifting remains even if heterogeneity of individual contact frequency is considered (Figures S67, Supporting Information). Note that after reopening, the *R*_eff_ (time‐dependent effective *R*‐value calculated from a moving average of secondary cases) for terminated and resumed outbreaks can follow a similar trajectory for an extended period. Likewise, selective reopening of either schools, public, or workplaces after lockdown can result in bimodal behavior (Figures S23–S25, Supporting Information) at variation of reopening time points.

Up to now, we have treated the simulated community as isolated, hence the question arises whether bimodality also occurs when infected commuters enter. Thus, as a third example, we exposed either a completely susceptible population or a 50% vaccinated population (*F*_v_ = 0.5) to an infected commuting individual visiting the community within a time frame of 2 weeks with frequencies *ν*_i_ between 1 and 7 times per week for 1–7 h and monitored outbreak frequencies (Figure 3c). The outbreak frequency was never close to 1 and for lower visiting frequencies and durations *τ*_i_, the robustness against imported cases increased, hence if the system is shifted toward termination of the outbreak a low number of infected weekly commuters will not necessarily lead to a subsequent outbreak. Consequently, bimodality provides some robustness against infected commuters. Assuming that neighboring communities will have similar infection rates, that is, low frequencies *ν*_i_ of infected commuters if the incidences are low, bimodality must also be considered for infection import.

While it is impossible to directly observe bimodality in the real‐world pandemic (one can repeat simulations, but one cannot repeat “the experiment” in the same location under exactly the same conditions), we found examples that may hint to bimodality. We have analyzed the low‐incidence period in the summer 2021 in Germany by matching and cluster analysis. From January to July 2021, the incidence values in German districts (Landkreise) were comparable and dropped until early July. Afterward, some districts observed an increase while districts with similar population density and vaccination rate kept low incidence numbers for some weeks (Figure 3d). Anecdotally, New Zealand has practiced very strict policies with strict lockdowns and isolation of the entire country. In general this resulted in very low infection numbers, but several outbreaks emerged nevertheless, which potentially may be due to bimodality.

Bimodality demonstrates that effective NPIs require strict execution (stringency) and careful temporal control (timing), because the likelihood of a following wave decreases with increasing length of the lockdown. Thus, even in an ideal situation with 100% covering sentinel and surveillance data available in real‐time it might not be possible to predict the optimal strategy in low incidence scenarios without a model that captures the stochastic nature of the infection dynamics. Moreover, this challenge may scale differently across communities with, for example, different inhabitant numbers and social structure. Therefore, community‐ and location‐specificity of the epidemiological model deployed is essential for accurate prediction of adaptive and optimal strategies.

### 2.4 Quantifying the Effect of Targeted Immunization Strategies

NPIs are important to fight the COVID‐19 pandemic; however, NPIs alone have not been sufficient to stop the pandemic. We hypothesize that this, at least in part, may be due to the observed bimodality and community‐specificity of the infection dynamics. Both contribute significant uncertainty to deductions of NPI strength and duration from prior experience and outbreaks. Alternatively, targeted vaccination may be useful as a longer‐term sustainable solution to preventing further large‐scale outbreaks. Therefore, we set out to compare the performance of different strategic targeting vaccination programs.

Given that effective vaccines are available, but not for everybody at the same time, we can use the model that was trained for different communities and different NPI scenarios to analyze the effect of vaccination. Here, it is critical to define which specific objective applies when searching for optimal targeted immunization strategies for the communities.^[^ ^58^ ^]^ We analyzed four objectives: 1) minimize the number of fatalities, 2) reduce AR, 3) reduce the number of hospitalizations or individuals requiring intensive‐care treatment to prevent a health system collapse, and 4) robustness against outbreaks, that is, reaching population immunity.

**Figure 4:**
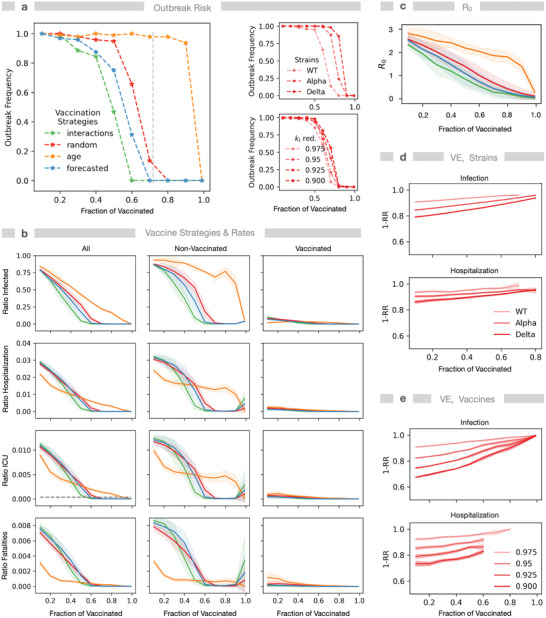
Vaccination strategies determine attack rate, population immunity threshold, death toll, and burden on the healthcare system. Compared vaccination strategies: random—individuals are selected randomly, forecasted—individuals are selected according to their infection order in a previous simulation, interaction—selection by descending interaction frequency, age—selection by descending age. We assume vaccines are ≈90% effective (infection and hospitalization probability reduced by 97.5% and 20%), if not stated otherwise, and analyze effects for a partially vaccinated population (0–100%). a) Ratio of simulations with identical conditions exhibiting an outbreak. Left: comparison of strategies. The dashed gray vertical indicates the theoretical population immunity threshold for *R*_0_ = 2.83 and VE = 0.9. Right: effect of strains (WT, Alpha, and Delta) and of vaccine efficacy (*k*_I_ reduction as shown) for the random strategy. b) Impact of newly introduced infections: a fraction of the population selected by the named criteria was set to immunized, then four new infections were introduced. Shown are AR, the hospitalization rate, the ICU demand (horizontal dashed line: capacity assuming the German number of ≈30.000 per 82 million inhabitants) and the fatalities either for the whole population (left column) or for nonvaccinated (middle) and vaccinated (right). c) Resulting *R*_0_‐values for different strategies and degrees of vaccination. d) Vaccine efficacy: Effect of virus strain infectivity on risk reduction due to vaccination (1‐RR) shown for infection and hospitalization numbers. e) Effect of *k*_I_ reduction on vaccine efficacy (1‐RR) for infection and hospitalization numbers for the random strategy. All simulations are for the German municipality Gangelt with 10.351 agents. Parameter values: *k*_I_ = 0.15, *μ* = 2. *N* = 96 simulations each.

We simulated six vaccination strategies (**Figure** **4** and Figures S40–S51, Supporting Information), where individuals were either prioritized by descending age, by descending interaction frequency, according to their infection time in previous unaltered simulations (forecasted) or not sorted at all (random). Additionally, we defined a combined strategy, where first individuals above age 60 were vaccinated in the order of descending age and, second, thereafter the most interactive individuals were prioritized. In the household strategy, first one person per household gets vaccinated (ordered by descending age), then a second person and so forth.

The effect of vaccination on individuals is modeled by reducing infection and hospitalization probability by 97.5% and 20%, resulting in a vaccination efficacy (VE) (1‐RR, reduction in risk due to vaccination) of 0.88–0.98 (Figure S40, Supporting Information), for 10–60% vaccination fraction, in agreement with reported values for the BioNTech, Pfizer vaccine against WT and the Delta variant.^[^ ^59^, ^60^ ^]^ Additional vaccination models were tested (Section S4.8.3, Supporting Information) exhibiting similar outbreak frequency.

The strategies were compared, first, with respect to the outbreak risk (objective 4) when introducing four infections into a partially vaccinated population (*F*_v_ ∈ (0, 1), Figure 4a). The outbreak risk was defined as the ratio of simulations exhibiting an outbreak (*R*_0_ > 1) to all simulations with identical parameters. This outbreak risk vanishes at the theoretical herd (population) immunity threshold (HIT/PIT) (4a), if vaccinations follow no particular prioritization (random). For other vaccination strategies, the required fraction of vaccinated, that is, the PIT, can shift either to lower values (interaction) or to higher values (age) than PIT. Importantly, vaccination according to interactions performs best for maximal reduction of the outbreak risk, while vaccination by age hardly reduces the outbreak risk.

For the random strategy, we analyzed the effect of the strain (WT or VOC) and different levels of efficacy (*k*_I_ reduction) for the WT (Figure 4a). Both VOC and reduced efficacy lead to higher outbreak risks in a certain range of the fraction of vaccinated.

Next, we assessed the vaccination strategies with respect to their ability to reduce AR (objective 2) and the ratios of hospitalized, ICUed (objective 3), and dead individuals (objective 4). Here, we considered either all individuals or discriminated nonvaccinated and vaccinated individuals (Figure 4b). For high vaccination levels, the strategy to prioritize the most interactive individuals is optimal, as it reduces the outbreak risk most efficiently, thereby preventing all states subsequent to infection, that is, it serves all four objectives simultaneously. For lower percentages of vaccinated individuals, we identify a tradeoff between different strategies depending on the objective, that is, infection wave attenuation, preventing fatalities, or avoiding ICU overload. Aiming to minimize infection numbers is similar to reducing the outbreak risk, where it is still most effective to prioritize the most interactive individuals, as it increases systemic robustness. It even outperforms vaccination by forecasting of infected individuals in a presimulated baseline scenario. Random vaccination underperforms compared to the other two strategies, also independently shown by others,^[^ ^8^, ^61^ ^]^ but outperforms sorted‐by‐age vaccination, when aiming for outbreak risk reduction. This is supported by the fact that vaccination by descending age is not as efficient in reducing *R*_0_‐values, that is, slowing down the infection dynamics (Figure 4c), rendering the strategy of prioritizing by age least effective at high vaccination levels, since this strategy is not able to suppress deaths completely before full population immunity is reached. A reason could be that younger individuals keep interacting, leading to high connectivity among the remaining susceptibles. In general, individuals of the same cohort form subnetworks that remain unperturbed by vaccination of members of other cohorts.

In agreement with other recent studies, we find that the age‐sorted strategy is very effective to minimize fatalities at lower vaccination levels^[^ ^62^, ^63^, ^64^ ^]^ and is only slightly outperformed by the combined strategy (Figure S42, Supporting Information). The combined strategy integrates the two strategies that either best reduce infections (i.e., by interaction) or death toll (by age) and can outperform both strategies, but only slightly and at medium vaccination levels and only with respect to hospitalization and ICU admission (Figure S41, Supporting Information).

The simulations also reveal a problem of strategies that focus on vaccination only (Figure 4d): with the objective to reduce ICU occupancy, vaccination by age performs best at less than ≈40%, but vaccination by interactivity is best above this level, considering infection with WT. However, below ≈50% vaccination, none of these strategies is able to prevent overload of ICU capacity, without additional NPIs. Furthermore, outbreak frequencies or hospitalization rates are increased for more infectious strains. Importantly, our model did not implicitly include an increased death rate if ICUs are overloaded. Hence, the death toll would be even higher than predicted if ICU demands cannot be met. While ICU capacities may vary in different locations, the problem remains that ICU demand and capacity differ widely for all strategies at stages of partial vaccination. This implies that NPIs remain to be considered to accompany the vaccination process in order to prevent the collapse of the healthcare system.

Thus, our model simulations suggest that, as long as one cannot ensure vaccination levels that minimize outbreak risks, which depends on the deployed strategy and VE, it is not possible to satisfy all objectives to reduce deaths, ICU demand, and infection levels at the same time equally well only with means of vaccination.

Although immunity waning was not explicitly considered for the analysis of vaccination strategies, as it is implicitly captured by reduction of VE,^[^ ^65^ ^]^ immunity waning will increase the immunity threshold even further.

The prevalence of different strains also has an influence on infection and hospitalization rates (Figure 4d and Figure S53, Supporting Information) as has the efficacy of the vaccine shown here for different values of reduction of *k*_I_ (Figure 4e). Considering different strains modifies the quantitative, but not the qualitative outcomes of the different vaccination strategies (Figures S46 and S50, Supporting Information).

We observe bimodality also for vaccination, that is, systematic vaccination of the population can lead to either termination or recurrence of outbreaks with a certain likelihood—depending on the strategy in the range of between 40% and 90% people vaccinated (Figure 4a). This means that in these ranges—either periods of time or sets of NPIs or ratios of vaccinated individuals—it depends essentially on luck whether a community will experience another wave. This necessitates adapting and 1) using precision simulations to see whether a community is in such a range or if the situation is controlled, and 2) combine vaccinations with NPIs in order to simultaneously keep the outbreak down and ease the conditions for the population by preventing unnecessary lockdowns or school closures.

In line with independent reports,^[^ ^17^, ^39^ ^]^ we could not identify a unique value for the critical fraction of vaccinated individuals required for population immunity. Instead, this value depends on the chosen strategy, the heterogeneity of the population, the infectivity of the virus, and the vaccine efficacy (Figure 4a). In particular, lower infectivity will decrease the required vaccination coverage to achieve population immunity (Figure S39, Supporting Information).

### 2.5 Aiming for Population Immunity and Control of Outbreaks Require Adaptive Combinations of NPIs and Vaccination Strategies

**Figure 5:**
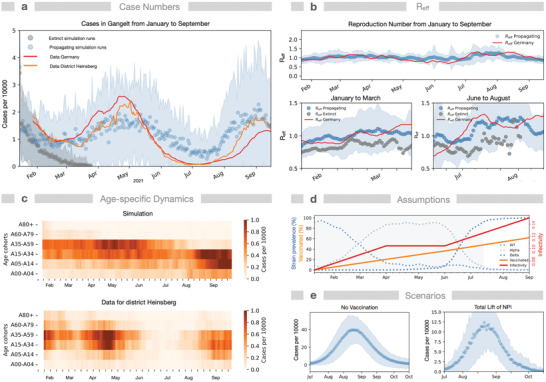
Comparison of adaptive interventions with real‐world epidemiological data. Simulations combining NPIs and vaccinations as applied in Germany are contrasted with the infection dynamics in district Heinsberg, enclosing Gangelt, and the whole of Germany for the period from January 15, 2021 to September 15, 2021. Simulations started with 117 infected individuals (corresponding to the incidence numbers on January 15th) and drastically reduced contacts (by 75% at public places, by 75% at schools, by 40% at workplaces) representing the applied NPIs (lockdown). NPIs were relaxed to an overall contact reduction of 40% on August 3rd. a) Simulated daily new cases of diagnosed individuals, exhibiting bimodality by showing either a propagating infection wave (blue; mean +/− SD) or extinction of infection (black, mean +/− SD, 137 out of 384 simulations) after the initial decline in cases. For comparison, data for Heinsberg (orange) and Germany (red, 7‐day‐sliding window scaled to the population size of Gangelt) are displayed.^[^ ^66^ ^]^ b) Evolution of *R*_eff_: simulation only for propagating infection waves, estimated numbers for Germany.^[^ ^69^ ^]^ Lower panels: *R*_eff_ for propagating and extinct infection waves prior to increases in diagnosed cases in March and August. c) Comparison of simulated diagnosed new cases within age cohorts with data for district Heinsberg. d) Underlying assumptions: The infectivity increases in a piecewise linear fashion, reflecting empirical prevalence of the wild type, Alpha, and Delta strains.^[^ ^67^ ^]^ Vaccination roll‐out *v*_s_ is linear according to age at a rate of 28 daily immunizations, reflecting the average vaccination progress in Germany (from 0% fully vaccinated in January to 60% at the beginning of September).^[^ ^68^ ^]^ The simulated period of stronger NPI is indicated (gray shade). e) Comparison of outbreak magnitudes for the period July 15th to October 15th for scenarios assuming either no vaccination or total lift (0% contact reduction) of NPIs in July. *N* = 384 simulations.

To demonstrate the model capabilities for real‐world data, we compared the simulated course of infection in the period January–September 2021 in the municipality Gangelt to real data from the enclosing district Heinsberg and from Germany (**Figure** **5**).^[^ ^66^ ^]^ Here, we considered 1) the local incidence values in early January 2021 according to RKI data, 2) the change in prevalence of WT and the Alpha and Delta variants of the virus, 3) the valid NPIs, and 4) the increasing immunization rate (2nd vaccine dose) in Germany (Figure 5d).^[^ ^67^, ^68^ ^]^ We modeled vaccination according to age as described above since this best matches the situation in Germany during that period. Under these conditions, the simulations again exhibit bimodality (subsiding infections in 35% of simulations, a new infection wave in April otherwise). The dynamics of the 7‐day rolling average of reported daily new cases matches the rolling average of the simulated newly diagnosed individuals (Figure 5a) and the relative occurrence in different age cohorts (Figure 5c) in simulations showing a new infection wave. Moreover, even the value, tendency, and weekly oscillation of the derived *R*‐value (Figure 5b) agree with reported estimates for *R*.^[^ ^69^ ^]^

Note that some aspects not considered here could influence the real‐world trajectory. The assumed roll‐out strategy age is an idealization of the strategy applied in Germany, leading to a strong reduction of infections in the cohorts 60+. Data show that also vulnerable individuals of other age groups and health care persons were vaccinated. The increasing rate of testing, the potential seasonal dynamics of the virus,^[^ ^70^ ^]^ and the commuting of infected individuals to other communities may also play a role. Using the model with infectivity adjusted to the real course of infection in the period January–September 2021, we can demonstrate that the combination of NPIs and vaccination is required to keep case numbers low: case numbers strongly rise in simulations either without vaccination or with a total lift of NPIs in July (Figure 5e). Considering additionally waning immunity shifted the onset of the autumn wave to earlier dates (Figure S63, Supporting Information). In summary, both NPI and increasing vaccination numbers were relevant in keeping the infection wave under control.

## 3 Discussion

Vaccination of the human population against COVID‐19 is an immense logistical challenge that necessitates careful prioritization in order to swiftly reach maximal suppression of the disease and also save lives.^[^ ^71^ ^]^ Limits on production and distribution render it absolutely necessary to prioritize and structure the vaccine deployment.^[^ ^58^, ^72^, ^73^ ^]^ The European Commission concluded that “the successful deployment and a sufficient uptake of such vaccines is equally important” rendering it critical to be able to “monitor the performance of the vaccination strategies.”^[^ ^74^ ^]^ Thus, the challenge for the scientific community is to develop platforms that can provide precise, context specific and adaptive predictions of the quantitative effect and requirements of such strategies in close to real‐time. Several well‐established simulation frameworks exist^[^ ^75^, ^76^, ^77^, ^78^, ^79^ ^]^ and have been successfully applied or adapted to explore SARS‐CoV‐2 pandemic behavior and intervention effects (e.g., refs. [18, 28, 29, 30]). We aimed for combining the properties to semi‐automatically integrate geographic, demographic, and virological open source data in order to simulate consecutively and comparatively, and to record simulations in full detail. Therefore, we have responded to the challenge described above by developing data‐driven geospatial, temporal models of integrated individual human behavior together with the required simulation environment. This made it possible to quantify effects of NPIs and compare the impact of targeted immunization strategies. Remarkably, the heterogeneous model offers insight into the bimodal behavior of SARS‐CoV‐2 infection dynamics and demonstrates that effective interventions require stringency and careful temporal control. We demonstrate that COVID‐19 iHHIN are sparse and small compared to the overall HHIN. Therefore, models based on homogenous mixing or averaging statistical models are likely to be of limited use^[^ ^25^, ^26^ ^]^ especially during phases of low to moderate infection or emergence of new variants, since they fail to capture heterogeneity and complexity emerging from stochastic and sparse events influenced by individual human behavior.^[^ ^16^, ^32^, ^80^, ^81^ ^]^ In future, hybrid models could potentially combine the required depth w.r.t. human behavior and breadth w.r.t. global impact.^[^ ^82^ ^]^

Our work demonstrates a tradeoff between different strategies for low levels of vaccination: vaccination by age minimizes fatalities, while vaccination by interactivity reduces infection events. However, at high vaccination coverage, vaccination by interaction prevails. It is important that the vaccination level giving rise to population immunity is not a unique number but depends on the chosen vaccination strategy. These conclusions depend on the demographics and the heterogeneity in the interactions and it can be assumed that the stronger the heterogeneity the better vaccination by interaction will perform.

Targeted immunization “into” an ongoing outbreak may still become relevant. This approach likely performs differently to other strategies, since infection spreading might already reach the vulnerable subgroups and spread further in these subnetworks. Vaccination of the population is a process in time, especially in the global context. But locally, significant vaccination coverage has already been achieved in some countries or regions. The optimal vaccination strategy depends on the supply and acceptance of vaccines, the demographic structure, local behavioral costumes, and the capacity to realize a specific strategy. Although population immunity is unlikely to be reached for the Delta variant or even more infectious VOC,^[^ ^39^ ^]^ we found that vaccination by interaction would reduce the population immunity threshold, at least for Delta to less than 80%, a value which seems achievable. In future, forecasting of the effect of vaccination shall be combined with prior simulation of the ongoing surge of infections and the effect of hitherto applied NPIs to precisely model the situation in specific communities at the time when vaccines become available.

Here, we showed that the particular demographics and the distribution of locations inside a specific community shape the contact dynamics and, hence, the infection dynamics. These emergent community‐specific patterns provide insights as a basis to derive strategies to efficiently control and prevent outbreaks. The presented approach can be used for further intensive comparisons of the effects of NPIs in different communities or demographic settings.

Our results suggest that, in order to reach a post‐COVID world, communities and governments worldwide will have to deploy adaptive and real‐time‐based simulation support for decision making. This is the only path that can ensure continued education, economic and research activity, healthcare, and other society functions during new outbreaks by minimizing the catastrophic socio–economic impact of lockdowns, travel bans, and civil noncompliance.

To fully achieve this, new paradigms for modeling of infection networks that capture the complexity and stochasticity of human behavior will be important beyond what we have demonstrated here. For example, considering the needs of individuals as a basis of autonomous decision making instead of preset daily routines, as done by Dignum, et al.,^[^ ^29^ ^]^ would enhance model complexity and allow for analyzing individual responses to NPIs. However, calibrating such models poses a daunting task and will drastically increase the demand of comparable data, thereby reducing the worldwide applicability of the model.

## 4 Experimental Section

The authors designed an open source simulation platform for GEoReferenced Demographic Agent‐based (GERDA) modeling of virus propagation using Python. To simulate infection transmission and disease progression between individuals, the modeling framework automatically generated a social contact ABM based on geographic and demographic input data. In addition, the classical SIR model was expanded with infection substates such that the model contained 7 health states and 13 state transitions. The modular design, separating initialization, simulation, and analysis, assured the support of different detail levels for user‐adjustable input data, comparable simulations, and comprehensive simulation outputs.

Below, the main concepts of model design, data integration, model initialization, and model simulation are described. The details of GERDA, including parameter estimation for stochastic state transitions and additional simulation results are represented in Supporting Information. **Table** **1** provides a list of key model parameters, while an extended summary of parameters and other model entities is listed in Table S1, Supporting Information.

**Table 1:** Model and simulation parameters

Symbol	Parameter	Scope	Type
*μ*	interaction frequency	global	interaction
*x_a_*	interactivity of agent *a*	individual	interaction
$P_{ijt}^{X}$	transition probability of transition *X_i_* → *X_j_* at time *t*	individual	infection progression
*φ*_*i*,*j*_	health state transition modifier	individual	infection progression
$I_{a}^{I}\left(t\right)$	infectiousness of agent *a* at time *t*	individual	infection transmission
*k*_I_	infectivity	global	infection transmission
*n*_I_	initial infections	global	environment
*ν*_i_	import frequency	global	environment
*τ*_i_	import duration	global	environment
*t*_start_	lockdown onset	global	policy
*t*_end_	lockdown lift	global	policy
*F*_c_	noncompliance fraction	global	policy
*F*_v_	vaccinated fraction	global	policy
*v*_s_	vaccination roll out speed	global	policy

### Model Design Principle

In this ABM, each human individual (agent) was associated with a defined physical location at each time point. These locations were specific for the community as they correspond to actual buildings retrieved from OSM^[^ ^83^ ^]^ which were assigned to one of the following categories: homes, workplaces, schools, hospitals, and public places (Figure 1c). Since transmissions outdoors or in public transport were of less importance,^[^ ^84^ ^]^ they were not explicitly included.

*Agent attributes*: Individuals were characterized by individual attributes such as a unique ID, a home and an associated household, an age (to impose age‐specific disease history), health‐state transition modifiers *φ*_*i*,*j*_ (e.g., to represent vaccination), a specific interactivity modifying contact rates, and an (active) weekly schedule governing its time‐dependent movements between locations in the simulated environment (Section S2.2.1, Supporting Information). The health states for individuals were defined as: susceptible (S), infected (I), recovered (R), or deceased (D). Substates of infected individuals (I) comprised: pre‐ or asymptomatic (plain I), diagnosed (I^d^), hospitalized (I^d^_H_), or admitted to ICU (I^d^_ICU_) (Section S2.2.6, Supporting Information).

*Agent behavior (movement)*: In every simulated time step each individual moved to the location specified in its active schedule for this time. The schedule was either followed or was altered by the individual agent's health state and potentially imposed NPIs, considering the compliance of individuals to follow them (Section S2.2.2, Supporting Information). While NPIs hold, represented by closure of certain location types, individuals stayed at home instead of visiting those locations. If individuals assumed the infection substates I^d^, I^d^_H_/I^d^_ICU_, or D their active schedules were changed to visiting either their home, hospital, or morgue 24/7. Upon lift of NPI or change of the state to R, the individuals’ schedules reset.

### Data Integration and Model Initialization

All data were taken from publicly available databases,^[^ ^43^, ^44^ ^]^ including location information (e.g., from www.openstreetmap.org) and population information (derived from country‐specific census databases). For the infection process, the authors' integrated information from the German Robert Koch Institute (RKI) factsheets^[^ ^85^, ^86^ ^]^ and from recent publications^[^ ^45^, ^46^ ^]^ to derive hourly conditional probabilities for transitions between health states.

*World Generation*: An environment, representing the modeled community, was generated from annotated geo‐data. It comprised locations with geographic coordinates of a specific location type, based on annotations. Unannotated locations were added to the residential spaces, serving as homes (Section S3.1, Supporting Information).

*Agent Generation*: For every home a household of individuals was generated based on the country‐specific demography (age distributions and household composition) (Sections S3.2 and S3.3, Supporting Information). Each generated individual was assigned a weekly schedule depending on its age and, if not stated otherwise, initialized as susceptible *S*.

*Schedule Generation*: Unique weekly schedules (hourly resolution, discriminating between weekdays and weekends) specified the individual's presence at different locations at different times (Figure 1c). The schedules were generated from templates, resembling typical daily routines of individuals represented by the respective agent‐type. The specific locations visited by an individual at a given time were chosen randomly from all locations of the dedicated type, with decreasing probability for increasing distance to the individual's home. The precise changing times between locations were determined by adding uncertainty around the boundaries of the respective time slot (Section S3.3.2, Supporting Information).

*Setting Initial Seed Infections*: The spread of infections started with setting a number of individuals to infected *n*_I_. To avoid early termination of infection spreading (Figure S27, Supporting Information), the authors used four seed infections as default. For better comparability of the simulations the same four initial infections within one household were used for all simulations, unless stated otherwise.

### Model Simulation

The simulation ran for a specified number of time‐steps. At each time step the individuals performed a specific set of actions in following predefined order. 1) All individuals defined whether their disease state would change according to given time dependent transition probabilities of the substates$P_{\mathrm{ijt}}^{X}$, defined in the SIR model (Section S2.2.7, Supporting Information). 2) For each location the interacting pairs of individuals were determined and infection transmission from an infected to a susceptible individual was decided. The likelihood of each individual to engage in interactions was defined by the specific interactivity (*μ*) (Section S2.2.4, Supporting Information). Infection events depended further on the time‐dependent infectiousness of the potential infector $I_{a}^{I}\left(t\right)$, the susceptibility of the potentially infected as well as on the general infectivity parameter *k*_I_ (tunable according to NPI such as mask wearing and adjusted to the reported *R*_0_ for different virus strains) (Section S2.2.5, Supporting Information). Vaccination was implemented as modification of state transition probabilities for infection, diagnosis, hospitalization, ICU admission, and death (Section S4.6.3, Supporting Information). 3) The current state of each individual was recorded and the individuals underwent the state transitions (between health states), defined in (1) and (2). Finally, the individuals either moved to a new location or stay, according to their active schedule. All individuals simultaneously present at a given location (colocation network) could interact with each other, establishing the HHIN and infection‐transmissions the iHHIN.

Sensitivity analysis on the impact of free parameters *k*_I_, *μ* as well as initial infections and the timing and type of lockdown interventions is presented in the Section S4.6, Supporting Information.

## 5 Code Availability

GERDA is a free software, available at our GitLab repository (https://tbp-klipp.science/GERDA-model/) and published under GNU General Public License v3.0. The repository also contains a manual for the application of the method to other communities, including the required types of data.

## Conflict of Interest

The authors declare no conflict of interest.

## Author Contributions

B.G., S.O.A., and O.B. (joint first authors), and J.A.H.W. and X.E.‐F. (joint second authors) contributed equally to this work. Conceived the project: R.L. and E.K. Conceptual work on the model: B.G., S.O.A., O.B., J.A.H.W., X.E.‐F., A.K., R.L., and E.K. Programming: B.G., S.O.A., O.B., J.A.H.W., A.K., L.B., J.E.L.H., M.K. (Karnetzki), R.M.‐T., and M.S. Analysis and computational experiments: B.G., S.O.A., O.B., J.A.H.W., X.E.‐F., A.K., M.K. (Karnetzki), R.M.‐T., M.S., and E.K. Data collection: X.E.‐F., J.E.L.H., M.K. (Krantz), L.M., H.P., and P.S.S. Wrote the paper: B.G., O.B., J.A.H.W., X.E.‐F., M.K. (Krantz), M.S., R.L., and E.K. All authors agreed on the final version of the manuscript.

## Supporting information

Supporting Information

Supplemental Tables

Supplemental Movie 1

## Data Availability

The data that support the findings of this study are available in the supplementary material of this article.
