# Supplementary material for: Control of COVID‐19 Outbreaks under Stochastic Community Dynamics, Bimodality, or Limited Vaccination
Source: Adv Sci (Weinh). 2022 May 23;9(23):2200088. doi: 10.1002/advs.202200088 (PMC9348421; doi:10.1002/advs.202200088)
Supplement: Supplementary file 1 — Supporting Information [file ADVS-9-2200088-s003.pdf]

ADVANCED SCIENCE
Open Access

Supporting Information

for *Adv. Sci.*, DOI 10.1002/advs.202200088

Control of COVID-19 Outbreaks under Stochastic Community Dynamics, Bimodality, or Limited Vaccination

*Björn Goldenbogen, Stephan O. Adler, Oliver Bodeit, Judith A. H. Wodke, Ximena Escalera-Fanjul, Aviv Korman, Maria Krantz, Lasse Bonn, Rafael Morán-Torres, Johanna E. L. Haffner, Maxim Karnetzki, Ivo Maintz, Lisa Mallis, Hannah Prawitz, Patrick S. Segelitz, Martin Seeger, Rune Linding and Edda Klipp**

Control of COVID-19 outbreaks under stochastic community dynamics, bimodality or limited vaccination

Björn Goldenbogen[1$], Stephan O Adler[1$], Oliver Bodeit[1,2,3$], Judith AH Wodke[1§], Ximena Escalera-Fanjul[1§], Aviv Korman[1], Maria Krantz[1,b], Lasse Bonn[1], Rafael Morán-Torres[1], Johanna EL Haffner[1], Maxim Karnetzki[1,a], Ivo Maintz[1], Lisa Mallis[1], Hannah Prawitz[1], Patrick S Segelitz[1] , Martin Seeger[1,4], Rune Linding[1,4], and Edda Klipp[1,4,*]

[1] Humboldt-Universität zu Berlin, Theoretical Biophysics, Invalidenstr. 42, 10115 Berlin, Germany

[2] Institut für Biochemie, Charité – Universitätsmedizin Berlin, Berlin, Germany

[3] Institute of Quantitative and Theoretical Biology, Heinrich-Heine-Universität, Düsseldorf, Germany

[4] Rewire Tx, Humboldt-Universität zu Berlin, Invalidenstr. 42, 10115 Berlin, Germany

[$] These authors contributed equally (joint first authors)

[§] These authors contributed equally (joint second authors)

* Correspondence to: edda.klipp@hu-berlin.de

Supplementary Material

Supplementary Material

1 Introduction

2 Model Design

2.1 Locations

2.1.1 Location Types

2.2 Agents

2.2.1 Agent Properties

2.2.2 Agent Operations - Schedules and Occupation

2.2.3 Motion Between Locations According to Schedules

2.2.4 Agent-agent Interactions

2.2.5 Infection Transmission

2.2.6 Health States

2.2.7 Health State Transitions

3 Model Initialization and Data Integration

3.1 Location Initialization

3.1.1 Principles of Location Initialization

3.1.2 Data used for Location Initialization

3.2 Household Initialization

3.2.1 Principles of Household/Population Initialization

3.2.2 Data for Household Initialization

3.2.2.1 Conversion of public data into GERDA input tables

3.2.2.2 Algorithm

3.3 Agent Initialization

3.3.1 Principles of Agent Initialization

3.3.2 Schedule Generation for Agents

3.4 Health State Transitions

3.4.1 Principles

3.4.2 Input Data for Health State Transition Rates

3.4.3 Probability Calculations

3.4.3.1 Age-independent state transition probabilities

3.4.3.2 Age-dependent state transition probabilities

3.4.3.3 Time-Dependent Conditional State Transitions

3.5 Infectiousness of an infected agent

3.6 Additional Parameters

4 Simulation and Analysis of GERDA

4.1 Simulation time course and outputs

4.1.1 Simulation object at final time step

4.2 Simulation of specific scenarios

4.3 Duration between agents' state transitions

4.4 Community specificity

4.5 Bimodality

4.6 Sensitivity analysis

4.7 Network analysis

4.7.1 Interaction networks

4.7.2 Infection networks

4.7.3 Overrepresentation and underrepresentation of specific groups in the infection process

4.8 Immunization scenarios

4.8.1 Vaccination strategies

4.8.2 Impact of different strains on vaccination scenarios

4.8.3 Vaccination effects

4.8.4 Vaccine efficacies

4.9 Comparison to Homogeneous Mixing Approach

4.10 Exemplary application to other communities

4.11 The movie

5 References

1 Introduction

Severe acute respiratory syndrome coronavirus 2 (SARS-CoV-2) has been globally affecting societies and economies for about two years and classical strategies have not been successful to prevent further spreading of the virus. To support evidence-based decision making during this ongoing crisis, the scientific community has made great efforts to analyze the available data (selected reviews:[1–5]), to predict virus propagation and the influence of different societal aspects, such as age or contact levels[6–8], to analyze the underlying networks of outbreaks[9], and to provide scientific evidence for the efficacy of different mitigation measures, testing or vaccination strategies (selected reviews:[10–12]). With the Global Epidemic and Mobility (GLEaM) framework there is a stochastic computational scheme that integrates worldwide high-resolution demographic and mobility data to simulate disease spread on the global scale[13]. Still, in a recent comment, Bouffanais and Lim pinpointed the discrepancy between the detail levels of available data and mathematical models versus the requested and required information to effectively prevent uncontrollable virus spreading[14].

Classical SIR-models[15,16] are commonly based on population-wide assertions, thereby omitting the variability of the infection transition process between individuals. Traditional agent-based models[17] capture stochasticity in agent behavior, but are highly limited in size by computing capacities. Others have attempted to overcome these limitations: Already in 2005, Ferguson et al.[18] published an agent-based model considering statistical distributions of transmission events in households, building types and alike, while Chao and coworkers considered data on personal contacts back in 2009[19]. More recently, Gomez et al.[20] simulated infection transmission throughout Bogotá representing the city's population by 1000 agents and Lai et al.[21] created a simple model to analyze the effect of traveling between cities in China, but without realistic individual agents. Quilty et al.[22] predicted quarantine and test strategies. Still, they all attempt to describe the infection process on population level, thus relying on a coarse-grained representation by a limited number of agents. Alternatively, Kissler et al.[23] developed an ODE model that describes increased social distancing by decreasing the basic reproduction number (R value). Ferretti and coworkers[24] modeled presymptomatic and non-diagnosed transmissions, Zhang et al.[7] provided valuable age specific data on contact patterns in the Chinese population, and Karin et al.[8] assessed the effect of alternative working schedules. Others estimated the rate of detection of symptomatic cases of COVID-19 in France after lockdown and suggested school reopening scenarios using data coupled with mathematical transmission models[25,26]. Mistry et al.[27] derived contact matrices to model virus spread and show that sub-national heterogeneities in human mixing patterns, however they employed ODE whereby they neglected the derived detailed household structures again. Also Quilty et al.[22] derived contact patterns of UK adults before and during lockdowns from questionnaires[22].

While certainly all of those models administer important information for decision processes during a pandemic, they still focus on population dynamics, i.e. the macroscopic view, and consider only specific aspects of the virus transmission process. In contrast, virus outbreaks are fundamentally non-linear, stochastic, and highly network-based. Additionally, the propagation between human individuals depends on the specific geospatial and demographical context. Consequently, quantitative assessment of non-linear integrative effects might be key to developing suitable intervention and vaccination strategies[6,14,28], or even account for social needs but do not include geographic or demographic data[29]. To efficiently predict virus propagation on a more specific, not necessarily global level, we require heterogeneous models that take into account not only age cohorts and social activity levels but many other types of heterogeneities such as households (of different sizes) or different types of locations, like schools or workplaces[6].

Several ABM simulators are available that are or could be applied for SARS-CoV-2 transmission simulations, for example proprietary AnyLogic[30], which, unfortunately, even for academic use is not free of charge. In addition, open source tools like Uri Wilenski's NetLogo[31], a commonly used ABM modeling environment written in Scala language, repast[32], a Swarm-based Toolkit with implementations in several languages and built-in adaptive features, or MATSim[33] a transport simulator, are valid alternatives. However, all of those simulators have one or more shortcomings, either with respect to options for data incorporation, parallel computing, expansion possibilities, or to software licensing, when considering the desired data-driven level of detail for simulation and analysis. More recently, new COVID-19-related simulation tools and models have been published, such as Episim, a tool box from Kai Nagel and colleagues[34] that is based on MATSim, ACEMod[35], an adaptation of an influenza ABM for Australia-wide simulation of COVID-19, INFEKTA an epidemiological model based on AnyLogic[20], and Covasim, a very detailed, demographic data-based COVID-19 model with individual agents that is used to support guided decision making in different countries[36].

To complement and improve those approaches with respect to options for data incorporation, parallel computing, and expansion possibilities, we developed a GEoReferenced Demographic Agent-based (GERDA) model. GERDA uses an integrative concept for modeling of virus spreading that focuses on individual virus transmission events in a realistic populated community created based on detailed geographic and demographic data. To this end, we used an expanded SIR model and extended it with information on heterogeneity in the population. GERDA allows simulation of a multitude of consecutive and comparative scenarios providing locally specific information on infection hubs and a variety of population subgroups. Showing bimodal simulation outcomes for the same scenario proves GERDA's capability to capture the stochasticity of the virus transmission process. Comparative simulations allowed us to evaluate the efficacy of different vaccination strategies. Finally, the ability to adapt simulation scenarios to the current situation in real-time, the potential applicability of the approach to any virus and to communities world-wide, and the capability to deal with various levels of detail for the different input data, render this approach highly valuable in the current but also in future pandemics. The model entities, parameters, and other important symbols used throughout this document are summarized in **Table S1**.

**Symbol**	**Name**	**Detail**	**Property/Dependency**	**Type**	**Reference**
MW	Modeled world	Reflects real-world community/town, composed of locations and agents	Custom	Model object	
loc	Location	Container for agents with geographical coordinates, reflects, real-world building or open space	workplaces, schools, public places, hospitals, morgue, and households or mixing location	Model entity	OpenStreetMap (OSM)
	Agent	Reflects human individual	agent ID, home location, age, schedules, health state	Model entity	https://ergebnisse.zensus2011.de
n_{loc}	Agents per location	Number of agents present at a specific location at the same time	Location type, location size, time, schedules	Time dependent simulation output	
μ	Global interaction frequency	Mean interaction frequency of agents		Input parameter	Wallinga et al., 2006
P^{int}_{ab}	Interaction probability	Interaction probability of two agents a and b per time step at specific location	M, n_{loc}, x_a , x_b and μ	Emerging parameter	

M	Interaction matrix	Matrix coefficients specify the probability with which any pair of agents may interact at a given location and time (x_i x_j)	Interactivity, schedule, location	Emerging parameter	
k_I	Global infection	Reflects the overall infectivity	User dependent	Input parameter	
P^{inf}_{ab}	Infection probability	Probability of an infected agent to transmit the virus to a susceptible interaction partner	Age, infected agent time of infection	Input parameter	He et al., 2020
X_{ij}	State transition	Infected agents undergo various health state transitions reflecting disease progression until they either recover or die. X_{ij}, $S_i \rightarrow S_j$ with S_i, S_j being health (sub-)states	Si health (sub-)states, state transition probabilities P^X_{ijt}	Model entity	
P^c_{ij}	Cumulative transition probability	Transition probability, with i, j being health (sub-)states.		Input Parameter	RKI Factsheet; Karagiannidis et al., 2020
P^X_{ijt}, P^X_{ijT}	Transition probability	Time and age dependent gaussian distributed transition probabilities with i, j being health (sub-)states and time t		Input Parameter	
p^X_{ijt}, p^X_{ijT}	Conditional transition probability	Time and age dependent conditional transition probabilities with i, j being health (sub-)states and time t		Input Parameter	
	Infectivity distribution	Poisson distribution reflecting infection time dependent differences in infectivity	Time of infection	Input Parameter	He, et al., 2020
t, T	Time	Discrete time step (per hour)			Mossong et al., 2008
S or S_1	Susceptible	Default state. Agents have never been subject to the infection and neither are immune to it	Simulation, state transition probabilities	Health state	
I or S_2	Infected	Comprise presymptomatic, asymptomatic and symptomatic infected individual	Simulation, state transition probabilities	Health state	
I^d or S_3	Diagnosed	Recognized infections including the cases with or without symptoms, irrespective of how they were identified.	Simulation, state transition probabilities	Health (sub)state	
I^d_H or S_4	Hospitalized	Cases of moderate severity, hospitalization on the normal ward	Simulation, state transition probabilities	Health (sub)state	
I^d_{ICU} or S_5	In ICU	Agents that need treatment in an intensive care unit comprising the critically severe cases	Simulation, state transition probabilities	Health (sub)state	
R or S_6	Recovered	Agents have survived an (either asymptomatic or symptomatic) infection, are not infectious anymore, and are considered immune	Simulation, state transition probabilities	Health state	
D or S_7	Dead	Condition of the agents that have not recovered from the infection and died	Simulation, state transition probabilities	Health state	

	Regular schedules	Regular behavior of agents. A schedule covers 7 days (168 hours).	Age, occupation, health status	Model entity	
	Specific schedules	Schedules that determine agents (restricted) movements under defined circumstances. This comprises diagnosed, hospital schedule, death, non-pharmaceutical intervention	Health status, non-pharmaceutical interventions, locations, non-compliant probability	Model entity	
x_a	Interactivity	Probability for any agent a to interact with any other agent	Custom	Input parameter	
p^{inf}_{ab}	conditional probability for infection transmission	Infection transmission probability given an interaction occurred between an infected agent a and a susceptible agent b	Infectivity, infectivity distribution, time	Input parameter	
$I^I_a(t)$	Infectiousness	Infectiousness of a given infected agent a at time t	Time since infection, infectivity, infectivity distribution	Emergent parameter	
f^{loc}	Location-specific factor	Represents the infection risk at specific location type loc	Custom	Input parameter	
y^{inf}	Specific transmission coefficient	Specific coefficients for infected agent modulating the transmission probability between interacting agents	Custom	Input parameter	
y^s	Specific transmission coefficient	Specific coefficients for susceptible agent modulating the transmission probability between interacting agents	Custom	Input parameter	
P^{S}_{iT}	Remaining in state probability	Probability for being in state Si until time T	Probabilities to leave the state S_i before T	Emergent parameter	
P^t_{ij}	Remaining in state probability	Probability for staying in state S_i at time t			
N_i	age-resolved case numbers	number of cases in an age group		Input Data	RKI Factsheet
μ^t_{ij}	State transition mean time	The probability for each state transition X_{ij} is Gaussian distributed with mean at the reported or assumed average time t.		Input parameter	an der Heiden & Buchholz 2020
σ^t_{ij}	State transition standard deviation time	The standard deviation of Gaussian distribution equals its mean		Input parameter	
PMF	Probability mass function	Function that gives the probability that a discrete random variable is exactly equal to some value.		Mathematical function	
p^{ncomp}	Non-compliance probability	Reflects (sub)population fraction not complying with non-pharmaceutical interventions	Custom	Input parameter	
t^{trace}	Tracing time	Time frame in which retrospective contact tracing takes place	Custom	Input parameter	

Table S1: Symbols and abbreviations reference table. Description and dependencies for the different model entities and calculations of this document.

2 Model Design

To investigate the infection dynamics in a real-world community, e.g. Gangelt in Germany or Tepoztlan in Mexico, a simulation environment is required that reflects local peculiarities, like the amount and position of buildings and outdoor locations or size and demographic composition of the population. Furthermore, it is important to account for individual behavior of the members of the modeled population, like household interactions or (age-dependent) daily occupations. Finally, specifically in the context of an acute pandemic, the possibility to evaluate the effectiveness of (non-pharmaceutical) intervention strategies is required. Here, we provide a detailed documentation of the design and computational setup of GERDA, including a description of how we made use of external data and of the pipelines used to convert data into model input. Below, the design principles of GERDA are summarized, followed by sections about the parametrization and the computational analysis.

The GERDA model has been designed with three main goals: i) Simulation and analysis of transmission events, e.g. SARS-CoV-2 virus transmissions; ii) Applicability to different communities/Consideration of local peculiarities for simulations; iii) Creation of a test environment for the effect of (non-pharmaceutical) interventions. Those goals directly translate into three major design principles: first, the agent-based modeling approach, second, easily exchangeable external data input files, and third, a strict separation of a) model initialization, b) model parameterization, and c) model simulations. Those principles have been consistently applied throughout the entire development process.

The major object of GERDA is a so-called modeled world (MW) that provides the basic structure required to simulate virus propagation throughout the community. The MW is composed of locations (reflecting real-world buildings and outdoor spaces) and inhabited by agents (representing human individuals). To assure a realistic representation, locations and agents are initialized based on external input data and connected via households at model initialization. According to their schedules, agents visit locations at which they can interact with each other during a simulation. Upon interaction, an infection is potentially transmitted between individual agents. Infected agents can undergo various health state transitions reflecting disease progression until they either recover or die.

2.1 Locations

In the MW and throughout simulations, locations serve as containers for agents and, thereby, define the interaction topology in which agent-agent interactions and virus transmission take place. Hence, locations represent the spatial structure of the model. Every location is assigned to a predefined location type and has its spatial coordinates stored to allow distance-related distinction of locations for agents. Sets of locations in spatial proximity can be clustered into neighborhoods to further structure the MW. An outstanding feature of GERDA is the possibility to generate locations on the basis of geographical data (**Sec. 3.1**) to reflect the interaction and infection dynamics of real world communities. Alternatively, locations can be generated according to alternative principles or arbitrarily for comparison with a randomized case.

The assignment of locations into predefined location types allows agents to visit appropriate locations according to their specific daily or weekly routines. Furthermore, it provides the

possibility to adjust particular model parameters for a specific location type, for example when testing non-pharmaceutical intervention strategies applied only to schools.

2.1.1 Location Types

In the current version of GERDA, seven predefined location types are defined, i.e. workplaces, schools, public places, hospitals, morgue, homes and a mixing location, each with unique and common properties.

Workplaces are locations which are regularly visited by the agents assigned to the group of employees during their schedule-defined work hours. The work place of an agent is chosen with a likelihood inverse to the spatial distance to the home assigned to that agent. Pure workplaces represent, for example, office buildings, farms, or factories.

Public spaces can be visited throughout the day by all agents, which are not restricted otherwise (e.g. being in hospital). Additionally, they are assigned to a fraction of the agents representing employees as workplaces. The location type public space stands for a variety of different real world locations, such as parks, city halls, or restaurants.

Schools are daily visited by underage agents throughout the weekdays and are assigned to teachers as location of work. A specific location of type school is chosen for each underage agent according to the least distance to its assigned home location. In the current version of GERDA, schools comprise among others: kindergartens, elementary schools and high schools.

Hospitals are locations with two functions. On the one hand, they are assigned to medical personnel as workplaces and, on the other hand, they serve as containers for agents with severe or critical disease progression, i.e. being in the states I_H^d or I_{ICU}^d as explained below.

Morgue is the unique location foreseen for “deceased” agents. This location is required to prevent non-realistic interactions with agents of that status while keeping the agent number of the MW constant. At least one hospital and one morgue are required in any MW to assure functionality of the model, and they are automatically added if they are not present in the modeled community.

Home is the location type, which serves as a reference location for each modeled agent. Each agent is assigned to only one home, but one location of type home can serve as reference to more than one agent. Agents with the same home are referred to as one household.

The mixing location is an artificially introduced unique location. It is not required for real-world-related simulations, but is used to compare with simulations of a homogeneously mixed world.

2.2 Agents

The population of the MW consists of agents representing human individuals. The population can not be set independently but instead results from the number of created locations of type

home, i.e. the households. Based on the number of households and the demographic input data (described 3.2), the model generates a population of agents which represents realistic distributions of ages and household sizes.

2.2.1 Agent Properties

Through the household initialization process, each agent obtains an age in years and an age-related occupation. Occupations resemble child/student for younger agents, employees in middle ages, and pensioners for older agents (more details below). Each agent is uniquely identifiable by an ID.

Agents have two major properties that define their potential operations: a weekly schedule and a health state. The schedule specifies the agent's time-dependent presence at locations and, thereby, its potential to interact with other agents and become infected. The health state can change depending on interactions with infected individuals or during the disease progression. It modifies the potential operations of the agent leading to altered schedules. GERDA includes four main health states, namely susceptible (S), infected (I), recovered (R), and dead (D) with the default health state S (**Fig. S1**).

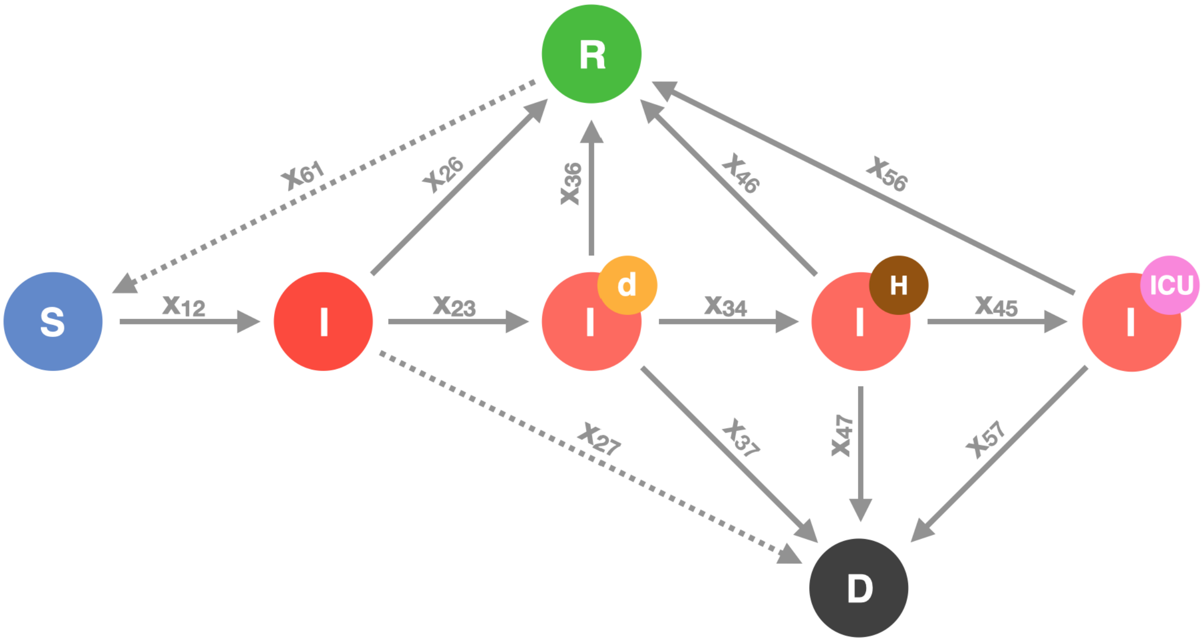

Figure S1: **Health states and potential transitions.** Agents can be susceptible (S), infected (I), recovered (R), or dead (D). The group of infected agents comprises the presymptomatic or asymptomatic infected (plain I), the diagnosed infected (I^d), the hospitalized infected ($I^d{}_H$) and the infected requiring intensive care unit ($I^d{}_{ICU}$). The transitions indicated by solid arrows are implemented, the transitions indicated by dotted arrows are foreseen, but not used in the current simulations (probabilities set to zero).

Below, we describe first the schedules and the resulting potential interactions between agents. Then, we introduce the infection transmission process, which links the concept of schedules and locations with the concept of health states. Eventually, we explain the considered health states and transitions between them in detail.

2.2.2 Agent Operations - Schedules and Occupation

Every agent has a weekly schedule, which is assigned based on age and a given schedule distribution for the corresponding age group during agent initialization. This schedule determines the locations visited by the agent over the period of one week in an hourly resolution. Different agent subgroups share a certain schedule type, i.e. their schedules comprise the same location types, but differ in the exact location and the exact times of movement. These subgroups are underage, working adult, teacher, medical professional, public worker and pensioner and reflect the occupation of an agent. Each subgroup consists of a fraction of people within a specific age section (0-17: underage; 18-66: working adults, teachers, medical professionals, public workers; 67+: pensioners). The schedules are designed in a way that every agent spends some time at its home (typically the nights). During the day, underaged visit schools and (working) adults visit workplaces. Teachers, medical professionals or public workers, visit schools, hospitals, and public places in a comparable regular fashion, respectively. Public workers have schedules similar to other workers except their working place is a defined public place and their work hours can also be at the weekend. 20 % of the working population are assigned to public places. All agents occasionally visit public places during weekdays and more extensively on weekends.

In addition to the regular schedule, all agents share the so-called specific schedules that determine their (restricted) movements under defined circumstances, in the model usually represented by defined health (sub-)states, such as a diagnosed schedule, a hospital schedule, and a death schedule (see **Fig. S3**). These schedules constantly assign the corresponding agent to a single location, i.e. either the agent's home, a hospital or the morgue, as long as the agent's state is I^d (diagnosed), I^d_H (hospitalized), I^d_{ICU} (in ICU) or D (dead), respectively. The only exception to this are non-compliant agents, who refuse to stay at home when they are diagnosed (I^d), but keep on following their regular schedule instead of a diagnosed schedule. Examples for schedules are given in **subsection 3.3.2**.

During a simulation, one schedule, by default the regular schedule, is set to active. The agents follow their active weekly schedules in the exact same way every week. The active schedule is changed to a specific schedule when an agent changes its health state. When non-pharmaceutical measures (e.g. lockdown) are requested (for all agents or a subgroup of agents), the schedules get modified accordingly for the duration of the implemented measures, i.e. such that agents stay in their respective homes, instead of visiting specified location types.

The schedules are the basis of the agents' interaction network. It is important to note that while agents visit the same locations at the same time each week, they possibly interact with different other agents on each visit. The interaction network is the basis for potential infection transmission resulting in the agent's health state transitions over time. As stated above, health state transitions can feed back into schedule activation (principles illustrated in **Fig. S2**).

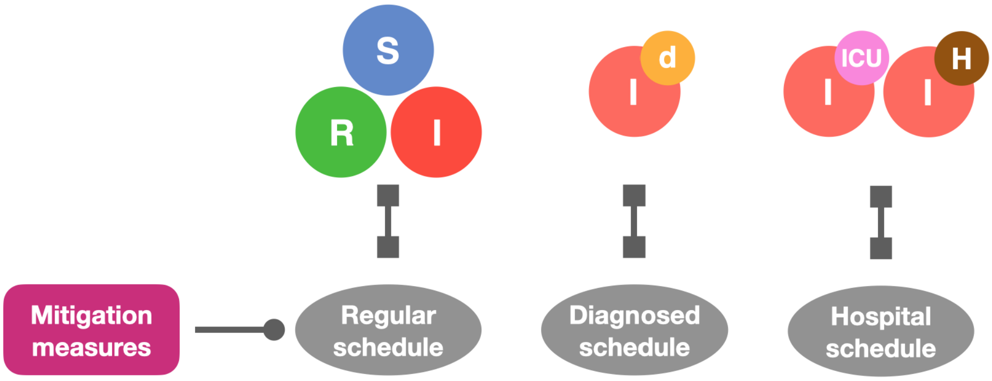

Figure S2 - Schedule assignment and modification. Agents have an active schedule at any time. By default, this is the regular schedule, which can be changed upon health state transition or application of non-pharmaceutical interventions. While regular schedules differ between agents, diagnosed, hospitalized, and dead schedules are specific schedules that place an agent 24/7 at home, in the hospital, or on the morgue, respectively. For non-compliant agents, the mitigation or lockdown schedules can be set equal to their regular schedule.

2.2.3 Motion Between Locations According to Schedules

During a simulation, each agent sequentially visits different locations, defined by its schedule. The resolution for location changes is per hour, in consistency with [37] which showed that 70% of the social contacts last an hour or more . Several agents can visit the same location at the same time (**Fig. S3**). This spatio-temporal overlap of agents creates their network of possible interactions and it further facilitates the network for infection spreading.

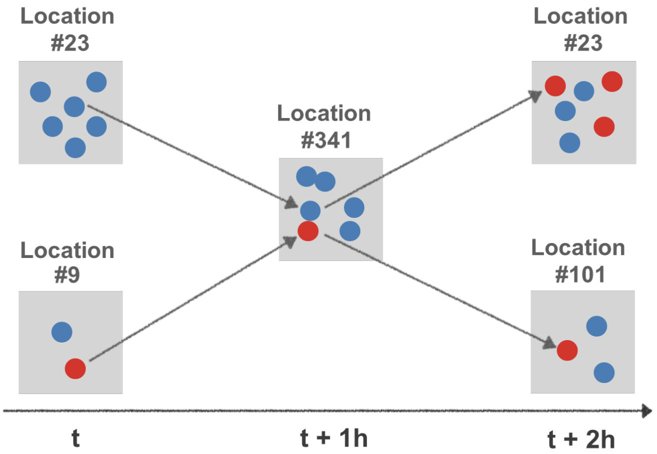

Figure S3: Schematic representation of agents visiting locations at different times *t*. At time *t*, six susceptible agents are at location #23 and one susceptible and one infected at location #9. In the next time step (*t+1*), one susceptible agent from location#23 and the infected agent from location #9 move to location #341. Here, they interact and transmit the infection. At time *t*+2, the newly infected agent moves back to location #23, while the first infected agent continues to location #101. Two other infected agents have moved to location #23 between times *t* and *t*+2.

2.2.4 Agent-agent Interactions

During a simulation, all agents with spatio-temporal overlap, i.e. being at the same location at the same time step, have the possibility to interact with each other. Interacting pairs are determined, regardless of their health state, by a stochastic process. Each of the n_{loc} agents in a location loc at time t has the possibility to interact with each of the n_{loc}-1 other agents with a given probability. We define a global interaction frequency μ. Due to the stochastic nature of the interaction mechanism, some agents have more than the expected number of interactions while others do not have any.

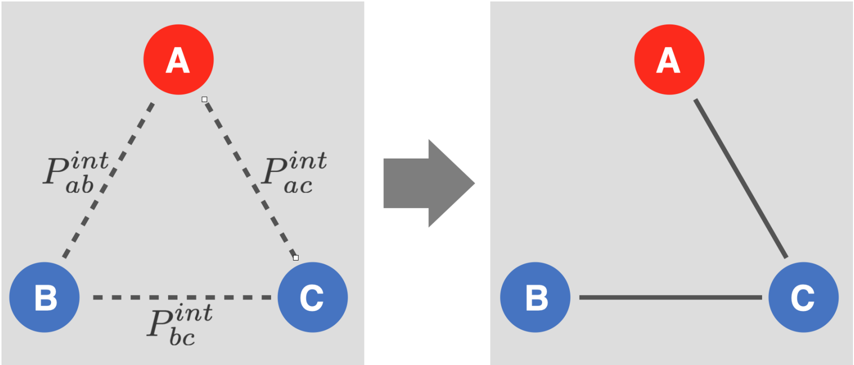

Figure S4: Schematic representation of agent-agent interactions. Left: Each agent has a certain probability to interact with each other agent having spatio-temporal overlap. Right: realized interactions.

The probability for any agent a to interact with any other agent b in the same location is dependent on their specific interactivities x_a and x_b, the global interaction parameter μ and the number of agents per location $n_{loc} = n_{loc}(t)$ (where $n_{loc} \geq 2$ in order to enable an interaction).

Equation (1) defines the interaction probability P_{ab}^{int} of two agents a and b per time step (at specific location):

$$P_{ab}^{int} = \frac{Minimum(n_{loc} - 1, x_a x_b \mu)}{n_{loc} - 1}$$

Equation (1)

This interaction probability is calculated for all potential pairs of agents present at the same time at the same location. The interaction is realized if a random number between 0 and 1 is smaller than the interaction probability.

Specific interactivities of agents, x_a, are set to 1 as default value to reflect airborne infection transmission, but can be set differently to reflect specific individual behavior. The influence of varying interactivities is shown in 4.10, including interativities sampled from log-normal or power-law distributions.

The global interaction frequency μ is the expected number of interactions per time step for sufficiently occupied locations and $x_a = x_b = 1$. The realized number of interactions depends on the number of agents currently present at the specified location. Since an agent can maximally interact with n_{loc}- 1 other agents at any given time, the real mean of interactions may be lower than μ, if n_{loc}- 1 is smaller than μ.

2.2.5 Infection Transmission

$P_{ab}^{inf}(t, loc)$is the probability for the infection transmission from agent a to agent b at location loc, where t denotes the infection duration of agent a in hours.

Given an interaction occurred between an infected agent a and a susceptible agent b, the conditional probability for infection transmission is defined as:

$$p_{ab}^{inf} = p_{ab}^{inf}(t, loc) = Minimum\big(1, k_I \cdot I_a^I(t)\big)$$

Equation (2)

with $I_a^I(t)$ being the infectiousness of the infected agent and k_I a globally fitted infection parameter. The approach can be extended such that parameter $k_I$is

$$k_I = k_I\big(y_a^{inf}, y_b^{inf}, f^{loc}\big)$$

Equation (3)

where f^{loc} is a location-specific factor representing the infection risk at different types of locations (offices, hospital, outside areas) and y^{inf} presents specific coefficients for the interacting agents modulating the transmission probability (e.g. representing protective gear). k_I can be also varied to represent different virus strains.

Hence, the probability for the infection to get transmitted from agent a to agent b at location loc and at time t is the product of the conditional probability for the infection transmission and the probability for an interaction to occur,

$$P_{ab}^{inf} = p_{ab}^{inf} \cdot P_{ab}^{int}.$$

Equation (4)

Values for the infection-duration dependent infectiousness $I_a^I(t)$ are given in **section 3.5** and **Table S26 (E)**.

2.2.6 Health States

As mentioned above, agents can assume different health states, namely susceptible (S), infected (I), recovered (R), and dead (D) with the default health state S. In the previous section, we described the infection transmission, i.e. the transition from state susceptible to state

infected (see **Fig. S1**). Here, we provide more details about the infected substates and about transitions between all health states.

The four major health states (S,I,R,D) specify the epidemiological conditions of the agents. They are the basis for simulating virus infection progression in the modeled population and to analyze transmission chains on individual level. In order to cover disease progression for individual agents more precisely, the infected state is divided into four sub-states that can be associated with severity of progression and are described in detail below. In summary, this gives us seven distinguishable health states (see **Fig. S1**), of which the four representing different infected states are transient, i.e., no agent can remain infected infinitely. Agent behavior is influenced by the health state: Being susceptible or infected without diagnosis, an agent follows its regular schedule while upon infection diagnosis the diagnosis schedule gets activated.

Susceptible agents have never been subject to the infection and neither are immune to it. They can become infected based on an interaction with an infected agent. They follow their regular schedule.

Recovered agents have survived an (either asymptomatic or symptomatic) infection, are not infectious anymore, and are considered immune. The model allows for loss of immunity by a transition from state R to state S, in order to account for the latest discussions about potential SARS-Cov2 antibody loss. However, this transition is disabled in the presented study (by parameterization with zero), due to lack of sufficient evidence.

Infected agents comprise presymptomatic, asymptomatic, and symptomatic infected individuals. In order to specify and distinguish between different types of severity of the infection, various attributes are associated with the different substates of the infection. Those attributes affect agent behavior, e.g. by activating the diagnosed schedule, and ultimately reflect the information available during a real-world pandemic. The plain infected state (I) represents the unrecognized cases, i.e. the vast majority of presymptomatic or asymptomatic cases. The diagnosed state (I^d) represents the recognized infections including cases with or without symptoms, irrespective of how they were identified. These diagnosed cases may escalate towards two subsequent stages of severity. These are either hospitalization on the normal ward for cases of moderate severity (I^d_H) or the treatment in an intensive care unit (ICU) for the critically severe cases (I^d_{ICU}).

Due to parameterization (as explained in **section 3.4**), all infected states are transient.

Infected individuals have a probability to either recover or to die. Birth and death processes of the susceptible or recovered states are neglected in the current version of the model.

Dead agents are assigned to the morgue.

Throughout the manuscript and simulations, we use the following color code: susceptible – blue, recovered – green, infected – red, dead – black. In addition, for the subgroups of infected holds: diagnosed – orange, hospitalized – brown, in ICU – magenta (**Fig. S1**).

Formally, we will refer to the states S_i, $i = 1,..,7$. The assignment of states to numbers is given in **Table S2**.

**State**	S	I	I^d	I^d_H	I^d_{ICU}	R	D
**Symbol**	S_1	S_2	S_3	S_4	S_5	S_6	S_7

Table S2: Numbering of health states for formal analyses.

2.2.7 Health State Transitions

At each time step, an infected agent can either remain in its current state, progress to another infected state, recover, or die (**Fig. S1** and **Table S3**). This is determined by a discrete stochastic decision process, based on the respective probabilities given in Tables **S26 (A-E)**, which is detailed in section **3.4**.

	S_1	S_2	S_3	S_4	S_5	S_6	S_7
S_1	X_{11}	X_{12}	0	0	0	0	0
S_2	0	X_{22}	X_{23}	0	0	X_{26}	X_{27}
S_3	0	0	X_{33}	X_{34}	0	X_{36}	X_{37}
S_4	0	0	0	X_{44}	X_{45}	X_{46}	X_{47}
S_5	0	0	0	0	X_{55}	X_{56}	X_{57}
S_6	X_{61}	0	0	0	0	X_{66}	0
S_7	0	0	0	0	0	0	X_{77}

Table S3: Possible Transitions X_{ij} in agreement with the network shown in **Fig. S1**.

Formally, we will refer to the allowed state transitions by X_{ij} with $i, j = 1, .., 7,$ as shown in **Table S3**. In order to determine whether a health state transition occurs, we used the following probabilities:

P^{S}_{iT} is the probability for being in state S_i at time T.

P^{c}_{ij} is the cumulative probability for transition from S_i to S_j (total probability).

P^{X}_{ijt} is the probability for transition from S_i to S_j (per time step).

p^{X}_{ijt} is the conditional probability for transition from S_i to S_j (per time step).

Together with the transition from susceptible to infected, we consider in total 13 potential transitions in the model, as listed below together with the respective probabilities:

State Transition	Symbol	transition probability
S → I	X_{12}	p^{X}_{12t}
I → I^d	X_{23}	p^{X}_{23t}
I → R	X_{26}	p^{X}_{26t}
I → D	X_{27}	p^{X}_{27t}
I^d → I^d_H	X_{34}	p^{X}_{34t}
I^d → R	X_{36}	p^{X}_{36t}
I^d → D	X_{37}	p^{X}_{37t}

$I^d_H \rightarrow I^d_{ICU}$	X_{45}	p^X_{45t}
$I^d_H \rightarrow R$	X_{46}	p^X_{46t}
$I^d_H \rightarrow D$	X_{47}	p^X_{47t}
$I^d_{ICU} \rightarrow R$	X_{56}	p^X_{56t}
$I^d_{ICU} \rightarrow D$	X_{57}	p^X_{57t}
$R \rightarrow S$	X_{61}	p^X_{61t}

Table S4: Health state transitions and the respective cumulative transition probabilities.

Considering all possible state transitions $X_{ij}$to change from state S_i to S_j,the probability for each transition then is given by:

$$P^X_{ijT} = P\,(X_{ij}, T) = P(X_{ij}, T \mid X_{ii}, t = 1, \ldots, T-1) \cdot P(X_{ii}, t = 1, \ldots, T-1),$$

Equation (5)

where the probability (P^S_{iT}) to remain in the state S_i until time *T* is one minus the sum of the probabilities to leave the state before *T*:

$$P^S_{iT} = P(X_{ii}, t = 1, \ldots, T-1) = 1 - \sum_{t=1}^{T-1} \sum_{j=1}^{n_S} P^X_{ijt}.$$

Equation (6)

We now can rewrite the Equation (5) for the state transition at time T to:

$$P^X_{ijT} = p^X_{ijT} \cdot P^S_{iT}.$$

Equation (7)

Hence the required conditional probability for a state transition at each time step used in the GERDA-model follows as .

$$p_{ijT}^{X} = \frac{P_{ijT}^{X}}{P_{iT}^{S}} = \frac{P_{ijT}^{X}}{1-\sum_{t=1}^{T-1} \sum_{j=1}^{n_S} P_{ijt}^{X}},$$

Equation (8)

which is the required conditional probability for a state transition at each time step used in the GERDA-model.

The derivation of numerical values for the transition probabilities from published data is detailed in Sec 3.4.2.

3 Model Initialization and Data Integration

The model is initialized in three steps:

(i) Locations are initialized either from real geographical data (default) or arbitrarily (for alternative scenarios or as randomized comparison). The location type home defines the number of households.

(ii) Households are initialized based on country-specific zensus data assigning household types to all home locations (1:1 ratio) and defining a respective number of household members (1:n ratio). Households are only relevant for initialization; they are not stored as simulation objects.

(iii) Agents are initialized according to households and with a number of attributes that either depend on their household membership or on additional data. Agents obtain an occupation and a weekly schedule that is related to their occupation.

Underlying data and details are explained below. Details of code are described in GitLab.

3.1 Location Initialization

3.1.1 Principles of Location Initialization

The initial step in GERDA is the generation of an *in silico* representation of the real-world community of interest through the creation of distinct locations. These locations define the space in which the agents can interact, and, in addition, the size of the agent population as described in **Sec. 2.2** and further explained below in **Sec. 3.2**.

In general, locations are initialized from an input data file (see **Sec. 3.1.2**) specifying the floor area, the geographical coordinates and, if appropriate, the neighborhood identifier of that location. The input data contains further annotations of each building in one of the following annotation categories: building, amenity, shop, leisure, sport or healthcare. According to these annotations, each building is assigned a specific location type as introduced in **Sec. 2.1**. For the municipalities Heinsberg and Gangelt an assignment table (**Table S5**) was created which ought to be extended, when including further geolocations.

location type	resp. specific annotations	annotation category
workplace	industria, greenhouse, cowshed, shed, commercial, warehouse, office, farm, fire station, farm auxiliary, retail	
public place	public, chapel, church, parish hall, townhall, restaurant, grocery store, cafe, sports center	leisure, sport
school	school, university, kindergarten	
hospital		healthcare

morgue	-	-
home	residential building	
excluded building	garage, roof, shed, bungalow, silo, barn	

Table S5. Location type assignment. All initialized buildings are assigned one location type, if applicable, according to the distinct annotation category and, otherwise, according to a specific annotation.

If a building is not annotated otherwise, its location type is set to home during initialization. Further if any of the two required locations, morgue or hospital, are not assigned to at least one location, they are added as an initialized location at the border of the generated world. Alternatively to the described procedure, one location called mixing location is initialized to allow for comparison with non-localized agent based models.

3.1.2 Data used for Location Initialization

To generate the geospatial maps, we utilized OpenStreetMap (OSM), an editable map database built and maintained by volunteers, distributed under the Open Data Commons Open Database License, and available from https://www.openstreetmap.org. OSM data retrieval is facilitated by the OSMnx package for python [38] and was integrated generically. Using the OSM addresses of sufficiently annotated administrative regions, such as the community Gangelt, data on area, street network, and buildings is downloaded. Area and street network data is used for visualization purposes only. All retrieved buildings of the community of interest, whose floor area is above a predefined threshold, are considered as locations in the model. Thresholding is necessary since not all buildings in the OSM database are sufficiently large to be considered a location with respect to our model categories. The building data is cropped to those relevant to determine the building functionality and extended by a manually curated neighborhood identifier to subdivide locations in functional clusters.

Whenever OSM data collection is incomplete, as is the case for Tepoztlán, we use data provided by government agencies (https://www.inegi.org.mx/app/mapas/default.html?t=0710000000000000&ag=01). Relevant files and specific references are given in the GitLab repository (https://tbp-klipp.science/GERDA-model/). In short, the government vectorial shape files were transformed to csv files where the columns were re-arranged to show the same architecture as the files generated when OSM was used.

3.2 Household Initialization

3.2.1 Principles of Household/Population Initialization

To create a realistic population, the agents of a MW are initialized based on the concept of households. By iterating over all locations of type home and assigning them a household type and size from a country-specific data-based distribution, a respective number of agents is initialized. Each agent belongs to a unique household. This way, we create a reasonable demographic as well as a family structure of the agent population (**Fig. S5**). Several agent properties depend directly on the agent's household, while further properties are added consecutively. The data that was used for agent initialization and the dependencies of agent properties on that data and on each other are detailed in the following subsections.

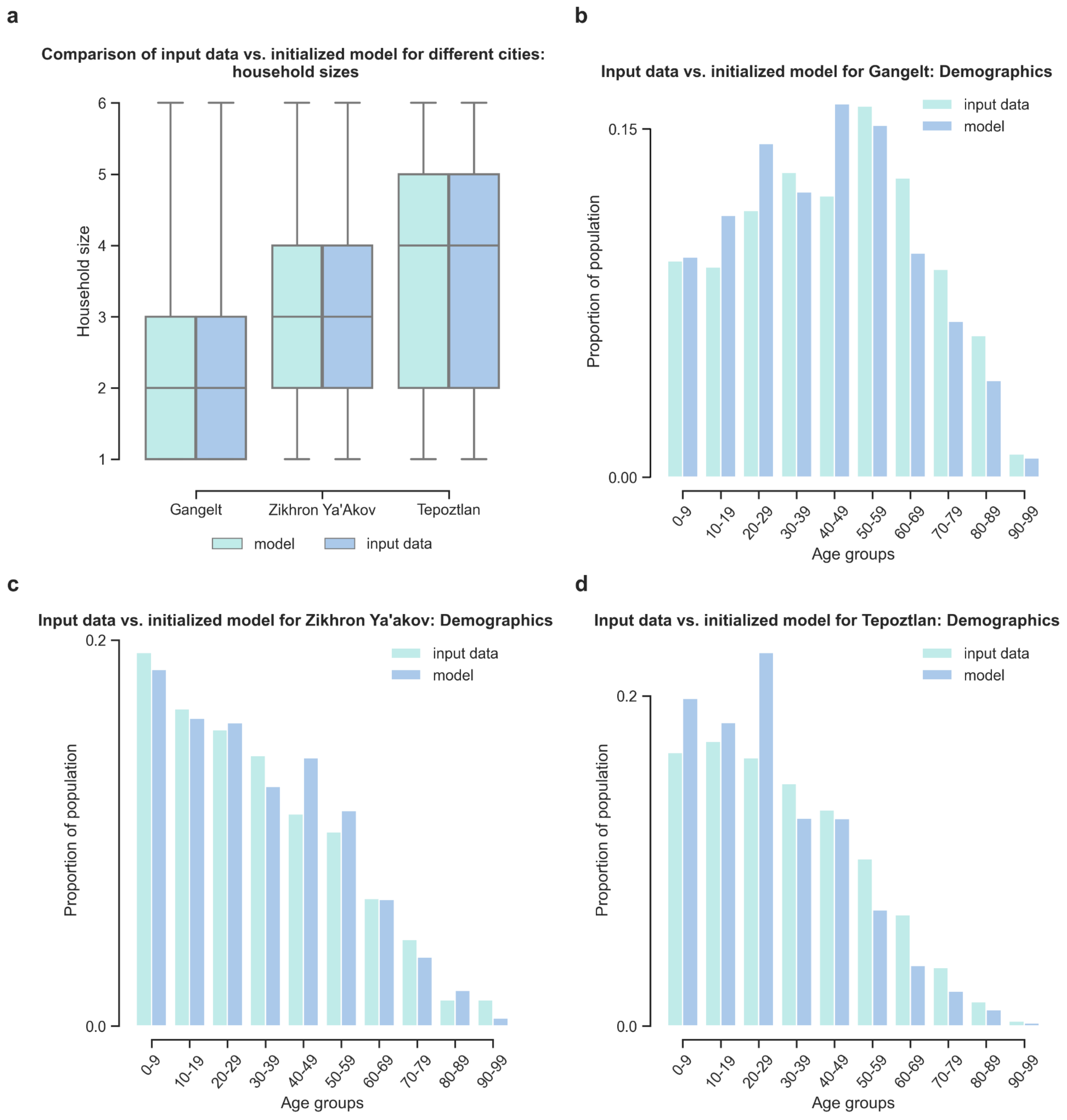

Figure S5: Comparison of input data and initialized model. a Box plot comparing household sizes for Gangelt (Germany), Zikhron Ya'akov (Israel), and Tepoztlán (Mexico) with their model counterparts. **b-d** Age pyramids of Germany, Israel, and Mexico (input data) compared to the respective initialized agent populations.

3.2.2 Data for Household Initialization

For the definition of households, we used data from three different countries, i.e. Germany, Mexico, and Israel.

For Germany, we obtained the relevant data from the German Zensus 2011 database that is available online (https://ergebnisse.zensus2011.de). The data can be obtained either in form of predefined tables (https://ergebnisse.zensus2011.de/#StaticContent:00,,,) or by specifying the combination of desired information in the dynamic table section (https://ergebnisse.zensus2011.de/#dynTable:). If not specified otherwise, the tables in this subsection were predefined. To use the latest age distribution, the age distribution for the German population of 2020 was integrated from (https://service.destatis.de/bevoelkerungspyramide). All respective data has been released by the German government and details on data collection and processing are described at the given webpage for each table. In the following subsections we show exemplarily for Germany how we converted the public census data into the input files required for GERDA simulation. Accordingly, we include Supplementary Tables for the used data and the input files for simulating German communities. For the other countries, the respective input files can be found in our public GitLab repository.

For Israel, we used data from the Population Census 2008, provided by the Israeli Bureau of Statistics (https://www.cbs.gov.il/en/subjects/Pages/The-2008-Census-of-Population.aspx). To produce the equivalent of the GERDA input files described in Supplementary Table S10 to S12, we used the following Census Tables: i) 'Table 6.3 - Households, by type of household and district' (for equivalent of Table S10), ii) 'Table 6.1 - Households, by type of household and age group of reference person' and 'Table 6.4 - Households, by household size and age group of the reference person' (for equivalent of Table S11), iii) 'Table 6.7 - Population in Households, by type of household and age group', and 'Table 1.21 - Population and households in localities with 5,000 residents and over' (for equivalent of Table S12).

For Mexico, we obtained data from the intercensal survey 2015 from the National Institute for Statistics and Geography (INEGI) (https://www.inegi.org.mx/programas/intercensal/2015/#Tabulados). The data is obtained as predefined tables for the whole country or state specific. Whenever possible, we used information for the whole country rather than for specific locations. In order to match the GERDA defined input tables, house types were homologated to our five archetypal house types (compare Supplementary Table S10). To use the latest age distribution, the age distribution for the Mexican population of 2020 was obtained from https://www.inegi.org.mx/programas/intercensal/2015/#Tabulados (section hogares). Original data and further specifications of which tables were used are given in the GitLab repository (https://tbp-klipp.science/GERDA-model/).

3.2.2.1 Conversion of public data into GERDA input tables

Tables S6 to S12 listed below are available in our separate **Supplementary Tables** file.

- **Table S6: Household type frequencies.** Absolute and relative frequencies of household/family types in Germany. The table content has been translated to English, original German content is kept in brackets and small font to allow comparison with the original source.

  https://ergebnisse.zensus2011.de/#StaticContent:00,BEG_3_1,m,table

- **Table S7: Absolute distribution of households according to type and size.** Original information obtained from the German Federal Government by using the dynamic table section of the Zensus 2011 database. The table content has been translated to English, original German content is kept in brackets and small font to allow comparison with the original source.

  https://ergebnisse.zensus2011.de/#dynTable:statUnit=HAUSHALT;absRel=ANZAHL;ags=00;agsAxis=X;xAxis=HHGROESS_KLASS;yAxis=HHTYP_FAM

- **Table S8: Age distributions per household type.** Total number of people in each age range (11 groups) per household type. The table content has been translated to English, original German content is kept in brackets and small font to allow comparison with the original source. As there was no specific information provided concerning the underage members of couples without kids and they only compose a very minor fraction of the population (<0.1%), we omitted the numbers grayed out.

  https://ergebnisse.zensus2011.de/#StaticContent:00,BEV_10_25,m,table

- **Table S9: Current age distribution in Germany.** This table was extracted from the 'Bevölkerungspyramide' (i.e. 'age pyramide') and shows the complete age distribution of the German population for 2020.

  https://service.destatis.de/bevoelkerungspyramide/#!y=2020

According to our concept to initialize the modeled population based on available home locations, the household data used is based on occupied flats/houses and not on families. The GERDA model in its current version allows five household types: Single person household, couple without kids, couple with kid(s), single parent household, and multi-person household without nuclear family. Household sizes range from one to six agents, with bigger households being included in size category '6+' in the data. For sake of simplicity, a few assumptions have been made when integrating the data. Household types that compose a minor fraction of the population have been included within the five types introduced above. E.g. couples without kids can only have a household size of two, thereby omitting specific households like grandparents with children. The omitted special cases as well as multi-generation households are included in the household category multi-person households without nuclear family. An algorithm was created to combine the above described data into probability distributions from which the number of agents per household and ages of the resulting household members are drawn. It is outlined below. The distributions used for GERDA simulation are listed below:

- **Table S10: Relative household type distribution.** Calculated from Table S6.
- **Table S11: Relative household size distribution.** Household-type-dependent; calculated from Table S7. For simplicity in implementing household initialization, the household type ‘couples without kids’ can only be of size 2, thereby ignoring very specific family types, such as grandparents with a kid. Those ignored family types compose about 1.6% of the total population. Used as input for GERDA simulation.
- **Table S12: Relative age distribution per household type.** Calculated from Tables S8 and S9.

3.2.2.2 Algorithm

For household initialization, the probabilistic relative distributions obtained from the tables introduced above are used in the following order: First, the household type is sampled (**Table S10**). Second, the household size is sampled in a household-type dependent manner (**Table S11**). Finally, the age(s) of the agents living in the household (number of agents = household size) are sampled. This last step is achieved using a probabilistic distribution based on mapping the real distribution of ages in Germany 2020 (**Table S9**) as weights to the household type age distribution (**Table S8**). This results in the calculation of the relative age-dependent frequencies for each household-type (0-99 years) (**Table S12**). The average age difference between couples, between parents and their children, and between siblings are provided as model input parameters in a country-specific .yaml file.

As a result, agents are initialized with a unique ID, a home location, and an age. Age assignment of all agents of a household is accomplished in a household type-dependent manner: i) If the household contains kids (couple with kids or single parent household), the algorithm starts sampling the kid(s) (number of kids = n-2 or n-1, respectively), and then the parent(s) age(s) are sampled from a shifted normal distribution depending on the age of the eldest kid. The mean of the age-offset for the eldest kid and its parents depends on the demographics and was set to 30 years for Germany, 21 years for Mexico, and 27 years for Israel. In addition, the maximum age of a child in a household is set to 30 years. The sampling of sibling(s) is made by a shifted normal distribution, around an average demographic dependent age difference (3.5 years for Germany, 2 years for Israel, and 4.3 years for Mexico) and a defined standard deviation (1.75 years for Germany, 1 year for Israel, and 2.7 years for Mexico). To avoid negative ages, absolute values are taken. ii) In case of a couple without kids, the first member is sampled and the generated age of this member is used to make a normal distribution with a standard deviation of 1/10 of this age. The age of the second member is obtained based on the aforementioned normal distribution. iii) In the rest of the cases the age is obtained by the corresponding probability distribution of ages per household type.

The output of the algorithm provides a household with type, size, and ages of the respective number of household members for each home location.

3.3 Agent Initialization

3.3.1 Principles of Agent Initialization

For each member of a household, an agent is initialized with a unique ID, the household's location as home location, a household-dependent age, and, by default, the health state susceptible. To evaluate the correct reproduction of our input data, we compared the age pyramids of Germany, Israel, and Mexico to the age distributions of modeled worlds for Gangelt (Germany), Zikhron Ya'akov (Israel), and Tepoztlán (Mexico) (**Fig. S5 b-d**). Based on its age a schedule is created for this agent that also defines the occupation. This schedule determines the agent's movement during a simulation. The details about schedule creation during model initialization are described in the following subsection.

The subsequent section outlines the optional possibility to set an agent's health state to *infected* upon initialization.

3.3.2 Schedule Generation for Agents

Schedules are created individually for each agent during agent initialization. Age and home location of an agent are required to generate its regular schedule from a user-adjustable input table of possible schedule types. Conceptually, there is no limit to the number of possible schedules and detailed instructions on how to set up a list of schedule types are given in the GitLab repository (https://tbp-klipp.science/GERDA-model/). The predefined schedule types are specific for different age categories, such that typical occupations for people of different ages can be accounted for. Within each age category, a set of possible schedule types and their proportions are defined. The proportion reflects the fraction of people in the corresponding age category that should follow the respective schedule type. This type does not have to be exclusive, so there can be many schedules of type 'medical professional', for example, reflecting agents with the same occupation, but different shifts or free time habits.

For each agent, the regular schedule is drawn from the set of schedule types within its age category. Thereby, the above mentioned proportions serve as draw probabilities. By definition, a schedule covers 7 days (168 hours), to account for differences in agent movements and localizations between different days, e.g. during work days and weekends. The schedules contain a list of times (hours of the week) or time spans with a resolution of one hour and a list of location types. The times determine the hours of the week at which the agent moves from its current location to the next. If a time span is provided, the exact time for the movement is drawn from a uniform distribution within the time span. When the exact times are defined, the agent gets assigned a set of explicit locations. For each location type in the schedule an explicit location of the corresponding type is drawn with a probability linearly decreasing with their relative distance to the agent's home location. Relative distance means that all existing locations of this type are ranked by their distance to the home location, such that only the order of distances and not the real geographical distance is of importance. If there are several locations of the same type in a schedule, they are denoted with a number (e.g. public 1, public 2). For each number, a different location of this type is drawn that stays associated with this

number for this agent. For example, public 1 is always the same location of type public whenever it appears in the agent's schedule. Once all exact times and locations are set, they define the regular schedule for this agent.

During the simulation, an agent can get assigned a health-state dependent schedule, i.e., a diagnosed or a hospitalized schedule, respectively, for a limited time. The diagnosed schedule reflects quarantine rules, i.e. the agent has to stay at home. With a hospitalized schedule, the agent stays in the hospital. In case of non-compliance, the agent continues to follow its regular schedule.

Lockdown measures or NPI were implemented such that if agents are excluded from locations of a specific type, e.g. public places, they are relocated to their home location instead.

**schedule type**	**pensioner**		**working adult**		**underage**		**diagnosed**	
	**time, h (week)**	**location**	**time**	**location**	**time**	**location**	**time**	**location**
	0	home	0	home	0	home	0	home
	10	public 1	8	work	7	school	9	home
	11	home	16	public 1	15	public 1	14	home
	36	public 2	18	home	18	home	19	home
	37	home	32	work	32	school	33	home
	55	public 3	40	public 2	39	public 2	38	home
	56	home	42	home	44	home	43	home
	82	public 1	56	work	56	school	57	home
	85	home	64	public 3	62	public 3	62	home
	112	public 3	65	home	68	home	67	home
	113	home	80	work	80	school	81	home
	127	public 2	88	public 1	85	public 1	86	home
	128	home	89	home	93	home	91	home
	156	public 4	104	work	104	school	105	home
	157	home	112	public 3	110	public 3	110	home
			114	home	115	home	115	home
			133	public 4	132	public 4	129	home
			138	home	138	home	134	home
			154	public 5	155	public 5	139	home
			163	home	162	home	153	home

158	home
163	home

Table S13. Example schedules. Agent-specific schedules with fixed hours, possibly ranging from 0 to 167 hours (over the course of a week) defining the agents' movements during a simulation.

Each agent has an active schedule at each time that specifies its movements between locations over time. **Table S13** shows three typical regular schedules of different types and one diagnosed schedule. **Fig. S6** shows a snapshot of the complete input table for schedules to be drawn from, which is available in the additional file for **Supplementary Tables, Table S14**. For detailed information about creation of schedules, please refer to https://tbp-klipp.science/GERDA-model/schedule.

age (upper bound)	version	fraction	description	Start time	expand to range	**mo**	Start time	expand to range	**tu**	Start time	expand to range	**we**	Start time	expand to range	**th**
18	1	1	underage	7	9	school	7	9	school	7	9	school	7	9	school
				13	16	public_1	13	16	public_2	13	16	public_3	13	16	public_1
				17	22	home	17	22	home	17	22	home	17	22	home
67	1	0.77	adult	7	12	work	mo		work	mo		work	mo		work
				p7	9	public_1	mo		public_2	mo		public_3	mo		public_1
				p1	3	home	p1	3	home	p1	3	home	p1	3	home
	2	0.004	medical_professional	7	12	hospital	mo		hospital	mo		hospital	mo		hospital
				p7	9	public_1	mo		public_2	mo		public_3	mo		public_1
				p1	3	home	p1	3	home	p1	3	home	p1	3	home
	3	0.003	medical_professional	10	16	public_4	10	16	public_5	7	12	hospital	we		hospital
				17	22	home	17	22	home	p7	9	public_3	we		public_1
										p1	3	home	p1	3	home
	4	0.0015	medical_professional	6	7	home	mo		home	mo		home	mo		home
				p12	14	public_1	p12	14	public_2	p12	14	public_3	p12	14	public_1
				22	23	hospital	mo		hospital	mo		hospital	mo		hospital
	5	0.0015	medical_professional	6	7	home	10	16	public_5	mo		home	mo		home
				p12	14	public_4	17	22	home	p12	14	public_1	p12	14	public_2
				p2	3	home				22	23	hospital	we		hospital
	6	0.02	teacher	7	9	school	7	9	school	7	9	school	7	9	school
				13	15	public_1	13	15	public_2	13	15	public_3	13	15	public_1
				16	18	home	16	18	home	16	18	home	16	18	home
	7	0.1	public_worker	7	9	public_1	mo		public_1	mo		public_1	mo		public_1
				p6	8	public_2	mo		public_3	mo		public_4	mo		public_2
				p1	3	home	p1	3	home	p1	3	home	p1	3	home
	8	0.1	public_worker	11	13	public_2	mo		public_3	mo		public_2	mo		public_4
				p1	3	public_1	mo		public_1	mo		public_1	mo		public_1
				p6	8	home	p1	3	home	p1	3	home	p1	3	home

Figure S6: Snapshot of the schedule input table. The possible schedules are defined by an input table that determines age categories and different schedule versions within each category that reflect typical schedules for different occupations. The corresponding complete **Table S14** is found in the additional Supplementary Tables file, detailed instructions on schedule creation are found at https://tbp-klipp.science/GERDA-model/schedule.

As outlined above, the generated schedules are agent specific, depending on the individual archetype. But they also differ between individuals of the same archetype with respect to moving times and exact times they spend in different locations. Some underage agents, for example, spend more time at public places than others on a certain weekday and less on another, or visit public places at different times than others but spend equal amounts of hours there. This holds for every archetype and every type of location/activity. Resulting distributions of time spent by different archetypes at different locations or time spent at different locations resolved by archetype are shown in **Fig. S7 and Fig. S8**.

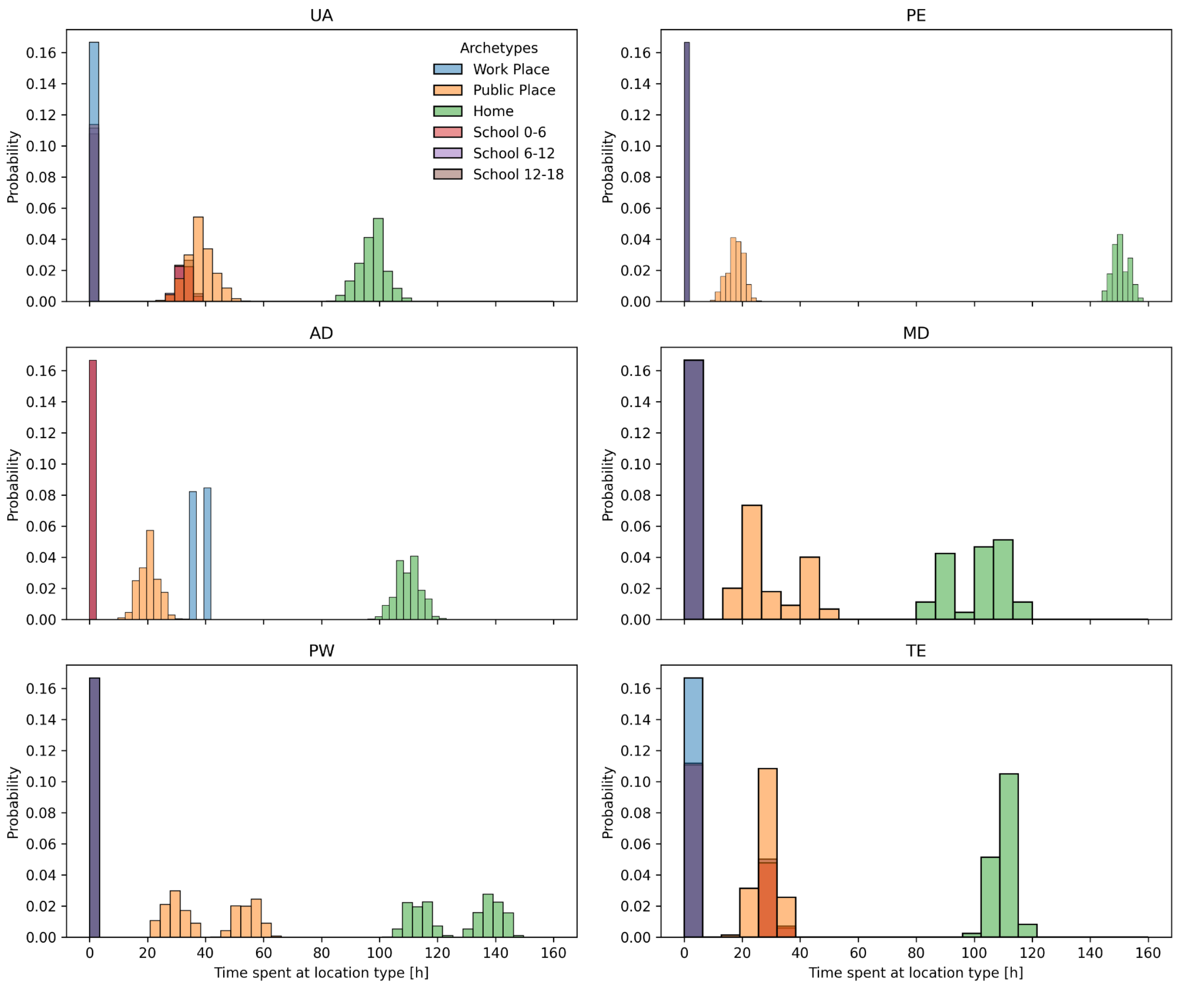

Figure S7: Time spent within a location for all archetypes by type of the location. Archetypes: underage (UA), pensioner (PE), adult (AD), medical professional (MD), public worker (PW) and teacher (TE). Results from a baseline simulation of the german community Gangelt (DE).

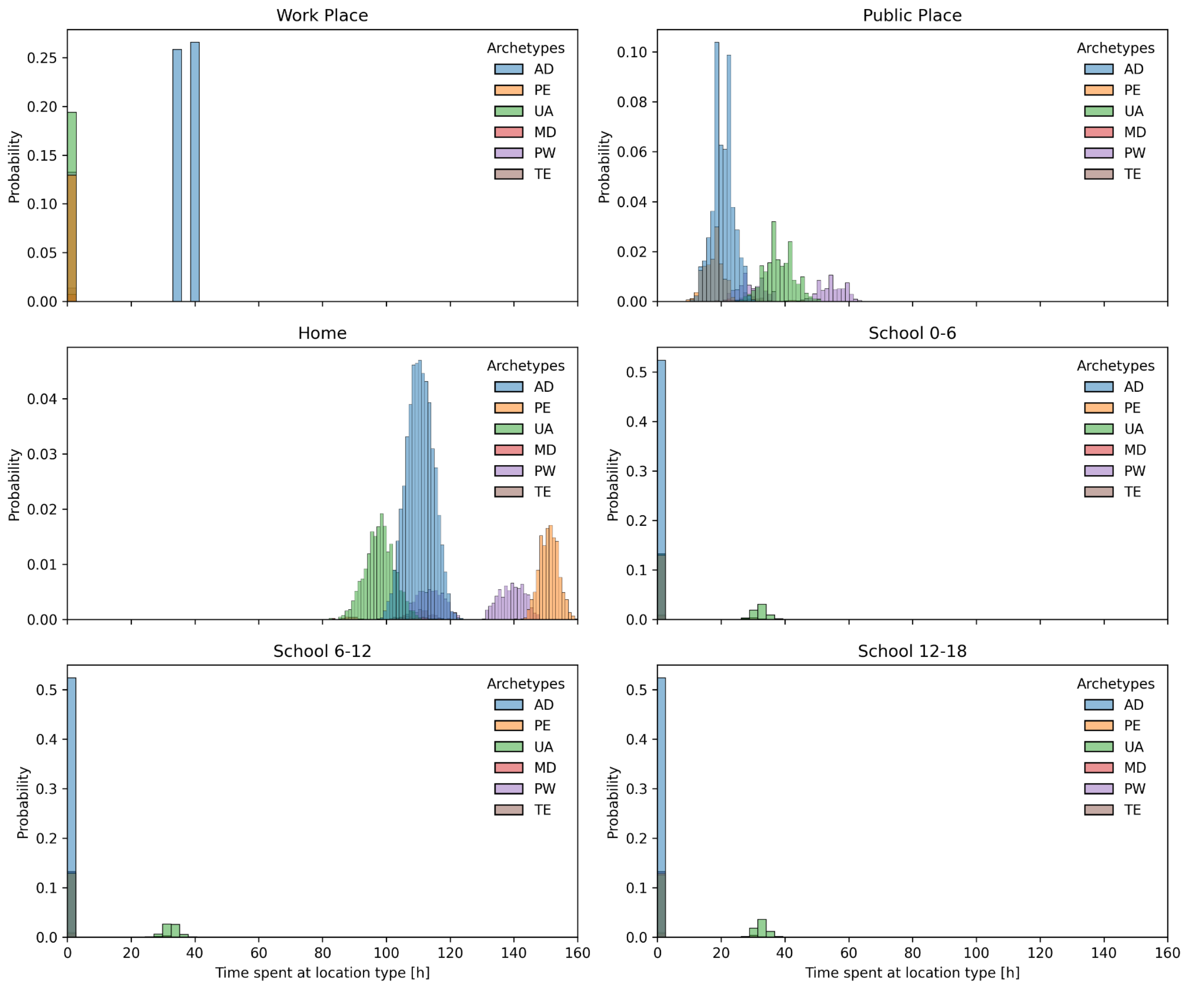

Figure S8: Time spent within a location for different location types by archetypes. Archetypes: underage (UA), pensioner (PE), adult (AD), medical professional (MD), public worker (PW) and teacher (TE). Results from a baseline simulation of the german community Gangelt (DE).

3.4 Health State Transitions

3.4.1 Principles

As introduced in **subsection 2.2.6**, in the GERDA-model agents can assume four main different health states: susceptible S, infected I, recovered R or dead D (**Fig. S1**, **Table S2**). Once infected, an agent can successively assume four different infection sub-states: The (a-/presymptomatic) infected I can possibly become diagnosed I^d, subsequently hospitalized I^d_H, and further referred to ICU I^d_{ICU}. Alternatively, the infected agent - generally from all of its different sub-states - can recover (transition to state R) or die (transition to state D) (**Table S3**).

Because all state transitions are irreversible (compare unidirectional arrows in **Fig. S1**), this leads to a total number of thirteen possible health state transitions. However, due to the lack

of sufficient data on reinfection with SARS-CoV-2 when already having recovered from it, represented by state transition X_{61}, loss of immunity is not considered in the presented GERDA model. This is technically achieved by setting the respective cumulative state transition probability P_{61}^{c} to zero. Furthermore, we exclude cases of dying from COVID-19 without being recognized, assuming that any deceased gets tested and a positively tested deceased is counted as COVID-19-related death. The cumulative probability P_{72}^{c} of the corresponding state transition X_{72} is set to zero. Transitions with probability zero are depicted by dotted arrows in **Fig. S1** and omitted in the tables of the following subsections.

For the remaining eleven state transitions, simulation of GERDA requires not only cumulative state transition probabilities P_{ij}^{c} (i.e. how likely is it that a diagnosed individual has to got to hospital), but hourly resolved conditional transition probabilities p_{ijt}^{X} for transition from state i to state j at time t. These probabilities depend on the duration of the infection and the time being in the current state, respectively. These probabilities also depend on the age of the agent. The following subsections outline how data from different sources have been combined, complemented with educated guesses, and processed to calculate those conditional hourly probabilities p_{ijt}^{X} for all state transitions and different age groups.

3.4.2 Input Data for Health State Transition Rates

If referenced by RKI Factsheet, we refer to the weekly status report of the Robert-Koch-Institute (RKI) from the 14th of March 2020 [39]. From this factsheet, we extracted information related to COVID-19 provided in **Tables S15, S16, S17** given in the separate file **Supplementary Tables**:

- **Table S15: Reported COVID-19 cases.** Source: RKI Factsheet [39]. 201 cases have been omitted due to lacking age information. For the remaining cases we calculated the total number (age group 'all').
- **Table S16: Deceased COVID-19 cases.** Source: RKI Factsheet [39]. Six cases were omitted due to incomplete information. For the rest we calculated the gender-independent numbers (column 'total') and the overall cases (age group 'all').
- **Table S17: Hospitalization information for COVID-19 cases.** Source: RKI Factsheet [39].

Age distributions not included in the RKI Factsheet have been taken from [40].

- **Table S18: Age group dependent information on hospitalization and referral to ICU.** Source: [40].

German Census data is used to determine the demography of our susceptible model population (state S_1) and, due to lack of age-dependent data on infection probability, also for infected agents (state S_2).

- **Table S19: Demography Germany 2011.** Source: German Zensus 2011 database.

To estimate the diagnosis probability (P_{23}^{c}), we used the homepage of the municipality Gangelt (https://www.gangelt.de/news/226-erster-corona-fall-in-nrw) and an early report about COVID-19 dynamics in Gangelt from [41]. Gangelt, a small town in the district of Heinsberg, was one of the first ‘Corona hotspots’ in Germany, where a lot of residents got infected during a large carnival event (“Kappensitzung”, Feb 15, 2020). Being of high interest for the understanding of SARS-CoV-2 and COVID-19, the entire population of Gangelt got tested, giving rise to the usually unknown dark number of infected individuals and, subsequently, the total diagnosis probability including not only symptomatic but also cases diagnosed based on contact information.

The usage of the above introduced data, the required assumptions, and the calculations applied to ultimately obtain age-dependent conditional probabilities p_{ijt}^{X} for all possible state transitions X_{ij} between states S_i and S_j per time step t are outlined in the following subsections.

3.4.3 Probability Calculations

3.4.3.1 Age-independent state transition probabilities

In **subsection 2.2.7**, we explained how to calculate cumulative and time-dependent conditional probabilities for the transitions between different states. Here and below, we outline how to convert input data with different levels of detail into the input information for the state transition probabilities required for GERDA simulation.

First, we extracted the age-independent cumulative state transition probability P_{ij}^{c} from the data listed in **subsection 3.4.2**. For the cases released from ICU, we know exactly how many have been discharged alive and how many dead, resulting in P_{56}^{c} and P_{57}^{c}. For cases with hospitalization information, we know how many are hospitalized (resulting in P_{34}^{c}) and how many of them are referred to ICU (resulting in P_{45}^{c}). For specific transitions, namely, we can derive the probabilities directly from the available data as follows.

Since we know how many people died out of all reported cases (see **Table S16**) and how many are released dead from ICU (I^{d}_{ICU}→D, X_{57}) we can infer the total number of people that died either in hospital (I^{d}_{H}→D,X_{47}) or somewhere else (I^{d}→D,X_{37}). However, we do not know the exact numbers for these two state transitions separately. We assume that people with more severe symptoms are people with a more severe disease progression. Those people are more likely to be referred to hospital, and more likely to die. Consequently, it should be more likely to die from state I^{d}_{H} than from state I^{d}. For lack of other information, we assumed that 90% of the deaths not occuring in ICU occur in hospitals. For the given numbers, this results in 1657 people who died in hospital but not in ICU and 184 people who died elsewhere. The number of reported cases serves as the reference group for the 184 people who died elsewhere and by knowing the probability to get hospitalized, we can calculate the reference group for the 1657 people who died in hospital, i.e. the number of people referred to hospital

assuming hospitalization information for all reported cases. This allows to calculate the respective cumulative state transition probabilities P^c_{37} and P^c_{47}.

The probability to get diagnosed (P^c_{23}) was inferred from two different sources, a report from [41] and the Gangelt homepage (https://www.gangelt.de/news/226- erster-corona-fall-in-nrw). Assuming further that nobody remains in one of the different infected substates but that everybody eventually leaves this state, we could infer the remaining cumulative state transition probabilities P^c_{26}, P^c_{36}, and P^c_{46}, i.e., the probabilities to recover from states S_2 (I), S_3 (I^d), and S_4 (I^d_H).

transition	symbol	value	reference	comments
S → I	P^c_{12}	*variable*	-	*see subsection 2.2.5*
I → I^d	P^c_{23}	*0.3*	*[41], Gangelt homepage*	-
I → R	P^c_{26}	*0.7*	-	*remaining fraction of infected*
I → D	P^c_{27}	*0*	-	*COVID-19 related deaths automatically count in reported cases as well*
I^d → I^d_H	P^c_{34}	*0.15*	*Table S17*	*hospitalized fraction (reference group: cases with hospitalization info)*
I^d → R	P^c_{36}	*0.84*	-	*remaining fraction of diagnosed*
I^d → D	P^c_{37}	*0.0017*	*Tables S16, S17*	*assuming 10% of deaths not in ICU from Id state*
I^d_H → I^d_{ICU}	P^c_{45}	*0.39*	*Tables S17*	*ICU fraction from hospitalized*
I^d_H → R	P^c_{46}	*0.42*	-	*remaining fraction of hospitalized*

$I^d_H \rightarrow$ D	P^c_{47}	*0.19*	*Tables S16, S18*	*assuming 90% of deaths not in ICU from state* I^d_H
$I^d_{ICU} \rightarrow$ R	P^c_{56}	*0.71*	*Table S18*	*fraction released alive from ICU*
$I^d_{ICU} \rightarrow$ D	P^c_{57}	*0.29*	*Table S18*	*fraction released dead from ICU*
R → S	P^c_{61}	*0*	-	-

Table S20: State Transitions and corresponding overall (age- and time-independent) cumulative probabilities.

In contrast to the state transitions described above, which solely depend on the state the agent is in and the time spend in this state, the state transition S→I (X_{12}) depends on different factors namely

- the interaction with an infected agent in state I (schedules, locations)
- the infection time dependent infectivity of interaction partner in state I (simulation input parameter infectivity)

That was explained in **subsections 2.2.4** and **2.2.5** and results in P^c_{12} are the product of these two probabilities (compare **Equation 8**). The determination of the infectiousness is further detailed in **section 3.5**. The resulting cumulative state transition probabilities are summarized in **Table S20**.

3.4.3.2 Age-dependent state transition probabilities

To calculate age-dependent cumulative state transition probabilities $P^c_{ij}(age)$, in addition to the age-independent transition probabilities P^c_{ij} listed in **Table S20**, we require age distributions for the different states S_i with $i, j = 1, .., 7$. From the input data, we obtain three consensus age groups in addition to the age-independent group 'all' (**Sec. 3.4.2.1**):

- 0 - 59
- 60 - 79
- 80 - 99

Combining the information from German Zensus and RKI Factsheet provides the following (age-dependent) information for those consensus age groups (**Table S21**).

age group	people in Germany	reported cases	hospitalized cases	cases in ICU	cases released from ICU	cases released dead from ICU	deceased cases
0-59	58998759	87375	-	-	-	-	134
60-79	17007688	23795	-	-	-	-	922
80-99	4213248	11645	-	-	-	-	1737
all	80219695	122815	14554	5700	3253	952	2793

Table S21: Consensus age group dependent information combined from the different data sources described above (compare Tables S15-S19). *cases* refer to COVID-19 cases.

To calculate age distributions, we first require age-resolved case numbers N_i for all states $i \in \{1,\ldots,7\}$ (**Table S22**). One exception is state R (S_6), which emerges based on the others. As outlined above, the infection probability P_{12}^{c} is age-independent (compare **subsection 3.4.3.1**). Therefore, the information from **Table S21** provides the age frequencies N_i for the four states S (S_1), I (S_2), I^d (S_3), and D (S_7). For the remaining two states I^d_H (S_4) and I^d_{ICU} (S_5), we used age frequencies reported for a subset of hospitalized cases (case study size n=10021) in [40].

age group	num. of people in S and I	num. of people in I^d	num. of people in I^d_H	num. of people in I^d_{ICU}	reported COVID-19 related deaths
0-59	58998759	87375	2896	422	134
60-79	17007688	23795	3779	917	922
80-99	4213248	11645	3346	388	1737
all	80219695	122815	10021	1727	2793

Table S22: Age group frequencies obtained from diverse input data. Numbers of people in S and I, in I^d, and the reported deaths are taken from the RKI factsheet [39], numbers of people in I^d_H and in I^d_{ICU} from Karagiannidis et al. [40]. Please note, that as the numbers are obtained from different data sources, they cannot be related directly between each other.

By dividing the number of cases in an age group $N_i(age)$ by the number of cases in age group 'all' of the same state $N_i(all)$ the age-group distribution for a state *i* was calculated (**Table S23**).

age group	fraction in S and I	fraction in I^d	fraction in I^d_H	fraction in I^d_{ICU}	fraction in D
0-59	0.74	0.71	0.29	0.24	0.05
60-79	0.21	0.19	0.38	0.53	0.33
80-99	0.05	0.2	0.33	0.23	0.62
all	1	1	1	1	1

Table S23: Age group distributions for different health states. The age group-dependent number of people in the reference state (compare **Table S22**) has been divided by the number for age group 'all'.

With this information and the overall cumulative state transition probabilities from **Table S20**, we calculated age group-dependent cumulative state transition probabilities $P^c_{ij}(age)$. Because the numbers in **Tables S22** and **S23** cannot be related between all the different states directly, we first created an example with age frequencies consistent between the different states for age group 'all'. To this end, we applied the age-independent probabilities P^c_{ij} (compare **Table S20**) to a fictive infected population of size *n = 1000000*, starting from state $N_2(all)$, resulting in a number of people for each state: Out of state $N_2(all) = 1000000$ 30% get diagnosed, resulting in state $N_3(all) = 300000$. Out of those 15.44% end up in hospital, i.e. state $N_4(all) = 46313$. 39.16% are referred to ICU ($N_5(all) = 18138$) from which 29.27% are released dead ($N_7(all) = 5308$). By splitting the resulting numbers of people per state into their age group fractions (compare **Table S23**), we obtain age group-dependent estimates for the expected number of people in each state for the example scenario. For each state transition, the age group-dependent transition probability $P^c_{ij}(age)$ equals the number of people in the end state divided by the number of people in the start state:

$$P^c_{ij}(age) = \frac{N_j(age)}{N_i(age)}$$

Equation (9)

with $i,j \in \{1,\ldots,7\}$ and $age \in \{0-59, 60-79, 80-99, all\}$. State transition probabilities $P^c_{ij}(age) \neq 0$ are summarized in **Table S24**. Please be aware that rounding resulted in several probabilities appearing to be equal to zero while in reality they are small but non-zero.

age group	$I \rightarrow I^d$	$I \rightarrow R$	$I^d \rightarrow I^d_H$	$I^d \rightarrow R$	$I^d \rightarrow D$	$I^d_H \rightarrow I^d_{ICU}$	$I^d_H \rightarrow R$	$I^d_H \rightarrow D$	$I^d_{ICU} \rightarrow R$	$I^d_{ICU} \rightarrow D$
0-59	0.29	0.71	0.06	0.93	0.00	0.33	0.64	0.03	0.94	0.06

60-79	0.27	0.73	0.3	0.7	0.00	0.55	0.28	0.16	0.82	0.18
80-99	0.54	0.46	0.54	0.45	0.01	0.26	0.39	0.35	0.19	0.81
all	0.3	0.7	0.15	0.84	0.00	0.39	0.42	0.19	0.71	0.29

Table S24: Age group-dependent state transition probabilities P_{ij}^{c} for all state transitions X_{ij} with $i,j \in \{1,...,7\}$. Please note that probabilities equal to zero in this table only do so due to rounding, in **Tables S26 (A-D)** the same probabilities are given in %, thus avoiding the zeros.

By definition of a non-chronically disease state, this state is transient, i.e. the probability to remain in a state S_i with $i,j \in \{2,...,5\}$ vanishes in the long term. That means that the sum of cumulative state transition probabilities P_{ij}^{C} away from each infected (sub)state *i* must equal 1, i.e.:

$$1 = \sum_{j=1}^{7} \quad P_{ij}^{c} \;\; \forall i \in \{2,...,5\}\,.$$

Equation (10)

3.4.3.3 Time-Dependent Conditional State Transitions

As explained in **subsection 2.2.7**, each agent being in a certain state at a certain time has a probability to stay in that state or leave towards selected other states within the next time step. To this end, we calculated hourly transition probabilities that depend on how long the agent was already in the given state: p_{ijt}^{X} is the required conditional state transition probability per hour for all considered state transitions X_{ij}. Based on the age group-dependent cumulative transition probabilities P_{ij}^{C} (**Table S24**), we determined age-dependent conditional state transition probabilities for $p_{ijt}^{X}(age)$, using the following assumptions:

1. The transition occurs within a given maximum time (50 days).

2. The probability for each state transition X_{ij} is Gaussian distributed with mean at the reported or assumed average time μ_{ij}^{t}.

3. The standard deviation of this Gaussian distribution equals its mean, i.e. σ_{ij}^{t} = μ_{ij}^{t}

From the data, we extracted cumulative transition probabilities P_{ij}^{C} of a state transition X_{ij} in a defined time frame and the reported average time μ_{ij}^{t} for the transition to occur. For example, if 30 % of cases died after being transferred to ICU and half of them died during the first 10 days after being transferred to ICU, the cumulative transition probability P_{57}^{c}would be 0.3 and the mean time would be $\mu_{45}^{t} = 10\ [days]$.

From assumption 2 it follows that the transition probabilities are drawn from a probability mass functions (PMF), generated from a Gaussian probability distribution and weighted by P_{ij}^{c} of the respective transition.

$$P_{ijt}^{X} = P_{ij}^{c} \cdot PMF \;\; (t: \mu_{ij}^{t}, \sigma_{ij}^{t})$$

Equation (11)

The required weighted distributions were calculated from information about the average transition times determined by the model from [42], **Fig. 1**). For all distributions, we assume the maximum transition time to be 50 days and the mean day was taken from this reference.

Because the underlying model of an der Heiden & Buchholz does not consider death from states other than ICU, we assume the same total average time from time of infection until death for the different states. Due to irreversibility of state transitions it is not possible to be referred back to hospital from ICU in the GERDA model. Therefore, we assume the average duration in ICU (state I^{d}_{ICU}) before recovery to be 13 days (until the average release from hospital when referring to the time of infection). In summary, we used the mean transition days given in **Table S25** to generate Gaussian distributed transition probabilities P_{ijt}^{X}:

**transition**	**$I \rightarrow I^d$**	**$I \rightarrow R$**	**$I \rightarrow D$**	**$I^d \rightarrow I^d_H$**	**$I^d \rightarrow R$**	**$I^d \rightarrow D$**	**$I^d_H \rightarrow I^d_{ICU}$**	**$I^d_H \rightarrow R$**	**$I^d_H \rightarrow D$**	**$I^d_{ICU} \rightarrow R$**	**$I^d_{ICU} \rightarrow D$**
mean day	5	13	19	4	9	15	1	14	11	13	10

Table S25: Average day after state entering for a state transition to occur. For complete model input tables used to calculated the final hourly resolved dependent state transition probabilities P_{ijt}^{X} compare Supplementary Tables file, **Tables S26 (A-D)**.

From the weighted distributions and the age group-dependent overall probability to undergo a specific state transition, we calculated the conditional transition probabilities per time step as explained in **subsection 2.2.7**. An illustration of the resulting probabilities is provided in **Fig. S9**.

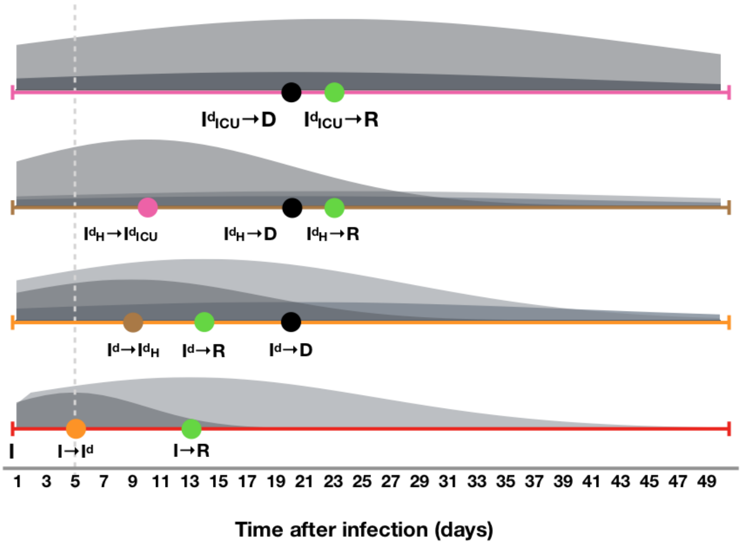

Figure S9: **Schematic representation of the conditional probability for transition from S_i to S_j (per time step, plotted against time after infection in days).** GERDA considers 11 transitions with non-zero probability. At each time step, an infected agent (states $S_2 - S_5$) can either remain in its current state, progress to another infected state or to one of the final states R (S_6) and D (S_7). Here, we show for a 64 year old agent the weighted distributions for the time-dependent probability to undergo a specific state transition. The probability for each state transition is Gaussian distributed with mean at the reported or assumed average time t_μ and standard deviation equal to mean, i.e., $t_\sigma = t_\mu$. The dashed line represents the average time to symptoms onset and the colored circles the mean occurrence times for state transitions, e.g. in green the average recovery periods for different COVID-19 progressions.

3.5 Infectiousness of an infected agent

The infectiousness $I_a^I(t)$ of an infected agent a is a function of time, starting with the time of infection. Background is that the reported time-dependent development of symptoms and viral load (representing the individual infectivity) do not coincide [43]. Instead, the infectivity peaks on day 2 after infection before symptoms onset on day 3. To represent pre- and asymptomatic transmission in GERDA, we used a Gaussian probability mass function to calculate the individual infectiousness. Its mean and standard deviation have been set to day 2, similar as described for the Gaussian distributions in **subsection 3.4.3** (**Table S26 (E), Fig. S10**). Although data on the timing of infectiousness is available [43], information on the intensity is still missing, therefore we weighted the obtained time-dependent probability distribution for the infectiousness by a global infection parameter k_I (compare next **section 3.6**).

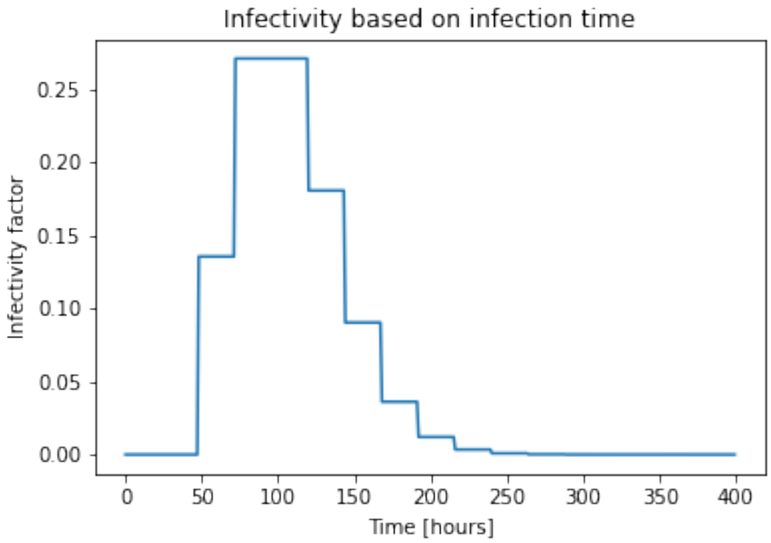

Figure S10: Time-dependent infectiousness. Infectiousness$I_a^I(t)$ of an infected agent a is a function of time, starting with the time of infection and including two days of zero infectiousness, reflecting the incubation period.

3.6 Additional Parameters

As a design principle, the parameter values required for the simulation of GERDA are not hard-coded, but simulation input. They can either be calculated from input data, as explained above, or they can be specified by the user when setting up the simulation. This allows us to change the parameter set between individual simulation runs, as explained below.

In **subsection 3.4.3**, we have described the time-dependent probabilities for state transitions. More parameters have to be set in order to enable a simulation. These are:

- the global infection parameter k_I (short: infectivity; required),
- the global interaction frequency μ (required),
- the non-compliance probability p^{ncomp} (optional).

Note that these parameters can be set either for the entire population or for population subgroups only.

Furthermore, start and end times for lockdown (closure) and reopening for all or for specific location types can be defined. For the required commands to set those parameters manually, please checkout the readme on our public GERDA Gitlab repository (https://tbp-klipp.science/GERDA-model/readme).

4 Simulation and Analysis of GERDA

Providing a simulation origin (an initialized MW) and the required and optional input parameters, GERDA can be applied for simulation of infection transmission throughout an agent population. At each simulation time step, for each agent it is resolved i) which of its putative interactions take place, ii) which of its realized interactions bear the potential of an infection transmission, and iii) which of its realized interactions effectively result in an infection transmission (**subsection 2.2.4**). In addition, for infected agents it is determined how the infection proceeds based on given state transition probabilities that depend on the time of infection and the agent's age. The calculation of cumulative time and age-dependent transition probabilities is detailed in **subsection 3.4.3**.

Alternatively, the simulation can also be started from a previous simulation output. This allows for the comparison of different scenarios starting from a given time point.

The simulation can be run from GitLab. The GitLab repository (https://tbp-klipp.science/GERDA-model/) also provides ample information about code, settings, and parameters. We provide a "cookbook" for interested users explaining which data and information to use in order to apply the model to different locations. The major functionalities are described in the following.

Below, we briefly summarize the basic output of the simulation of GERDA. Of course, it is always possible to add more functionalities for further analysis, which shall be omitted here.

4.1 Simulation time course and outputs

The time course of a simulation describes for each agent at each time step its

- location,
- health states (including infection sub-states),
- interaction partners,
- agent from which an infection was obtained.

Additional information, such as interaction and infection networks, frequencies and distributions of state transitions and durations for the transient infection states, or household-related transmissions can be directly inferred. This allows us to monitor infection spreading and disease progression on the individual level, thereby providing unprecedented insight into the effects of non-pharmaceutical intervention strategies, preventive measures like contact tracing, and the individual compliance.

It is possible to store complete and partial time courses. Time course simulations can be repeated (either full simulations or simulations starting from a given time point) in order to analyze the effect of stochasticity on the course of infection spreading.

4.1.1 Simulation object at final time step

Together with the locations the agent population at simulation end time is combined into a simulation object that shares most of its properties with a MW object. In addition, it contains the agent information (AI) including their transition times and the location information (LI) including all contacts and their timings.Simulation objects can be saved, loaded or resumed to allow for repeated simulations and implementations of interventions.

4.2 Simulation of specific scenarios

In order to investigate the effect of infection spreading, non-pharmaceutical interventions or vaccination strategies, it is possible to simulate specific scenarios.

The following scenarios are currently foreseen:

- Baseline scenario (spread of infection starting with one or few infected agents, **Fig. 1**)
  - Location-dependent behavior versus homogenous mixing (as control, see **section 4.9**)
- Non-pharmaceutical interventions, including
  - Lockdown (temporarily or continued, **Fig. 3** and **section 4.6**)
  - Selective lockdown of specific location types
  - Lockdown and full or selective reopening (**section 4.6**)
  - Re-infection, test for community protection
- Non-compliance with non-pharmaceutical interventions such as lockdown (**Fig. 1**, **section 4.6**)
- Pharmaceutical interventions (immunization scenarios, **section 4.8**)
- Combinations of the above

In the baseline scenario, we change the state of 4 random individuals from S to I and let the infections spread through the population according to the stochastic interactions that agents have when visiting locations corresponding to their schedule. No mitigation measures are applied. Default parameter values are infectivity k_I=0.14 and global interactivity μ=2.

Lockdown describes in our model a situation, where agents stay at home instead of going to school, to workplaces or to public places. Agents, who are scheduled to go to hospital, continue so (medical personal or hospitalized agents). Selected lockdown means that only one or two location types out of {schools, workplaces, public places} are closed and individuals stay at home instead of visiting them.

Reopening means that all agents in states S, I, or R return to their normal schedules or follow the quarantine (I^d) or hospitalization (I^d_H, and I^d_{ICU}) routine. Selective reopening indicates that the respective location can be visited again.

Non-compliance: We also investigated what happens if a number of individuals will not follow the order for closure of all locations. This reflects either non-compliance (e.g. civil

disobedience) or the fact that some people in systemically relevant jobs have to go to work or need to send their children to school (or daycare) to be able to go to work themselves. Already small levels of non-compliance have severe effects (**section 4.6**).

4.3 Transmission dynamics and state transition timings.

As a proof of consistency, we compared the empirical input distributions for state transition rates with the distributions of durations between specific state transitions resulting from a simulated baseline scenario as shown (**Fig. S11**).

Based on the state infection times, we estimated the median (25%-75%) of the generation time (GT) to be 3.79 days (3.08 days - 4.75 days) for the ancestral stain, which is in range of the reported values for the GT, though on the lower end[44,45;46)47]. For higher k_I, i.e. for more infectious variants GT decreases slightly to 3.58 and 3.42 for the Alpha and Delta variant (**Fig. S12**).

Assuming a constant incubation time the values for the generation time are also valid for the serial interval.

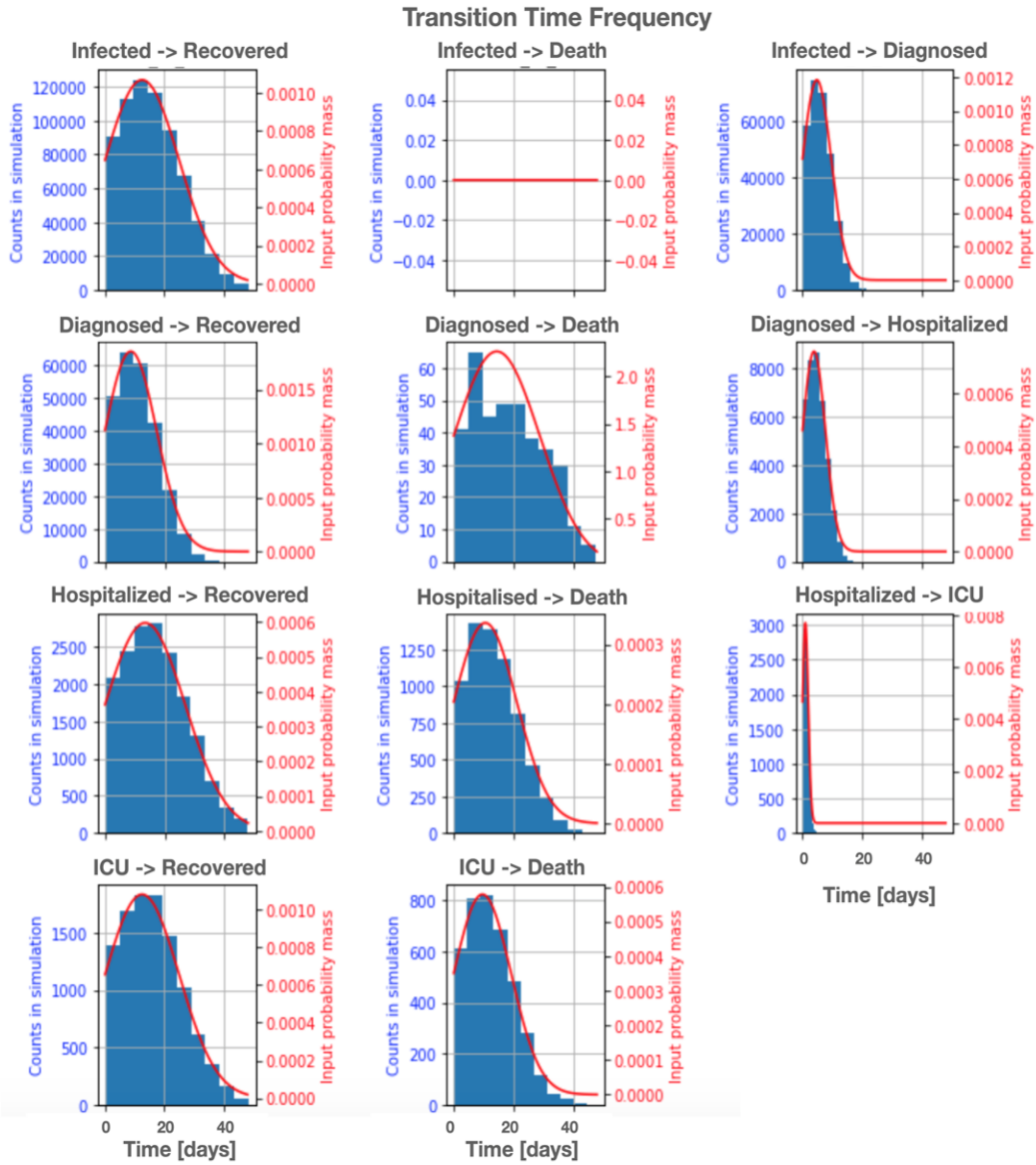

Figure S11: Comparison of input distributions for the agents' state transitions to distributions resulting from the simulations. Presented are the distributions of the indicated transitions.

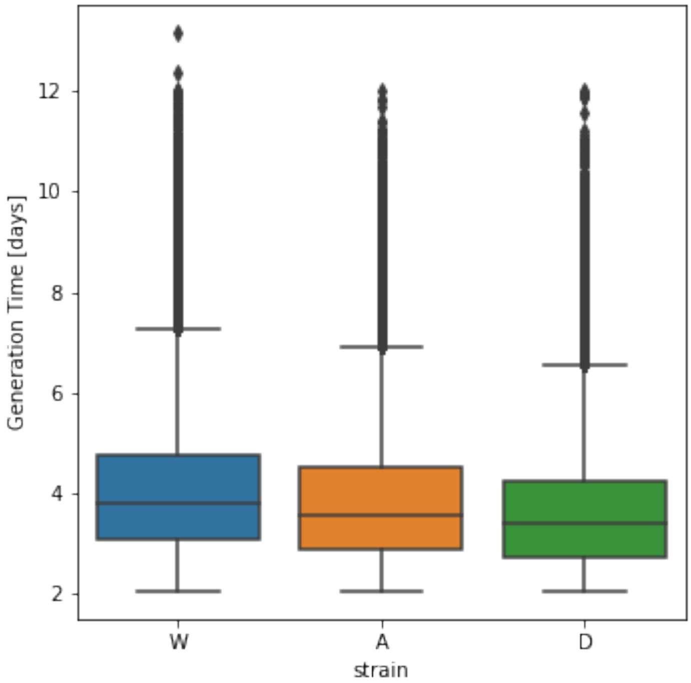

Figure S12: Obtained generation time (GT) decreases slightly with increasing infectiousness of the simulated viral strain. Median generation time decreases from 3.79 days to 3.58 days and 3.42 days for WT, Alpha and Delta Simulations of Gangelt (G, N=10.296 agents).

4.4 Community specificity

To test the importance of model initialization with community-specific data on the emerging simulation properties, we compared simulation results for Gangelt (Germany), Zikhron Ya'akov (Israel), and Tepoztlán (Mexico). In all three communities, children have the most unique contacts to children of the same age group (meeting in schools) while other agents also have contacts to different age groups (**Fig. S13, left column**). In contrast, when considering all contacts, the three communities differ especially for adults, which have much more age-nonspecific interactions in Tepoztlán than in Zikhron Ya'akov than in Gangelt, reflecting the different average household sizes (**section 3.2**, **Fig. S5COMP**) and family structures, e.g. having most multi-generation households in Mexico (**Fig. S13, right column**). Interestingly, when accessing the interactions per location type, in Zikhron Ya'akov agents have less interactions than in Gangelt and Tepoztlán (**Fig. S15**).

The numbers of infections per schedule type differ most for pensioners, who have far more infections in Tepoztlán than in the other two communities, which in similar but less significant manner holds for public workers, too. Also, for underaged agents numbers of infections differ significantly, with most infections in Gangelt and least in Tepoztlán, while for adults, teachers, and medical personnel infection numbers are comparable for all three communities (**Fig. S16**).

The infection per location type patterns are the same for all three communities with most infection transmissions at home, followed by public places, work, schools, and hospitals. The

only significant differences are in fraction sizes for home and work, which in Zikhron Ya’akov and Tepoztlán are bigger and smaller, respectively, when compared to Gangelt (**Fig. S17**).

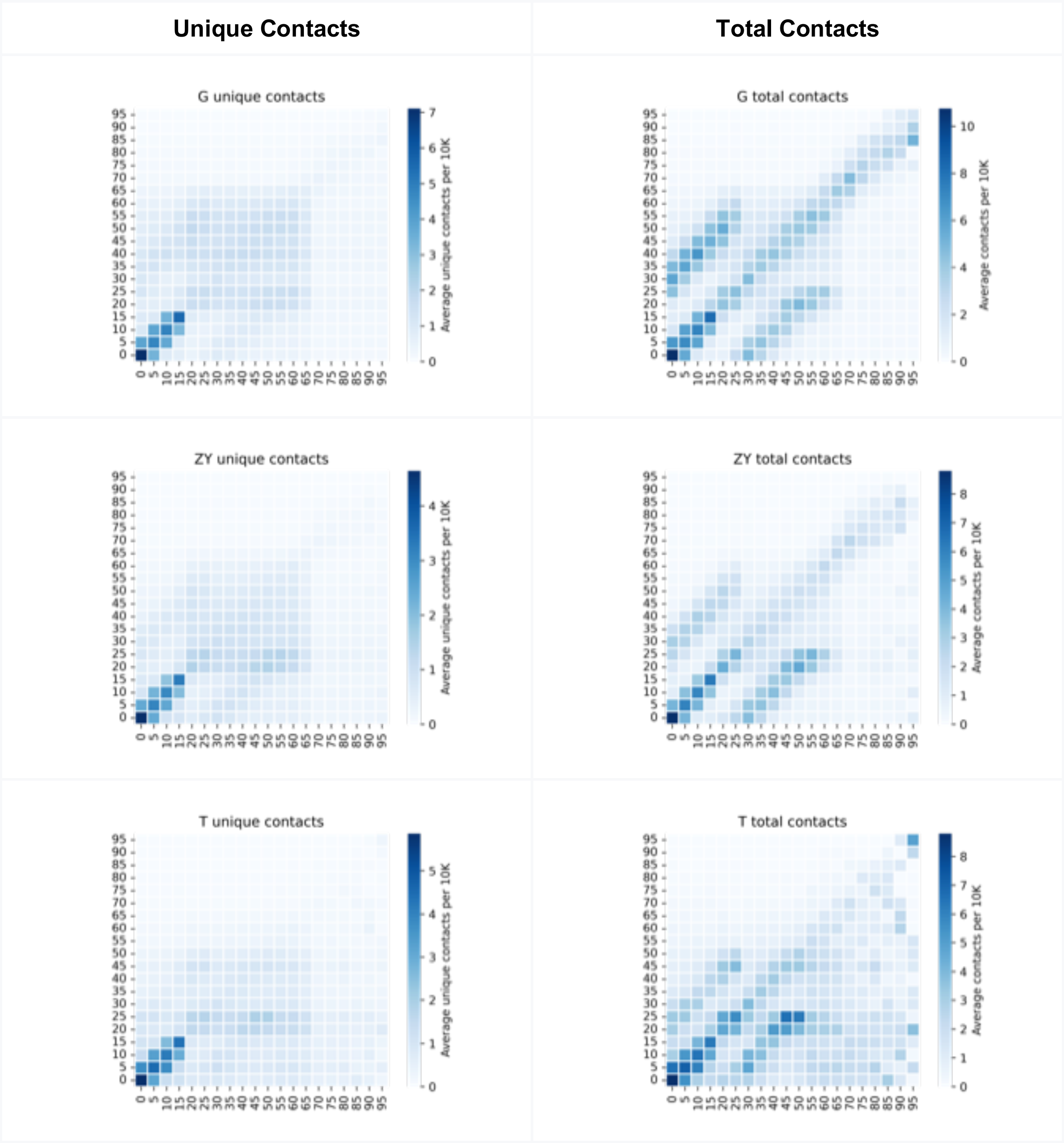

Figure S13: Contact pattern for three different parameterized communities: Gangelt (DE), Zikhron Ya’akov (IL), and Tepoztlán (MX). Simulations of Gangelt (G, N=10.296 agents), Zikhron Ya’akov (ZY, N=17.764 agents), and Tepoztlán (T, N=13.806 agents) and their patterns of average daily unique and total contacts (simulated with an average hourly interaction frequency μ=2). Age-groups are summarized in 5-year-cohorts

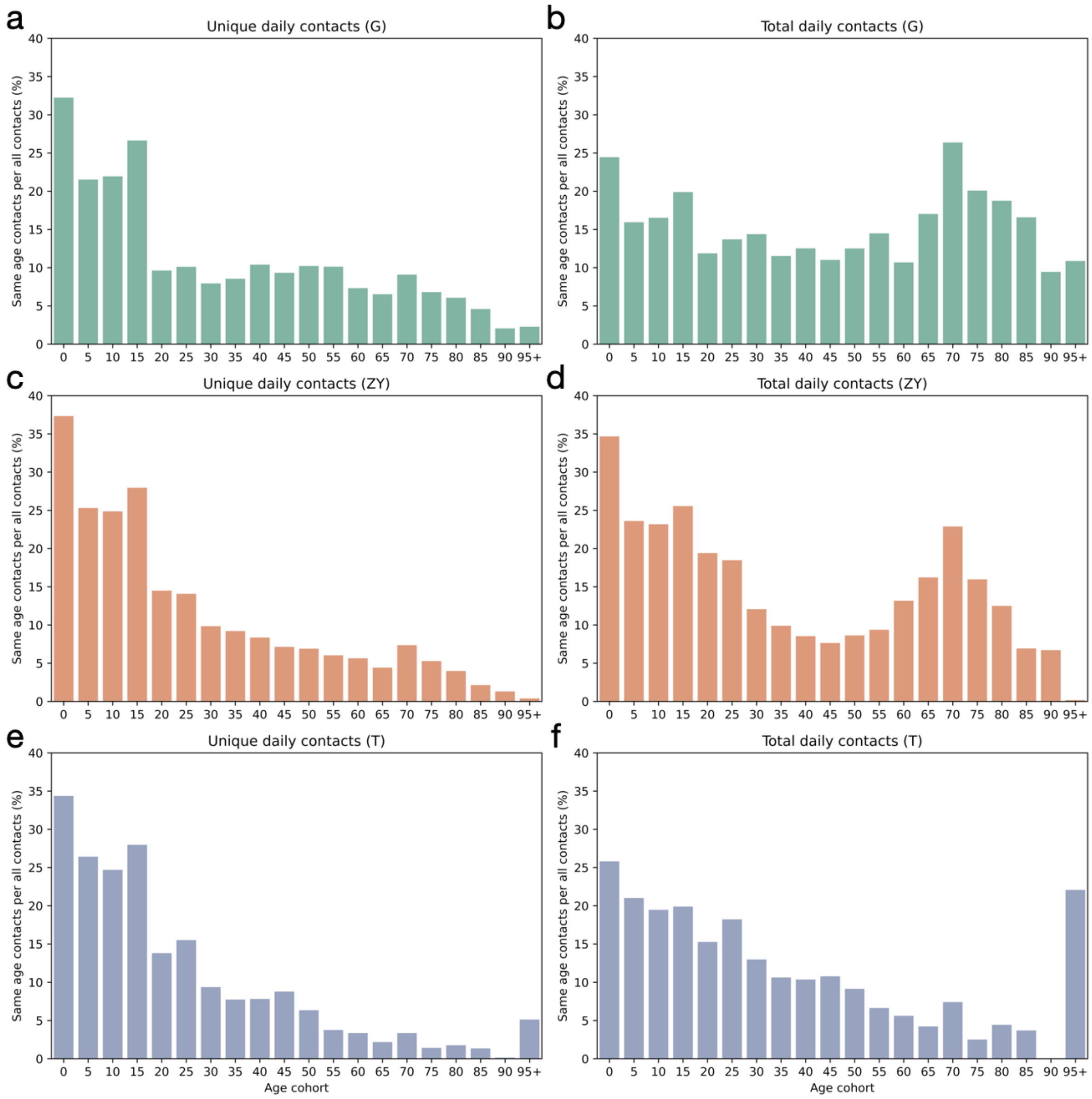

Figure S14: Fraction of contacts among age cohorts. Relative proportion of interactions with individuals of own age cohort, among daily total (b, d & f) and daily unique (a, c & e) contacts, for the three communities of Gangelt (G), Zikhron Ya'akov (ZY) and Tepoztlán (T).

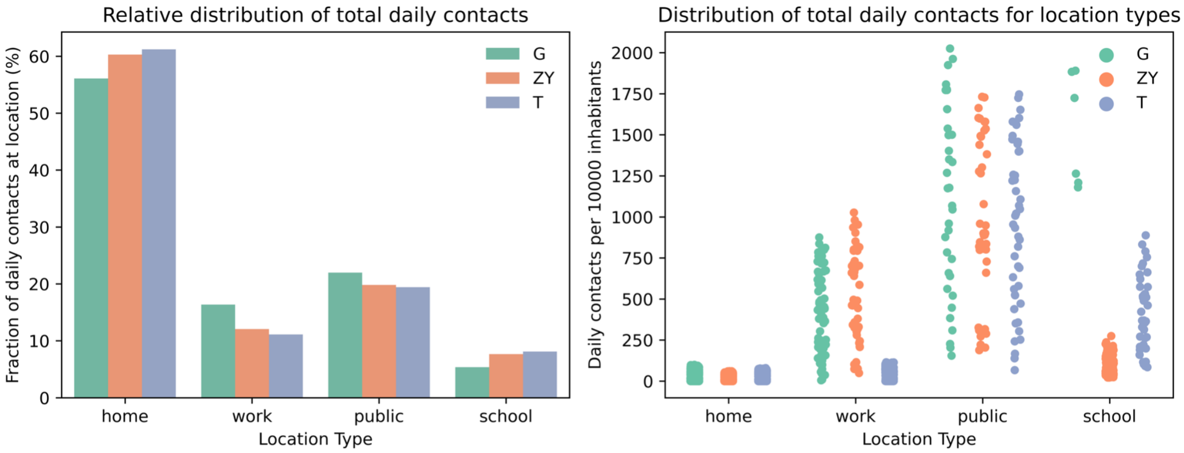

Figure S15: Interactions per location type. Left: Relative amount of total daily interactions, happening at all locations of the given type, for the three communities (average over 7 days). **Right:** Distribution of daily interactions, over all locations of the given type for the three communities, scaled to 10000 inhabitants.

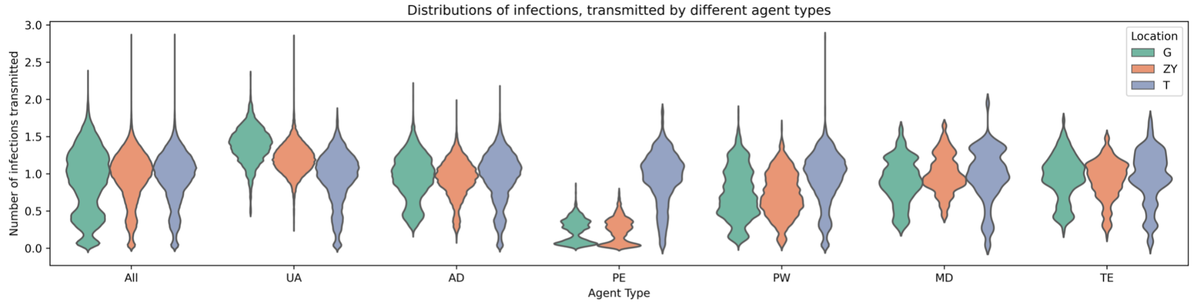

Figure S16: Agent type specific numbers of infections in different communities. Distributions of number of infections caused by individuals (mean over 96 replicate simulations), sorted by agent type (Whole population (All), adult (AD), public worker (PW) underaged (UA), pensioner (PE), teacher (TE), and medical personnel (MD)) for the communities of Gangelt (G, blue), Zikhron Ya'akov (Z, orange) and Tepoztlán (T, green).

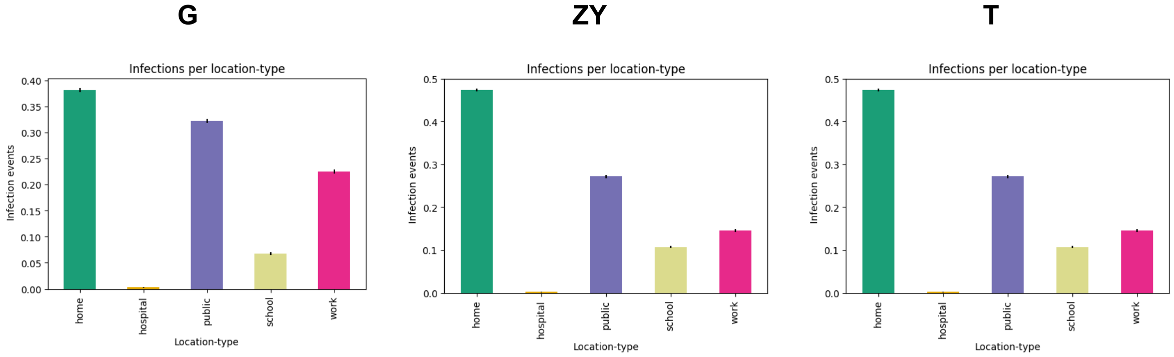

Figure S17: Infection per location type. Average number of infections per location type for Gangelt (G), Zikhron Ya'akov (Z) and Tepoztlán (T).

4.5 Bimodality

As our simulations show bimodality with respect to the occurrence of a second wave after certain interventions (non-pharmaceutical as well as pharmaceutical), we checked for indications for bimodality during the low incidence phase from June to July 2021 in Germany. We used KMeans Clustering [48,49] to identify different system behavior in July 2021 for cities of similar population density with similar 7 day incidences in June 2021. We used RKI data for German district level, namely COVID-19 case numbers per district (file RKI_COVID19, downloaded from the COVID-19 Datenhub at https://npgeo-corona-npgeo-de.hub.arcgis.com/datasets/dd4580c810204019a7b8eb3e0b329dd6_0/about) and district information (file RKI_Corona_Landkreise, downloaded from https://npgeo-corona-npgeo-de.hub.arcgis.com/datasets/917fc37a709542548cc3be077a786c17_0/explore?location=51.159939%2C10.714458%2C6.48&showTable=true), both downloaded on 22nd of October, 2021. Furthermore, we used RKI vaccination information (file COVID-19-Impfungen_in_Deutschland, downloaded on 27th of October, 2021, from the github repository of the RKI https://github.com/robert-koch-institut). From those district files, we excluded all cities and city districts, to assure comparability to our simulated communities.

To identify similar 7 day incidence progression during June 2021, we used the dynamic time warping (dtw) KMeans clustering algorithm of Python package *tslearn* for cluster identification[50]. Therefore, we used the time series data of the RKI, calculated the 7 day incidence from 29th of May, 2021, till 2nd of July, 2021, and applied the dtw KMeans algorithm with a cluster number of four. The identified four clusters are depicted in **Fig. S18, left column**, with the black line representing the dtw Barycenter Average of each cluster[50]. As we did not want to find particular trends across the data sets but to ideally find clusters with identical 7 day incidence progression in June 2021, we did not normalize for KMeans clustering t.

	Cluster 0	Cluster 1	Cluster 2	Cluster 3
avg. silhouette score nCluster = 2	0.307	0.313	0.562	0.717
avg. silhouette score nCluster = 3	0.198	0.197	0.461	0.325
avg. silhouette score nCluster = 4	0.196	0.217	0.339	0.262
avg. silhouette score nCluster = 5	0.199	0.199	0.223	0.157

Table S27: Average silhouette scores for each cluster using different numbers of cluster $n_{Cluster} \leq 5$.

Afterwards, we performed a second clustering for each individual cluster with the time series data from 2nd of July till 1st of August 2021. The respective cluster numbers are derived by comparing the avg. silhouette score for a range of $n_{Cluster}$ = 1, …, 10[51]. As can be seen in **Table S27**, the average silhouette score is the highest for $n_{Cluster}$ = 2, which we used to identify one subcluster with a lower dtw Barycenter Average (**Fig. S18, center column, denoted with 0**) and another subcluster with a higher dtw Barycenter Average (**Fig. S18, right column, denoted with 1**) for each cluster.

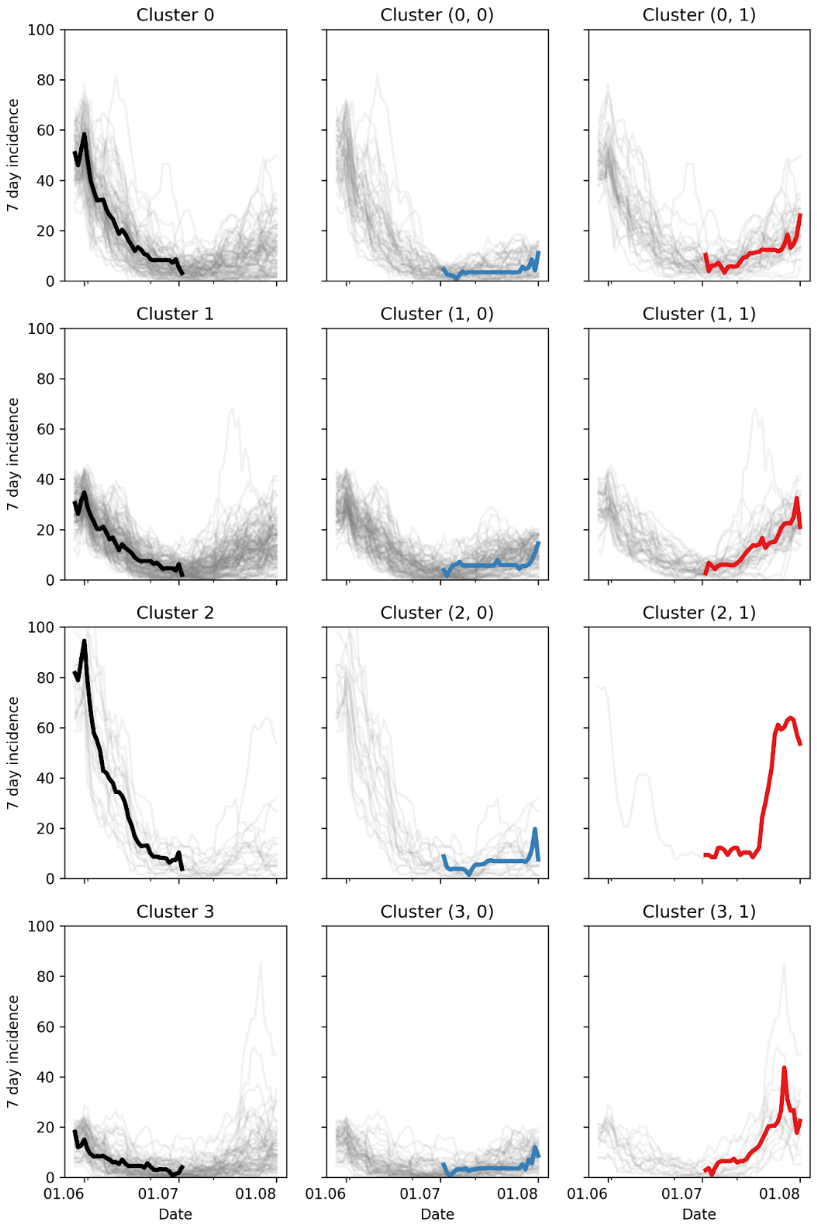

Figure S18: Visualization of clusters and their subclusters. In gray each district's 7 day incidence. In color Dynamic time warping Barycenter Average (DBA) of the cluster's time series data, in black first clustering (time series data used from 05/29/2021 - 07/02/2021, cluster number = 4), second clustering of individual cluster (time series data used from 07/02/2021 - 08/01/2021, cluster number = 2), in blue subcluster with lower average and in red subcluster with higher average.

Finally, we matched each district in a subcluster 0 to the best suited partner in the corresponding subcluster 1. As a comparison measure, we used the quadratic difference of the population density (δPop) and sort for the smallest value. Selected pairs from the different clusters are shown (**Fig. S19, columns 1 and 3**) aligned with the vaccination rates in the respective districts (**Fig. S19, right columns 2 and 4**).

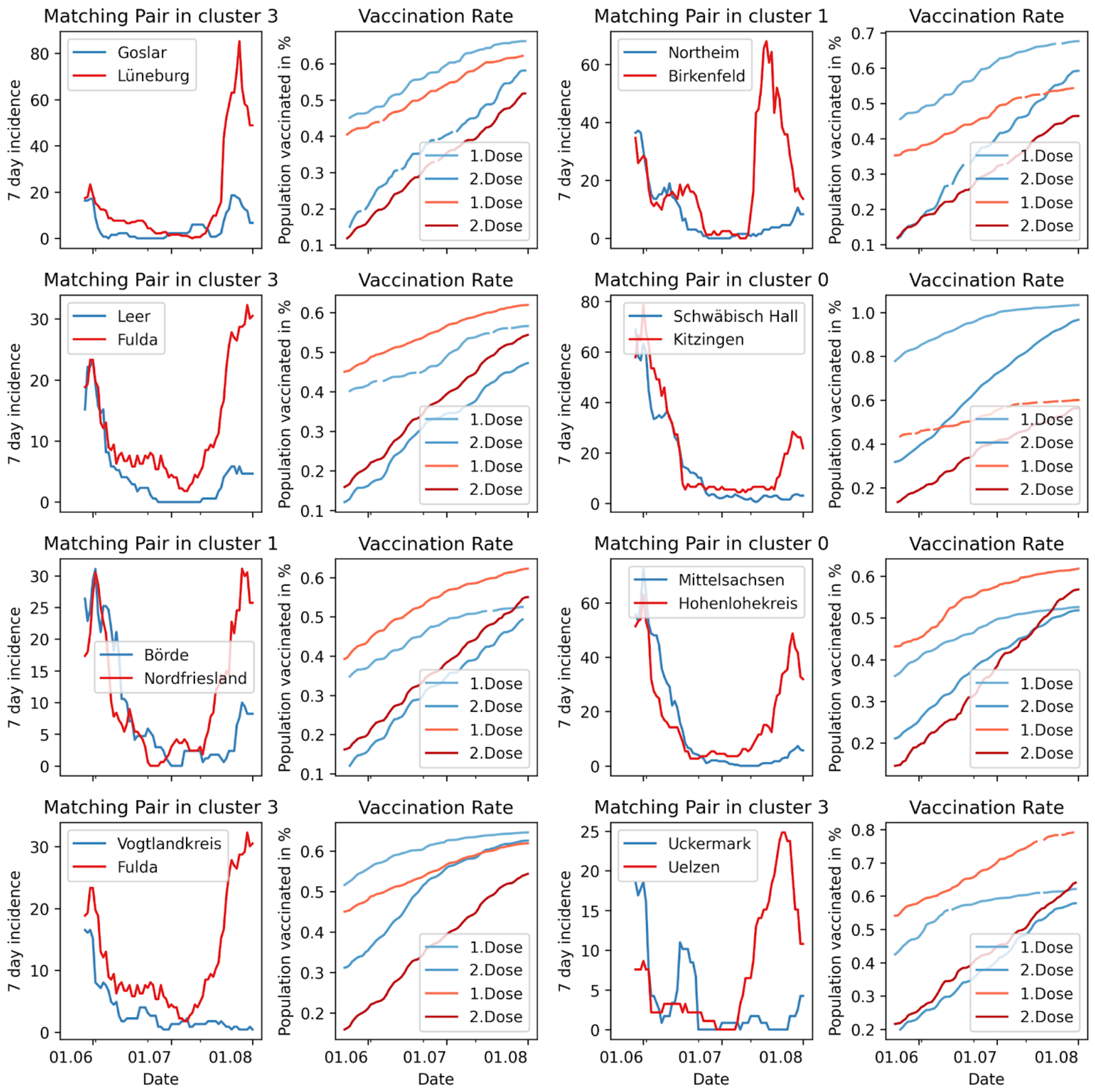

Figure S19: Examples for different system behavior of two districts with highest similarity score when comparing subclusters. Columns 1 and 3: graph of 7 day incidence; Columns 2 and 4: vaccination status of district. In blue from lower average subcluster, in red higher average subcluster, vaccination doses are indicated in the respective subplots.

4.6 Sensitivity analysis

In order to better understand the impact of the choice of parameter values as well as the capabilities of our model, we performed sensitivity analyses. Selected results are shown below:

- change start and end times of non-pharmaceutical intervention measures (**Fig. S20** - variation of lockdown start time, **Fig. S21 and Fig. S22** - variation of reopening times, **Fig. S23** - variation of reopening times for public places only**, Fig. S24** - variation of reopening times for schools only**, Fig. S25 -** variation of reopening times for workplaces only)
- apply specific measures to either specific age groups, groups with different occupations (e.g. pensioners), or specific locations.
- vary the fraction of vaccinated individuals at the start of simulation and test different vaccination efficacies (representing different vaccines), vaccination effects (vaccination models), and the influence of different strains on vaccination scenarios (**section 4.8**)
- study the impact of infection rate on attack rate and bimodality (**Fig. S26**)

Sensitivity analysis enables systematic comparison of model results with input data. It also points to specific behaviors of the model that only occur for specific parameter values.

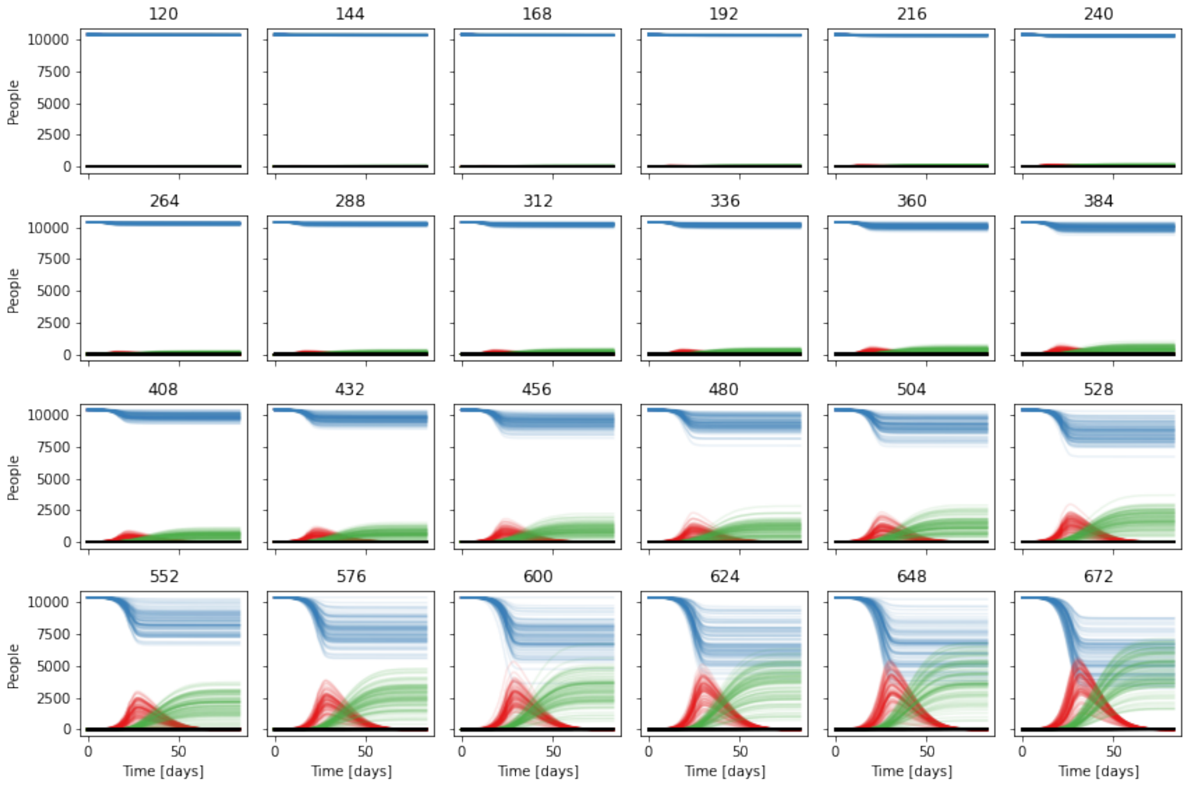

Figure S20: Lockdown start, time scan from t = 120 hours to t = 670 hours (WT, Gangelt). Resulting time courses for agent health states S (blue), I (red), R (green), and D (black) from 96 simulations for varying lockdown start times.

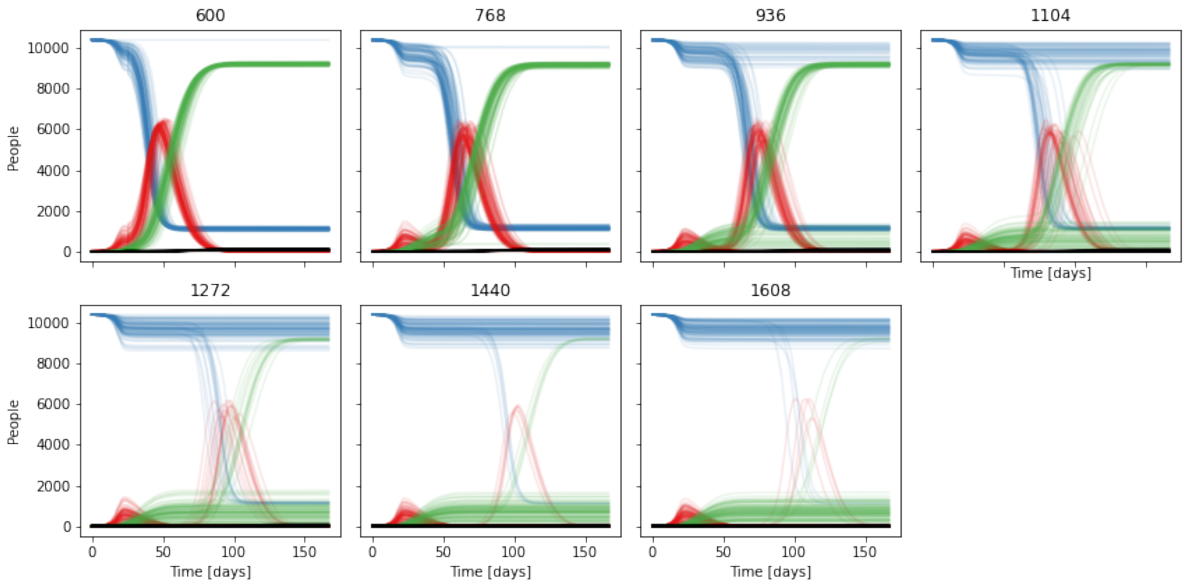

Figure S21: Reopening times scan from t = 600 hours (25 days) to t = 1608 (67 days) hours (reopen all; WT, Gangelt). Resulting time courses for agent health states S (blue), I (red), R (green), and D (black) from 96 simulations for varying reopening times after closing all at t=432.

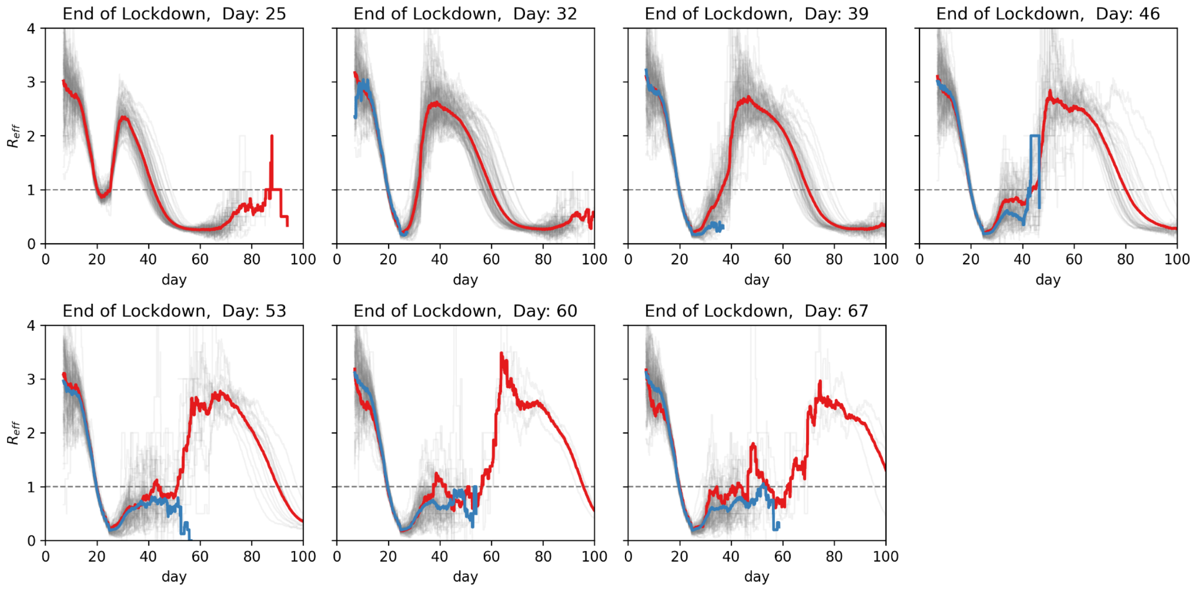

Figure S22: Reopening times scan from t = 600 hours to t = 1608 hours (reopen all, WT, Gangelt). R_{eff} time courses for N=96 repetitions in gray and average R_{eff} time courses for simulations with and without second outbreak, red and blue .

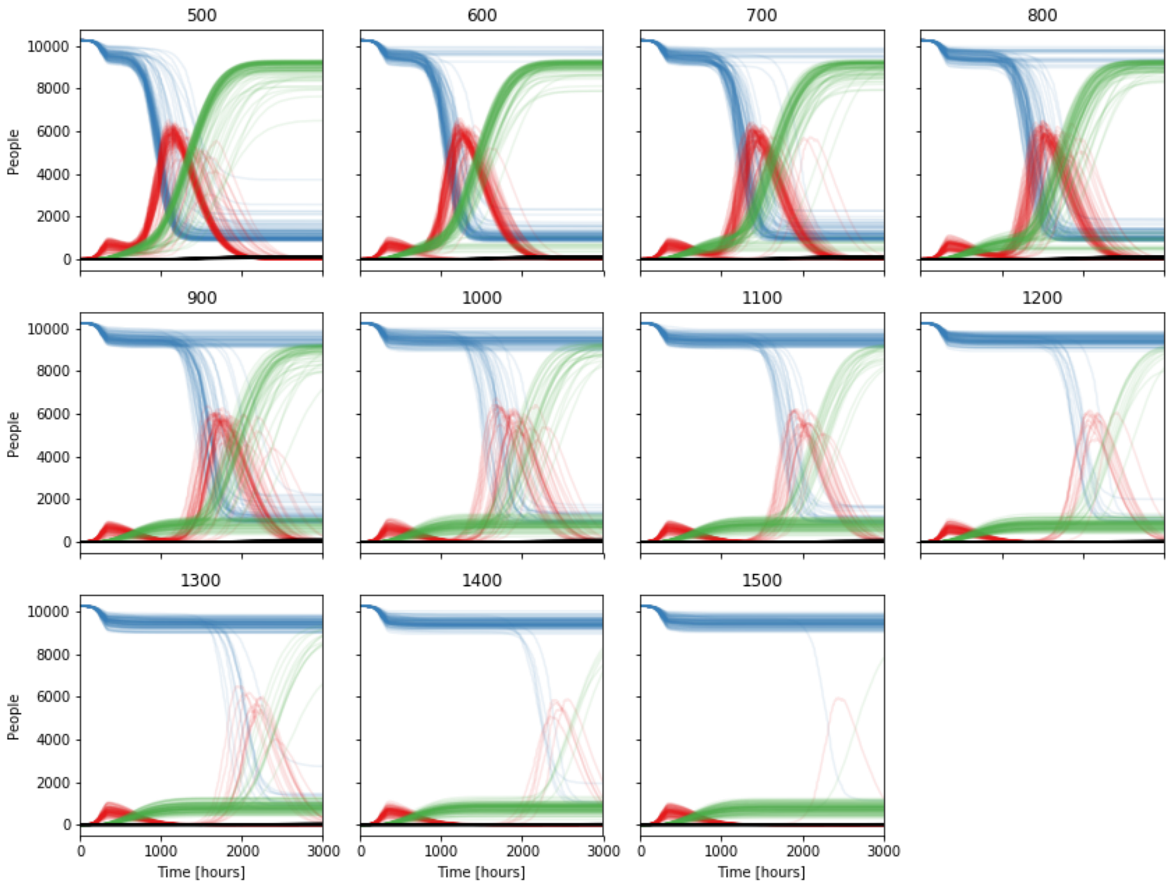

Figure S23: Reopening times scan from 500 to 1500 (reopen public places; WT, Gangelt). Resulting time courses for agent health states S (blue), I (red), R (green), and D (black) from 100 simulations for varying reopening times after closing all at t=200.

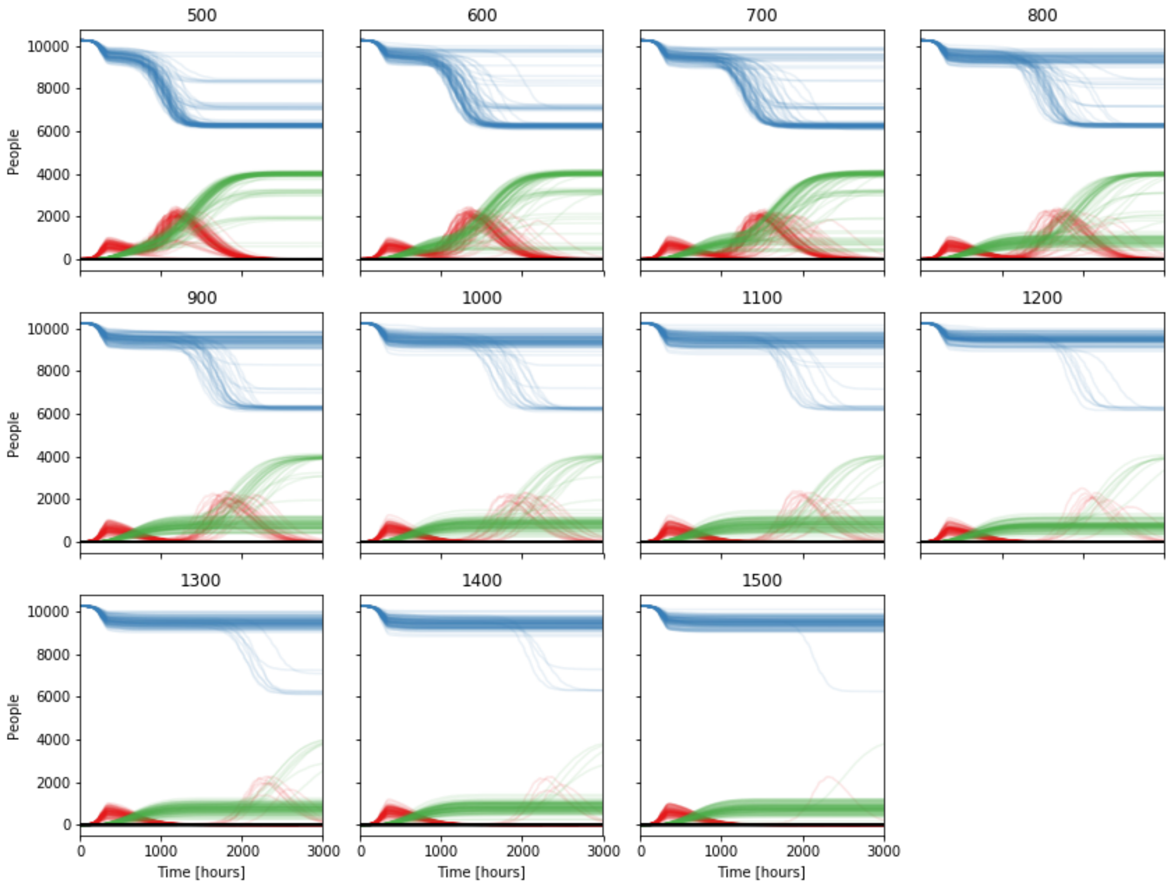

Figure S24: Reopening times scan from 500 to 1500 (reopen schools; WT, Gangelt). Resulting time courses for agent health states S (blue), I (red), R (green), and D (black) from 96 simulations for varying reopening times after closing all at t=200.

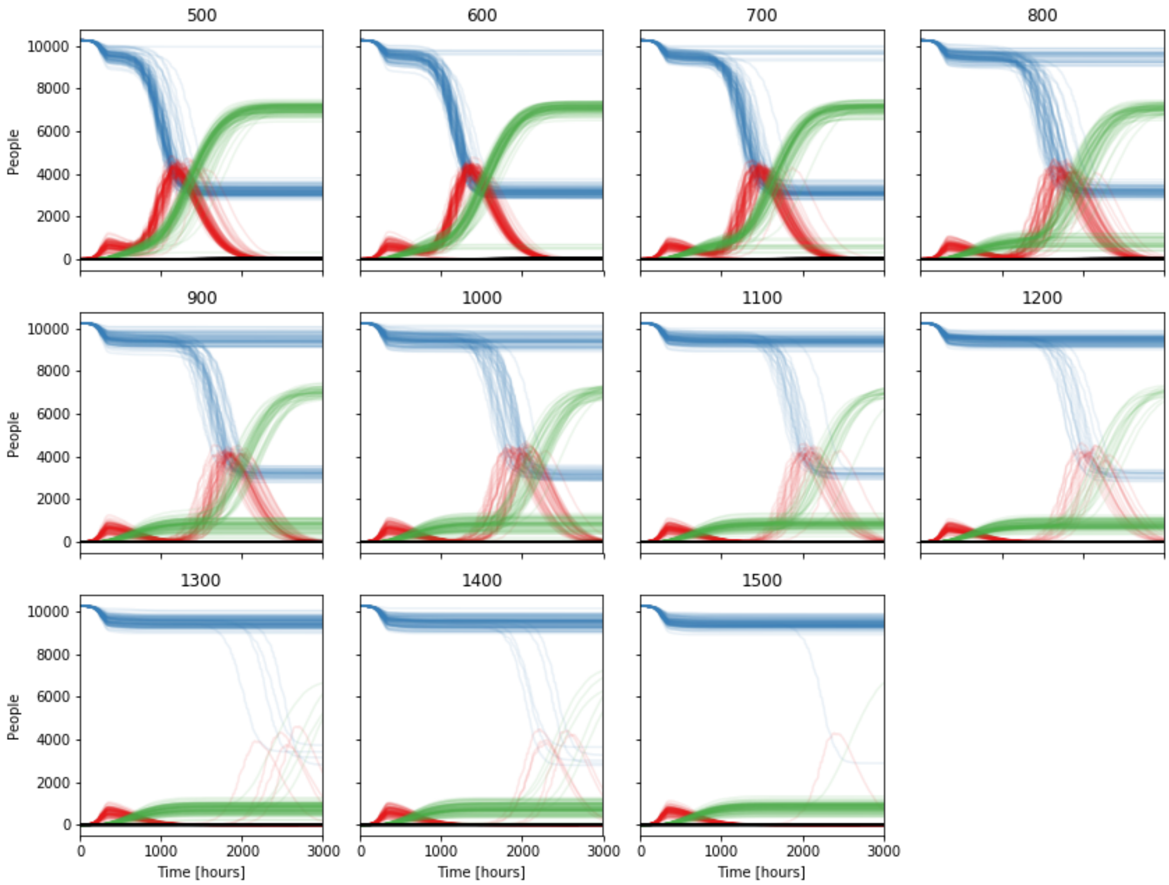

Figure S25: Reopening times scan from 500 to 1500 (reopen work; WT, Gangel). Resulting time courses for agent health states S (blue), I (red), R (green), and D (black) from 96 simulations for varying reopening times after closing all at t=200.

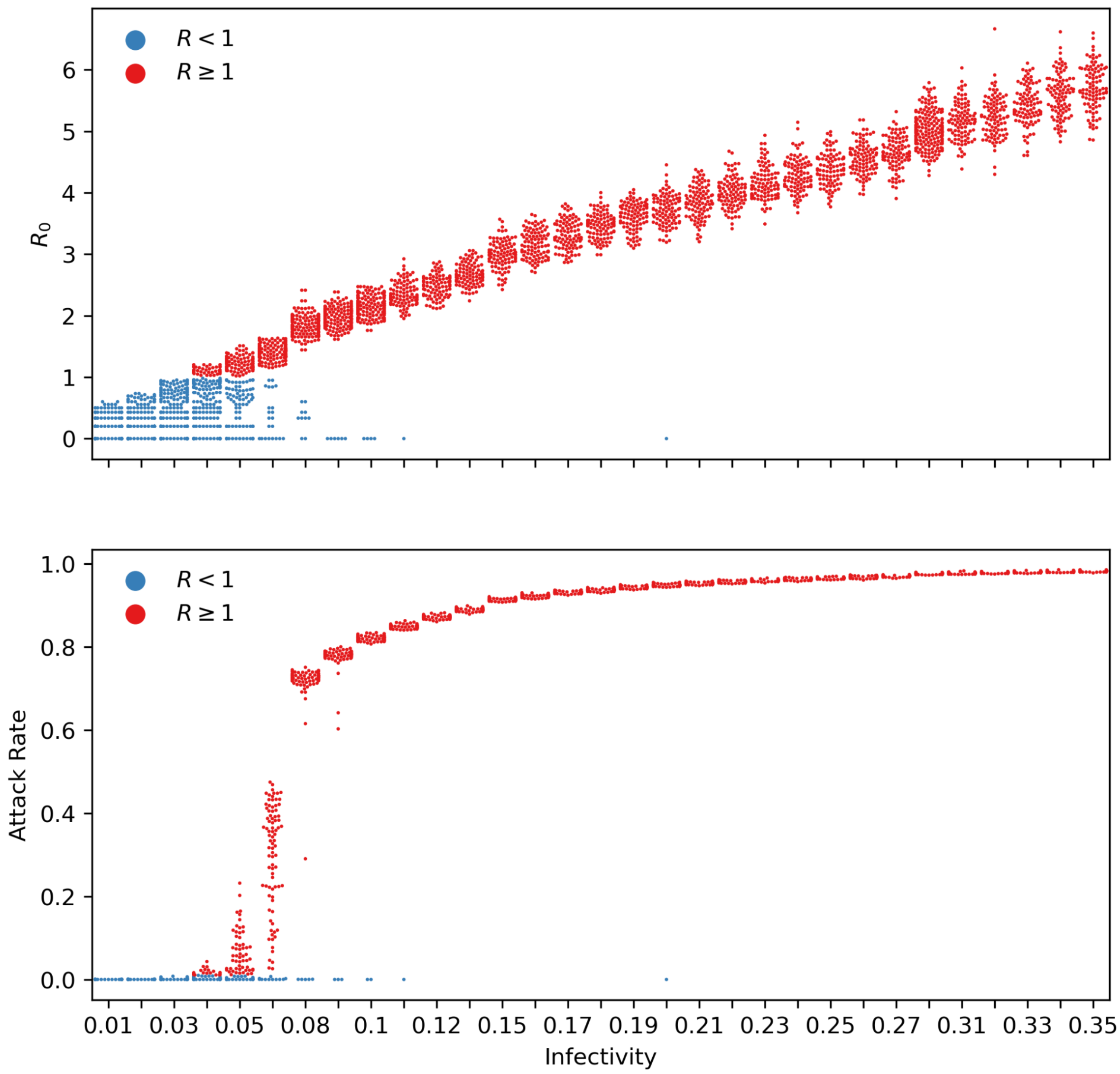

Figure S26: Impact of Infectivity on R_0 and Attack Rate (AR) (GangeIt). R_0 scales almost linearly with the infectivity k_I and shows higher variations for low and high k_I values. In contrast, AR shows a sigmoidal dependence on k_I. R_0 AR (total infected / initial susceptible) for 100 simulations and different k_I. Note bimodal outcome for the range $0.03 < k_I < 0.09$ for R_0 as well as AR.

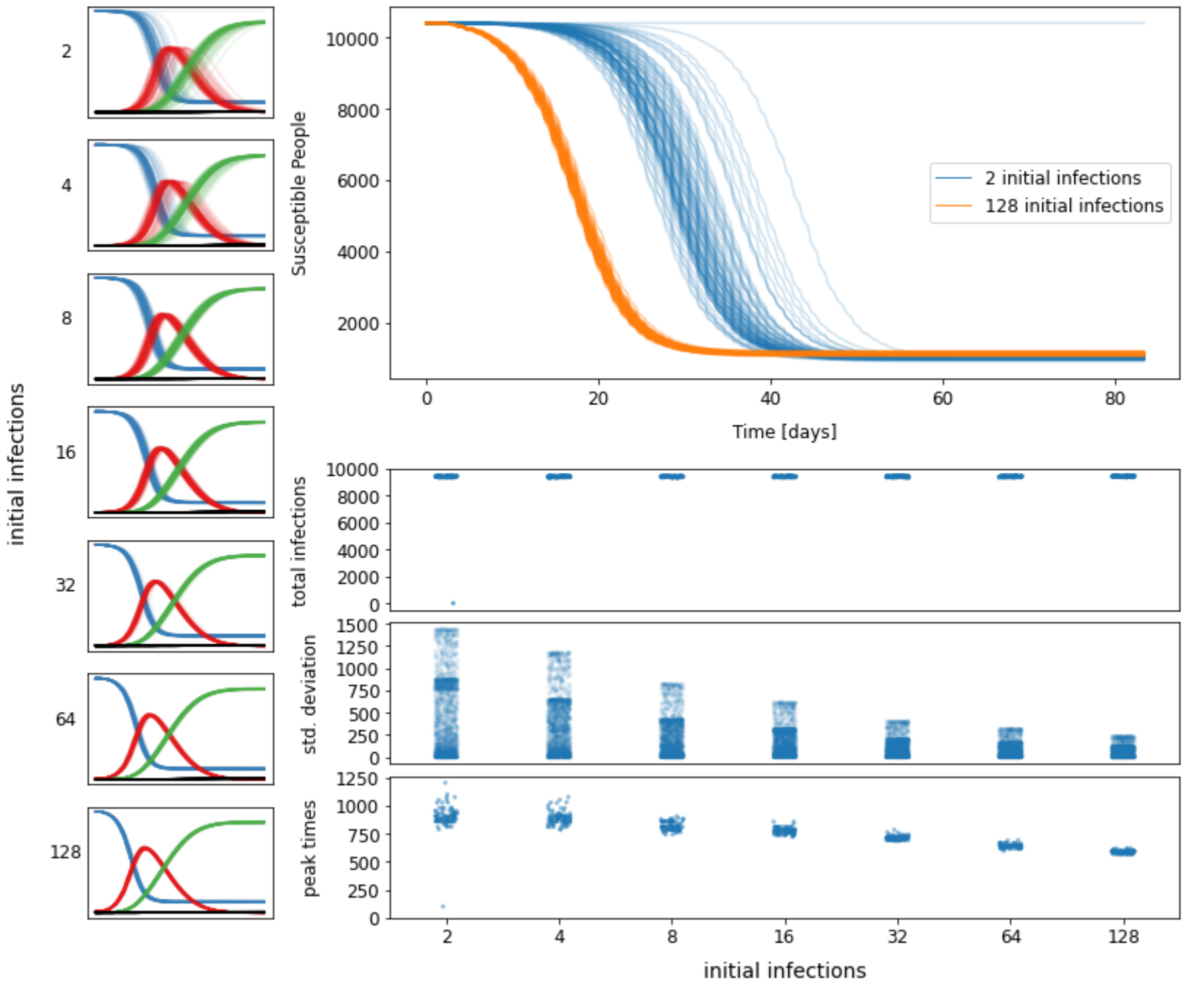

Figure S27: Impact of the number of initial infections. The left panel shows SIR time courses of base scenario simulations over 83 days with varying number of initial infections n_I between 2 and 128. On the upper right the susceptible time courses from the simulations with 2 and 128 initial infections are shown together. The lower right shows the evolution of total infections, standard deviations over all time points and infection peak times (in hours) from top to bottom, respectively. Each data point represents a single simulation run, except for the standard deviations that are computed for each time point over all corresponding simulations. We notice a shift of infection wave occurrences to earlier time points with an increasing number of initial infections. Also the deviation between single simulation runs decreases while the total number of infections stay at a similar level.

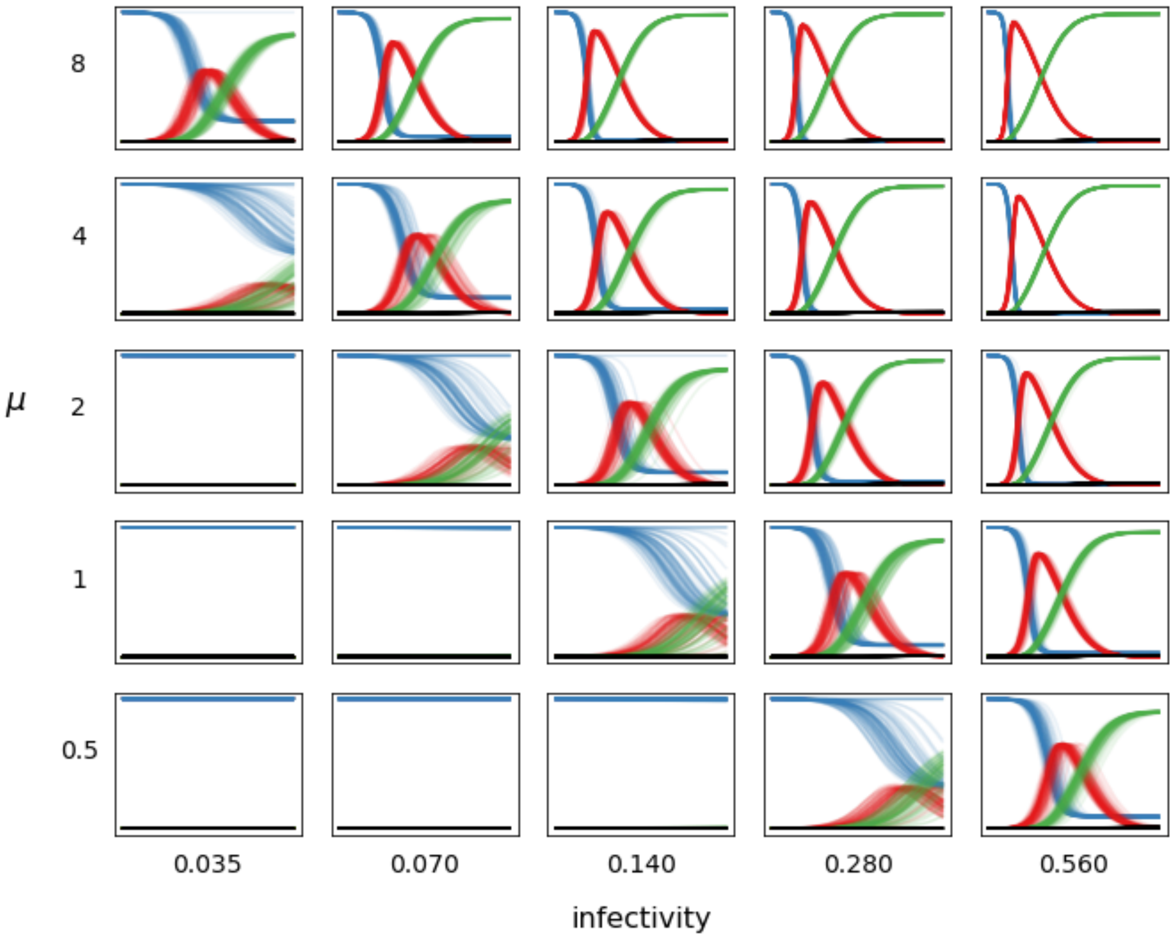

Figure S28: Combined impact of infectivity k_I and mean hourly interaction frequency μ. The SIR time courses of simulations with varying μ and infectivity k_I are shown in a matrix.The center of this matrix represents the simulation of the baseline scenario. To each side the value of infectivity is either halved (left) or doubled (right). Going up the value of μ is doubled, going down it is halved. Like this, the product $\mu * k_I$ is constant over the diagonals from upper left to lower right. We see that the simulations on each of these diagonals exhibit strong similarity. Going from lower left to upper right, total infections increase, the infection peaks shift to earlier time points and the variance between simulation runs decreases, given an infection wave occurs.

4.7 Network analysis

4.7.1 Interaction networks

Interaction and infection networks are emerging throughout a simulation time course. Since we are tracking the complete simulation time course we can reconstruct the (time-dependent) interaction network after a simulation. The complete network graph is built by adding new edges (and corresponding nodes if not yet existing) for each first appearance of a unique pairwise interaction. Also, it is possible to define specific time frames to create and analyze

subsets of interactions. Counting the number of occurrences for unique pairwise interactions provides a weighted graph.

It is important to notice that for each time step and each location a new interaction network is created on runtime, following the rules described in **subsection 2.2.4**. Each of these interaction networks is unique for the current time step and depends on the set of agents occupying each location at this specific time step. **Fig. S29** to **Fig. S32** illustrate such temporary interaction networks for different time steps and different locations of varying types. **Fig. S33** shows a distribution of average node degrees at one time step for different location types. Notably, these values distribute around the set mean interaction value μ, but can differ by more than 1 degree, resulting from differences in location occupancy, especially if there are less than three agents in one location at this time, limiting the maximum possible interactions of the agent(s) present in this location to 1 or even 0.

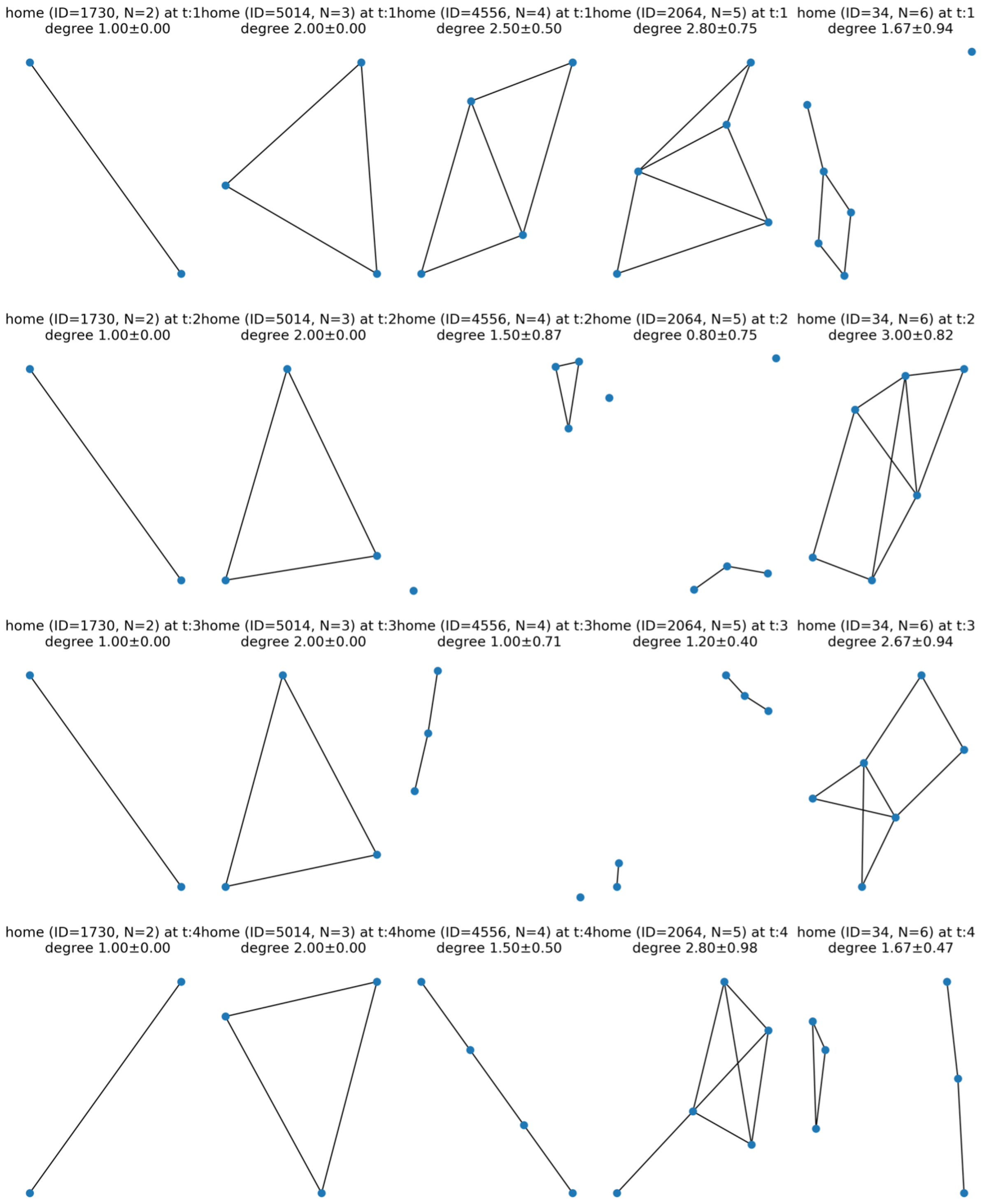

Figure S29: Networks of the contacts at specific times within homes of different household sizes. Exemplary graphs of the interaction networks in home locations (specified here by their ID), from a baseline simulation of the German community Gangelt (DE). N - number of agents present at the location; the node degree is given as mean +/- std. The household size increases from left to right from 2 to 6 household members.

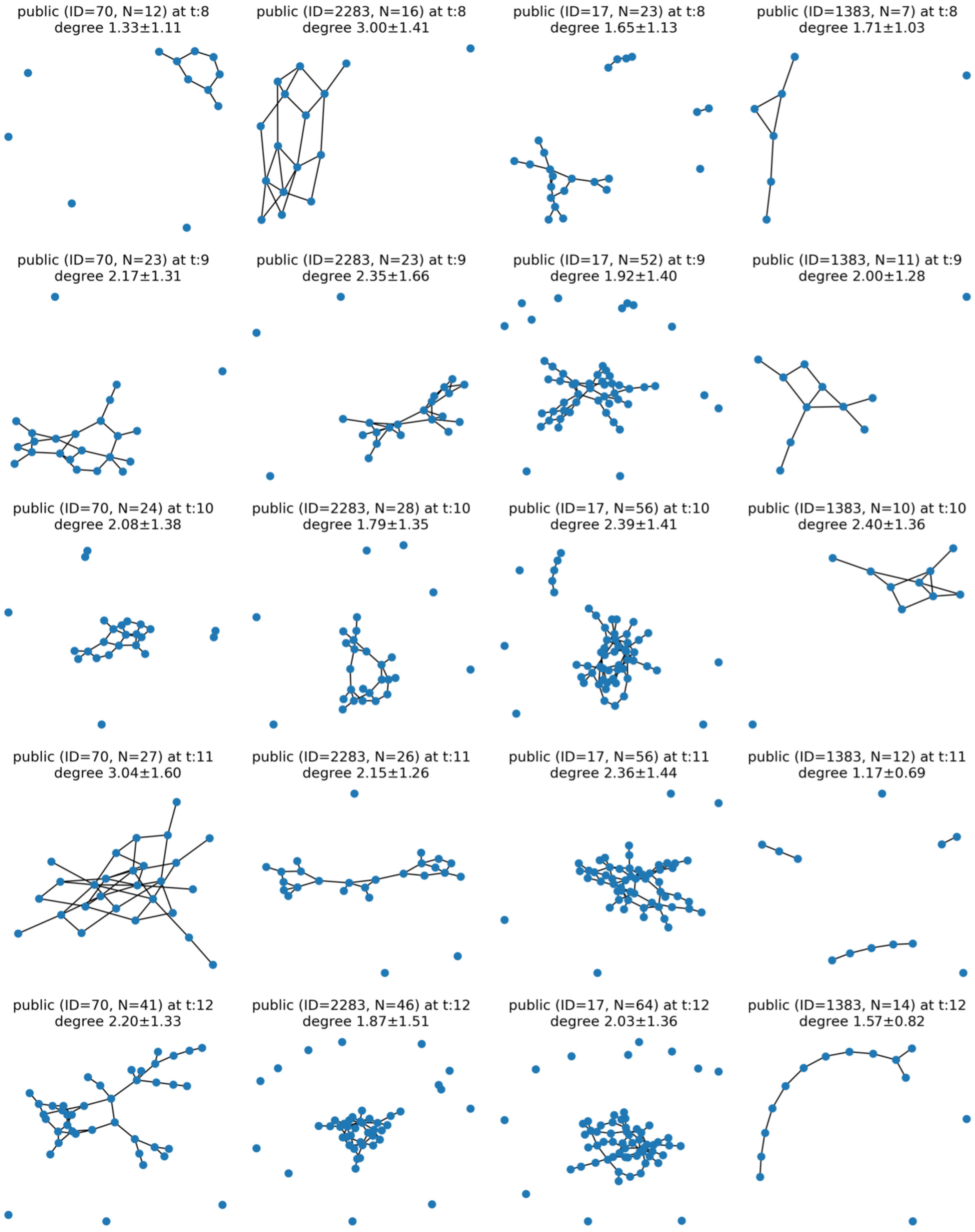

Figure S30: Networks of the contacts at specific times within public places. Exemplary graphs of the interaction networks in public locations, from a baseline simulation of the German community Gangelt (DE). N - number of agents present at the location; the node degree is given as mean +/- std.

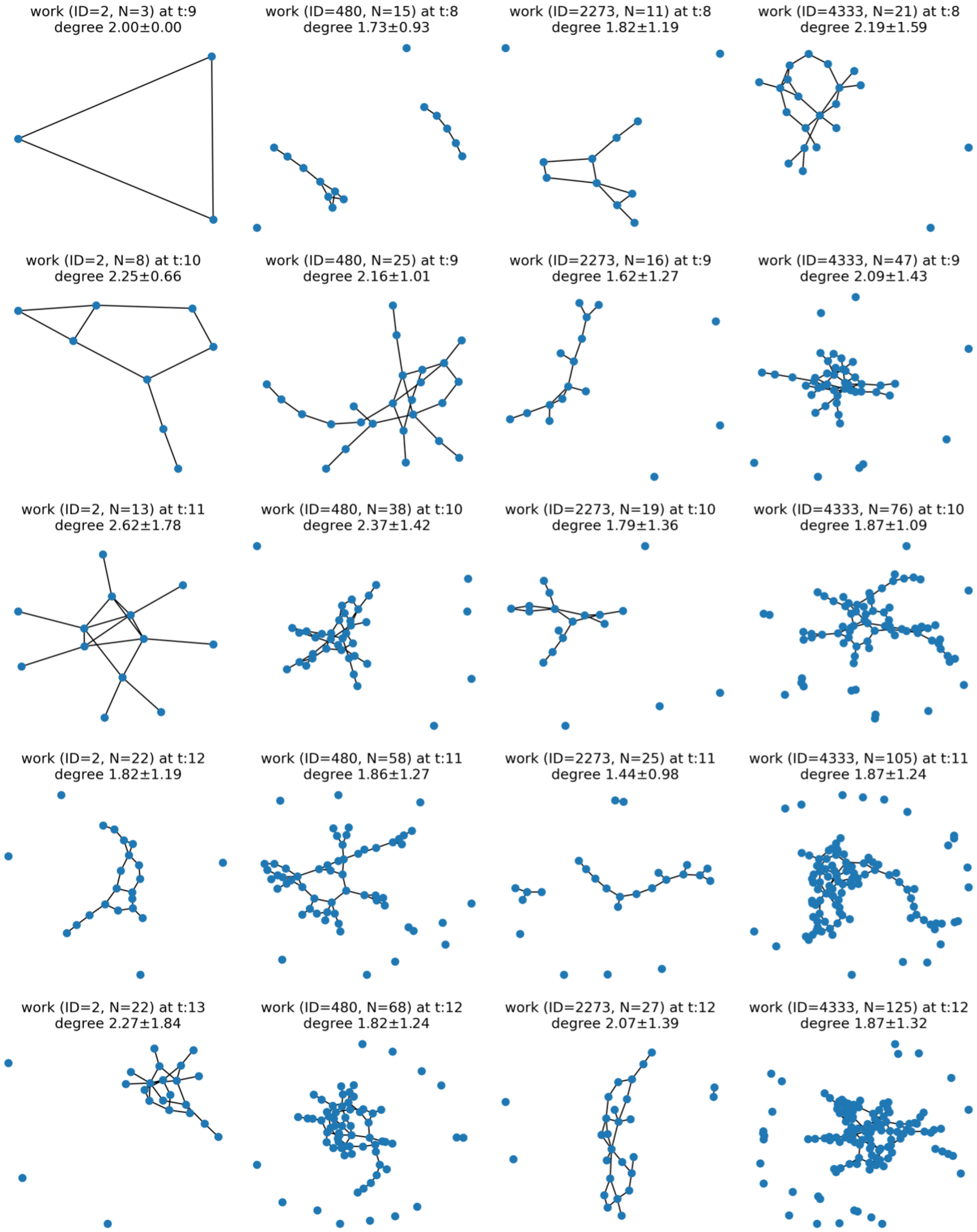

Figure S31: Networks of the contacts at specific times within workplaces. Exemplary graphs of the interaction networks in public locations, from a baseline simulation of the German community Gangelt (DE). N number of agents present at the location; the node degree is given as mean +/- std.

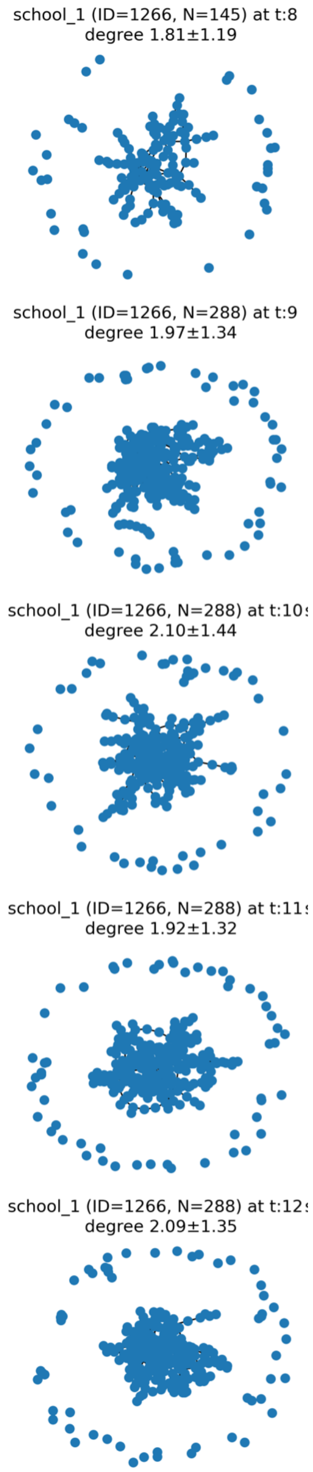

Figure S32: Networks of the contacts at specific times within a school (6-12years). Exemplary graphs of the interaction networks in school locations from a baseline simulation of the German community Gangelt (DE). N - number of agents present at the location; the node degree is given as mean +/- std.

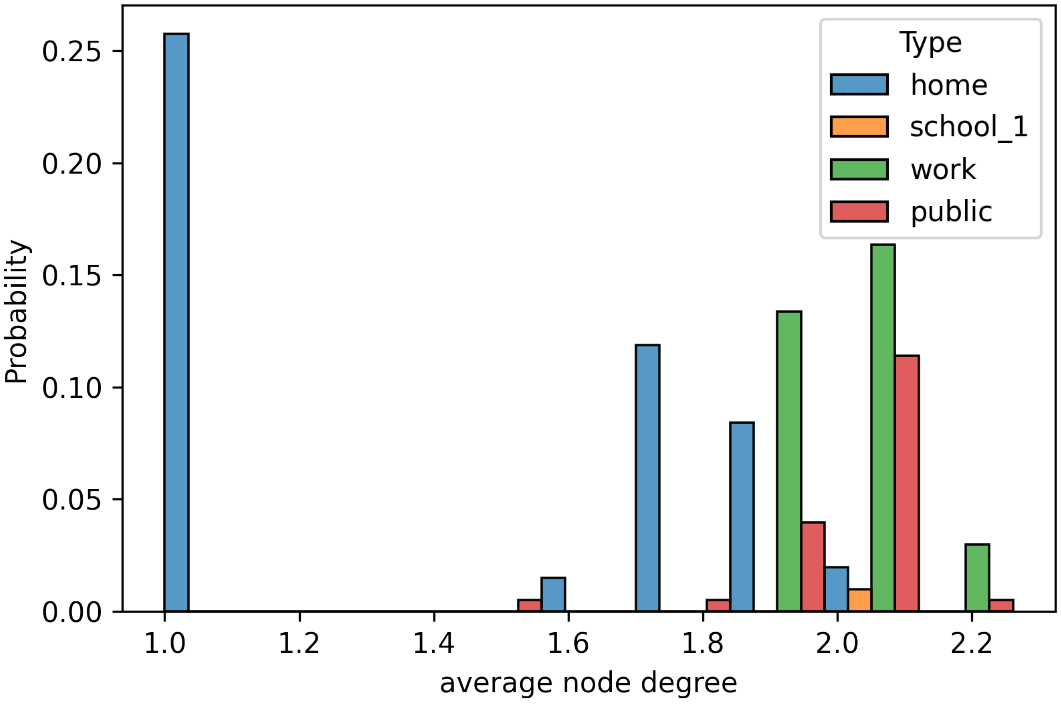

Figure S33: Average node degree of interaction networks during one time step (hour) sorted by location type. Results from a baseline simulation of the German community Gangelt (DE).

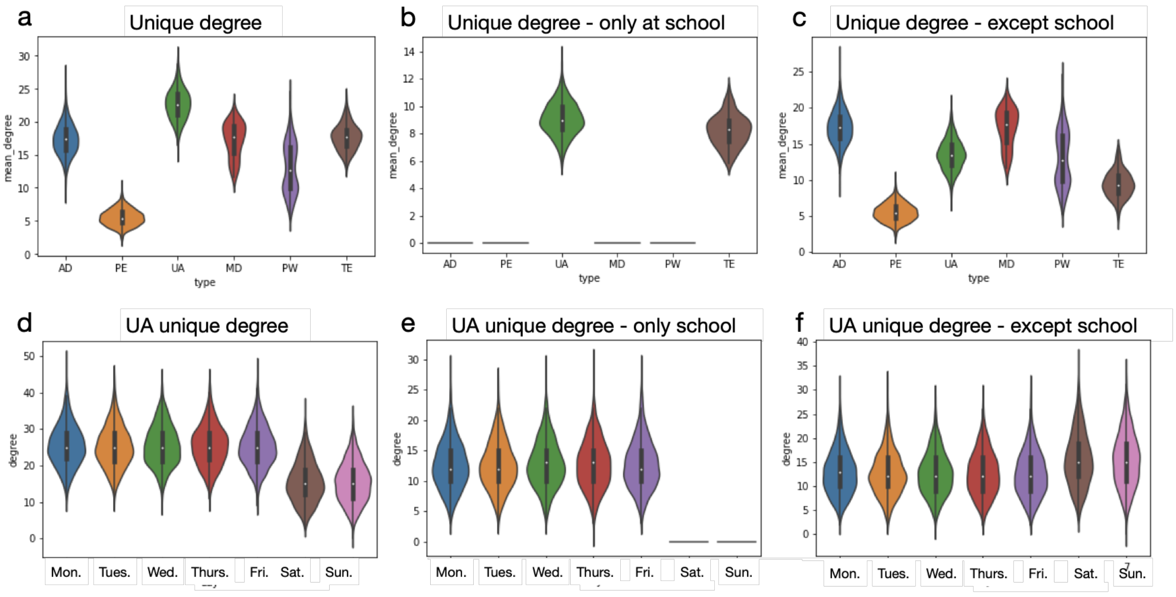

Figure S34: Distribution of average daily node degrees (unique contacts) by archetype and the daily node degree of underaged individuals over the different days of the week. Results from a baseline simulation of the German community Gangelt (DE).

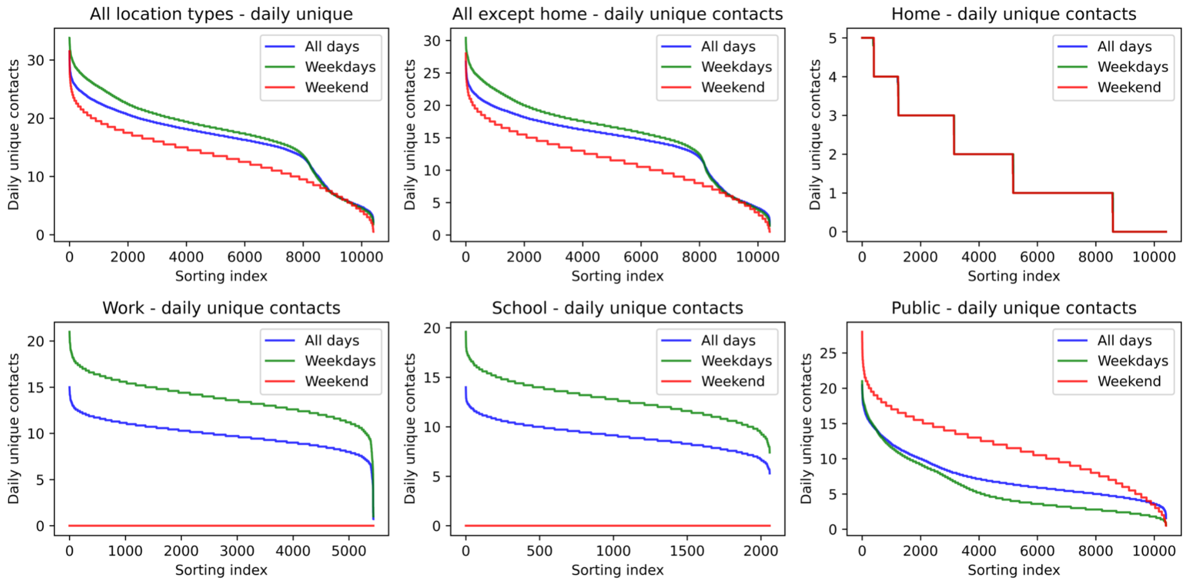

Figure S35: Ranked mean (over weekdays and weekend) of daily degree of contacts at different (combinations of) locations for the municipality of Gangelt.

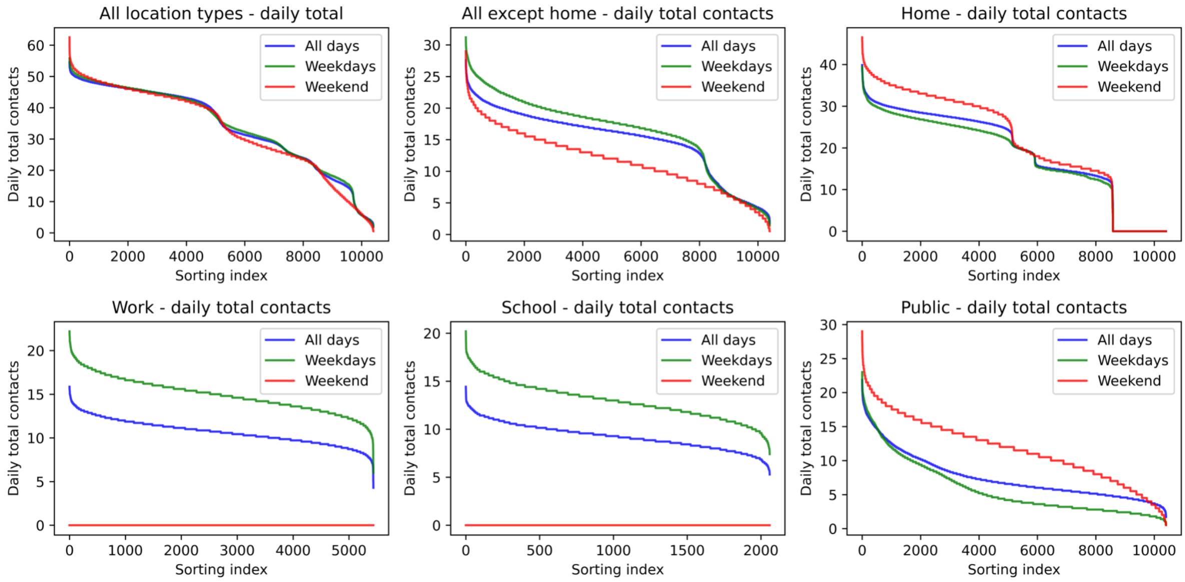

Figure S36: Ranked mean (over weekdays and weekend) of daily number of total contacts at different (combinations of) locations for the municipality of Gangelt.

4.7.2 Infection networks

The infection network is a directed subset of the temporal interaction network, including only those interactions during which the infection was transmitted to a new agent. This enables us

to follow individual infection transmission chains. An example of the emerging network is represented in **Fig. S37**.

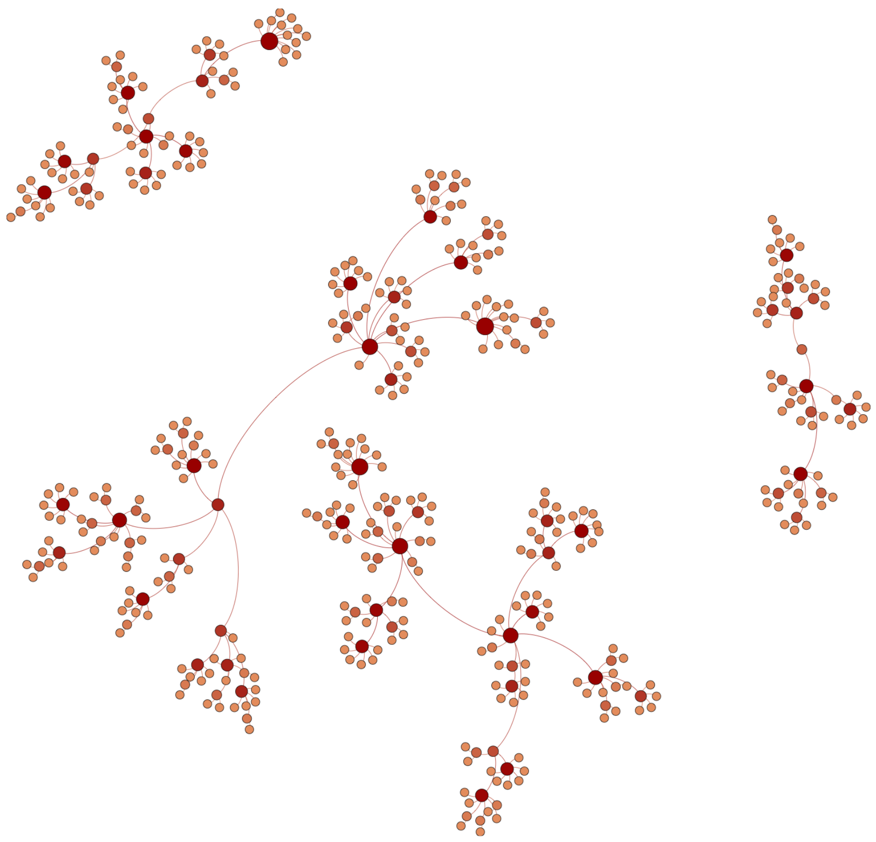

Figure S37: Infection networks in Gangelt unfolding within 300 hours (single simulation). Starting from four infected individuals other individuals get infected creating initially unconnected networks (which may or may not become connected later).

4.7.3 Overrepresentation and underrepresentation of specific groups in the infection process

For example, comparing the distributions of agents that transmit infections and of locations at which infections are transmitted to the respective distributions of initialized agents and locations, provides a possibility to investigate the impact of specific population subgroups and location types on infection spreading. In particular, we analyzed the difference in the contribution to infection spreading between different occupations, as can be seen in the over- and underrepresentation plots (**Fig. S38).** Additionally, we analyzed the average infection caused by an emitter, resolved for its occupation **(Fig. S39**).

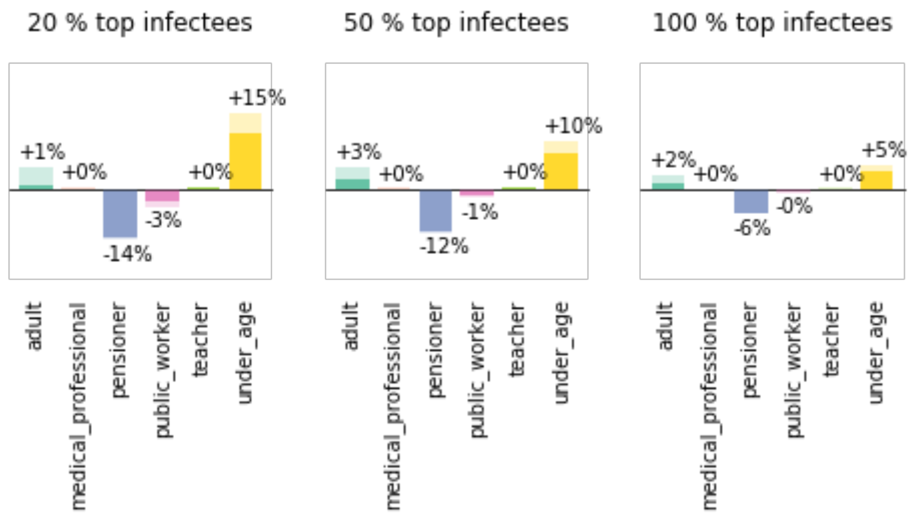

Figure S38: Over and under representation of agents with specific schedule types in the fractions of top spreaders (Gangelt). Difference in the fraction of spreaders with specific schedule type to the fraction of this type in the population.

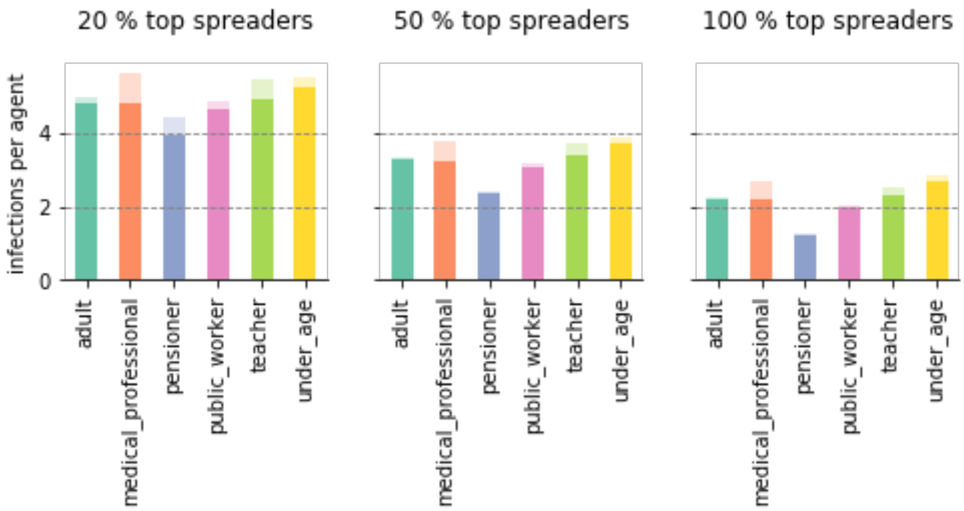

Figure S39: Pattern in schedule type dependent infection rates (Gangelt). Average infections caused by each agent resolved for schedule type and sorted for percentage of top spreaders. Shaded area corresponds to standard deviation of mean.

4.8 Immunization scenarios

We simulated different immunization strategies and a selection of effects and efficacies of vaccinations. In addition, we tested the impact of three SARS-CoV-2 strains, namely wild type (WT), alpha, and delta variants, on accomplishing population immunity. Vaccinated agents obtain new probabilities either for a single state transition or for multiple state transitions depending on the effects and efficacies assumed. This strategy allows us to account for different vaccines as well as to consider different strains. For better comparison of the scenarios, all simulations are based on the same modeled world based on real-world data for Gangelt, Germany. Thus, the same locations, the same agents and the same daily routines are given in all vaccination scenarios.

4.8.1 Vaccination strategies

Prior to the simulations we defined sorted lists of individuals, reflecting different immunization strategies. The simulations were initialized with different fractions of vaccinated agents. We scanned fractions of vaccinated individuals ranging from 0.1 to 0.99. All ratios were repeatedly simulated for 3000 hours 96 times (N=96) with four initial infection emitters chosen from the remaining pool of non-vaccinated individuals. All simulations were analyzed with respect to outbreak frequency (ratio between simulations with $R_{eff}(0)>1$ and all simulations), attack rate, fractions of individuals admissioned to hospital or to ICU, and deceased relative to all individuals, to vaccinated individuals, and to non-vaccinated individuals

Sorting algorithms for the analyzed immunization strategies:

- **forecasted**
  Before carrying out the simulations with a given percentage of individuals set to immunized, pre-simulations have been performed to forecast potentially infected individuals. The lists are based on 96 complete infection chains from 96 preceding simulations starting always with the same initial infection emitters. Resulting infection receivers were sorted by infection time and the precise given percentage was set to R.

- **random**
  96 different lists of individuals were shuffled randomly and the given percentage set to R.

- **age**
  One list of all inhabitants of the community was used, sorted by decreasing age of the individuals.

- **interactions**
  One list was used, sorted by decreasing mean absolute contact numbers, again resulting from 96 preceding simulations, but here without infection spreading.

- **overrepresentation**
  One list was used, sorted by occupations, starting with occupations showing the strongest overrepresentation in infection (**Fig. S38**) to occupations with the least overrepresentation in infection: underage (UA), adult (AD), teacher (TE), medical personnel (MP), public worker (PW), pensioners (PE).

- **combined**
  One list of three consecutive blocks was used. The first block contained all individuals above age 60 with decreasing age (as in list "age"). The second block contained all individuals from age 13 to age 59 sorted for mean absolute contacts as in "interactions". The third block contained all individuals below age 12 with decreasing age.

- **household**
  First, one person per household gets vaccinated (order descending age), then a second person and so forth.

It is important to note that the performance of the different strategies depends on the infectivity k_I, i.e. the strain. While the general pattern between the different strategies is preserved, the drop in the outbreak frequency is shifted toward higher fractions of vaccinated (**Fig. S42, Fig. S46, Fig. S50**). Further, we analyzed the development of the AR and the ratios for hospitalization, ICU admission and death (**Fig. S41, Fig. S45 and Fig. S49**), the resulting VE (**Fig. S40, Fig. S44 and Fig. S48**) and the effective reproduction number (**Fig. S43, Fig. S47 and Fig. S51**) for all strategies and the three considered strains. All simulations are based on the German municipality Gangelt with 10.351 agents.

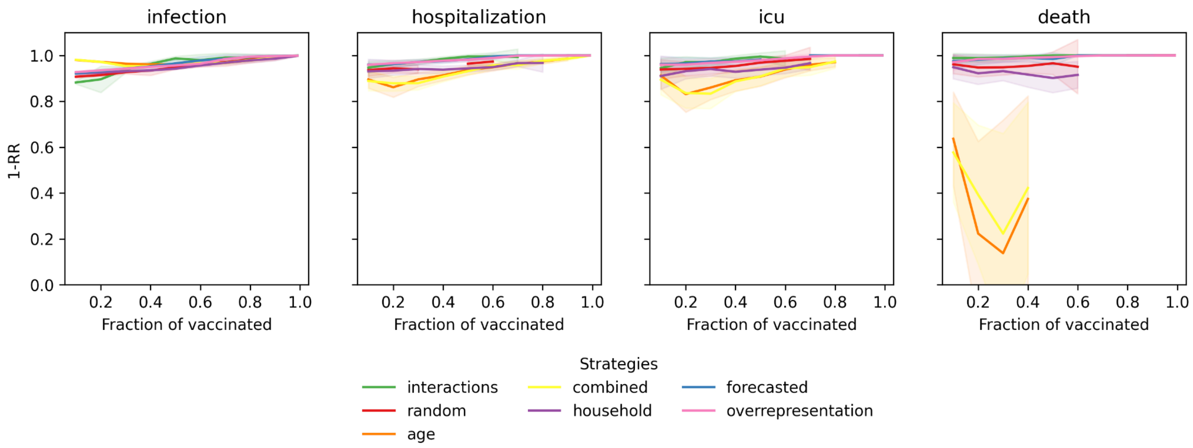

Figure S40: Vaccination fraction scans for different vaccination strategies (WT, Gangelt). Theoretical vaccine efficacy as reduction in risk ratio (1-RR) against infection, hospital admission, ICU admission, and death for different vaccination strategies using reduction of infection probability and severity of COVID-19 progression (RIP+S - 97.5% infection reduction + 20% severity reduction, compare **subsection 4.8.3**).

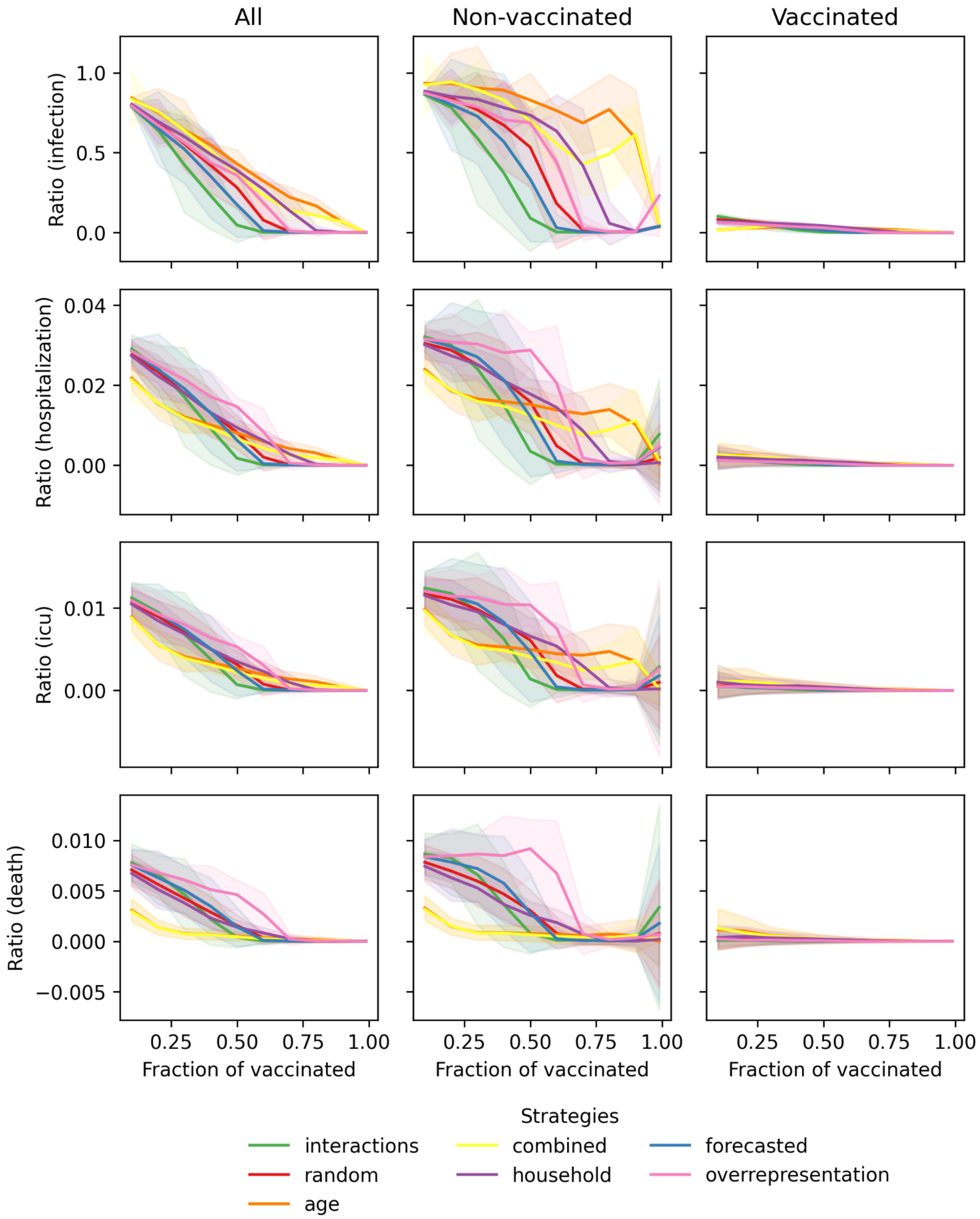

Figure S41: Impact of vaccination strategies on agent health states (WT, Gangelt). Ratios of agents in specific states to population sub-groups (all, non-vaccinate, vaccinated) for different fractions of vaccinated individuals using RIP+S (97.5% infection reduction + 20% severity reduction, compare **subsection 4.8.3**).

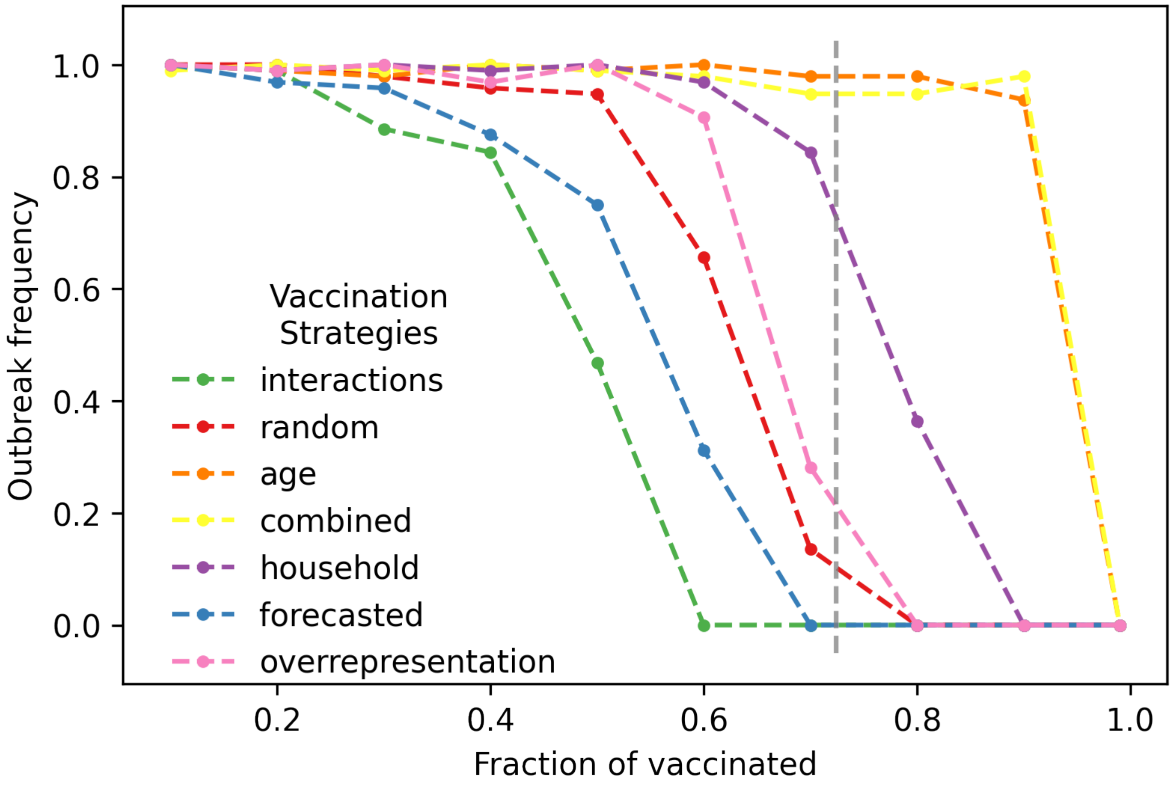

Figure S42: Impact of vaccination strategies on outbreak frequency (WT, Gangelt). Share of simulations with $R_{eff}(0)>1$, i.e. showing an outbreak, for different fractions of vaccinated individuals using RIP+S (97.5% infection reduction + 20% severity reduction, compare **subsection 4.8.3**). Grey dotted line represents the theoretical population immunity threshold for R_0=2.83 and VE=0.9.

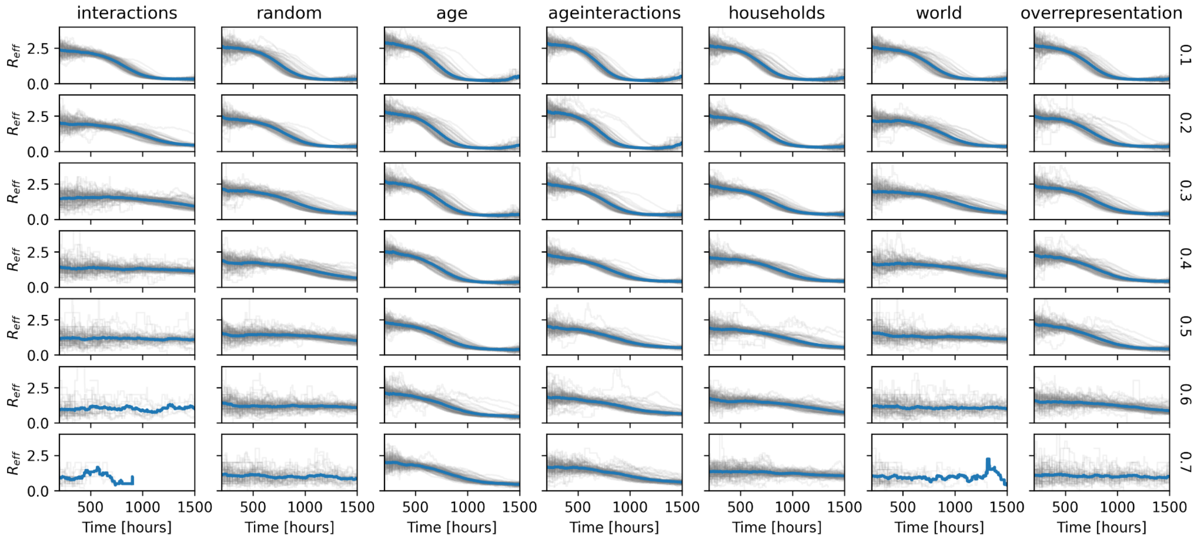

Figure S43: Vaccination strategy impact on R_{eff} (WT, Gangelt). Reproduction number R_{eff} over time for different strategies and fractions of vaccinated individuals (right y-axis) using RIP+S (97.5% infection reduction + 20% severity reduction, compare **subsection 4.8.3**).

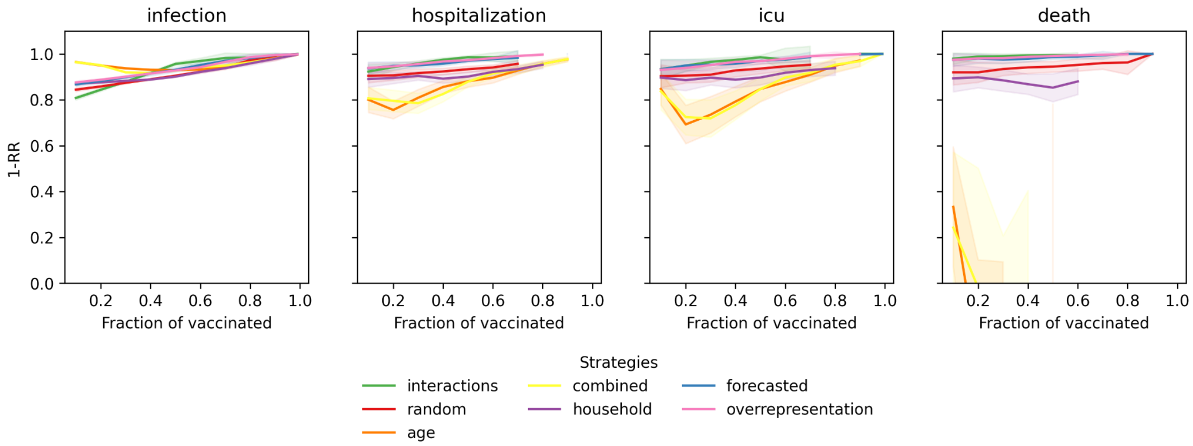

Figure S44: Vaccination fraction scans for different vaccination strategies (Alpha strain, Gangelt). Theoretical vaccine efficacy as reduction in risk ratio (1-RR) against infection, hospital admission, ICU admission, and death for different vaccination strategies using reduction of infection probability (RIP+S - 97.5% infection reduction + 20% severity reduction, compare **subsection 4.8.3**).

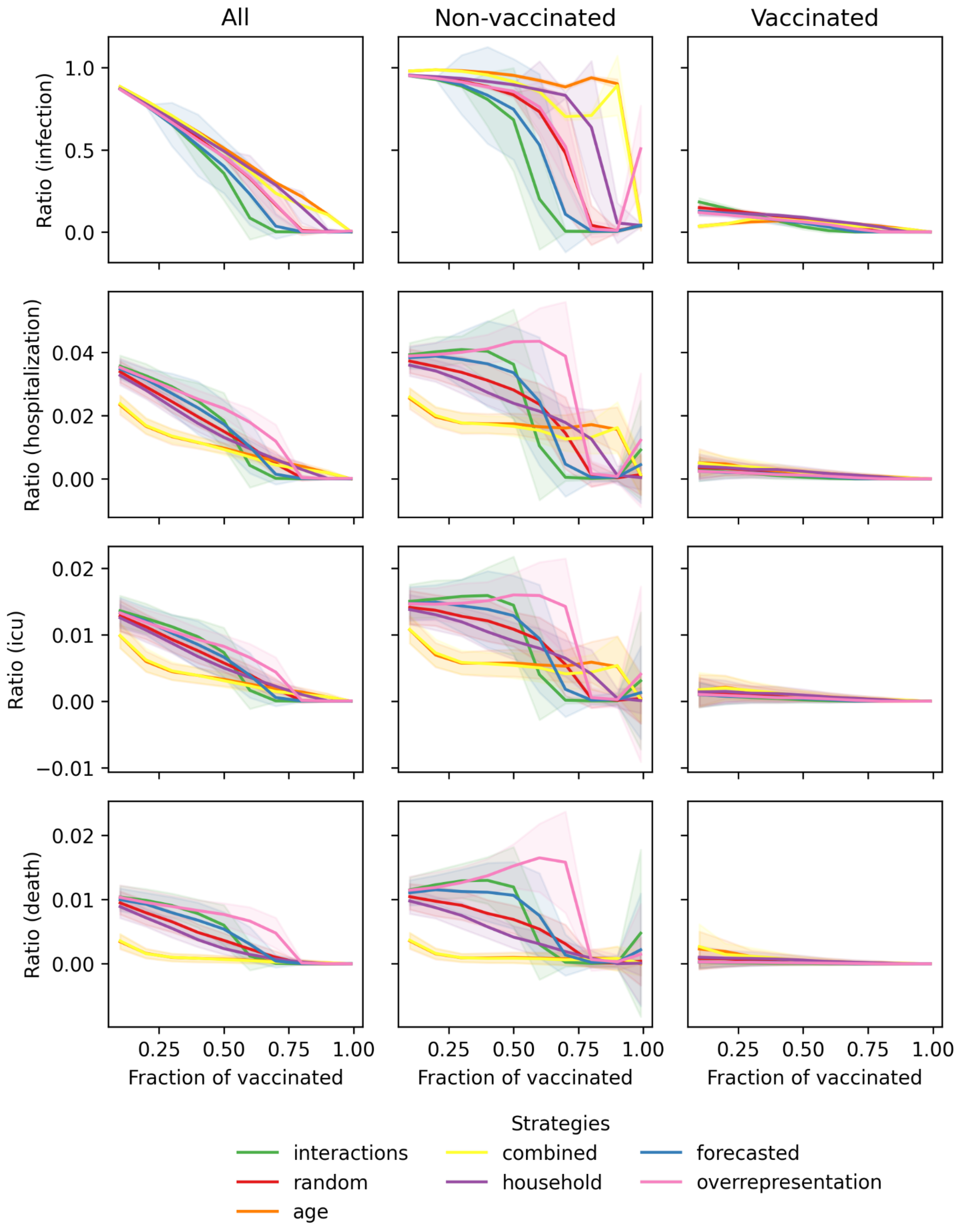

Figure S45: Impact of vaccination strategies on agent health states (Alpha strain, Gangelt). Ratios of agents in specific states to population sub-groups (all, non-vaccinate, vaccinated) for different fractions of vaccinated individuals using RIP+S (97.5% infection reduction + 20% severity reduction, compare **subsection 4.8.3**).

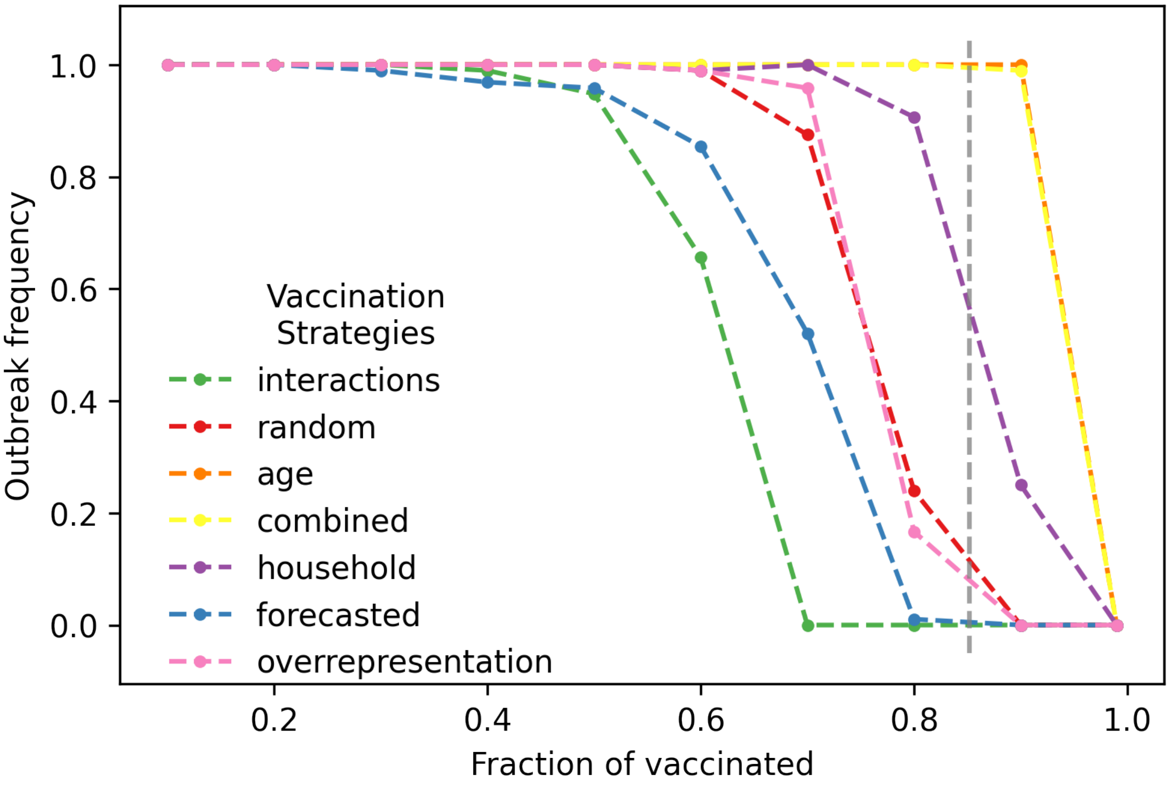

Figure S46: Impact of vaccination strategies on outbreak frequency (Alpha strain, Gangelt). Share of simulations with $R_{eff}(0)>1$, i.e. showing an outbreak, for different fractions of vaccinated individuals using RIP+S (97.5% infection reduction + 20% severity reduction, compare **subsection 4.8.3**). Grey dotted line represents the theoretical population immunity threshold for R_0=4.28 and VE = 0.9.

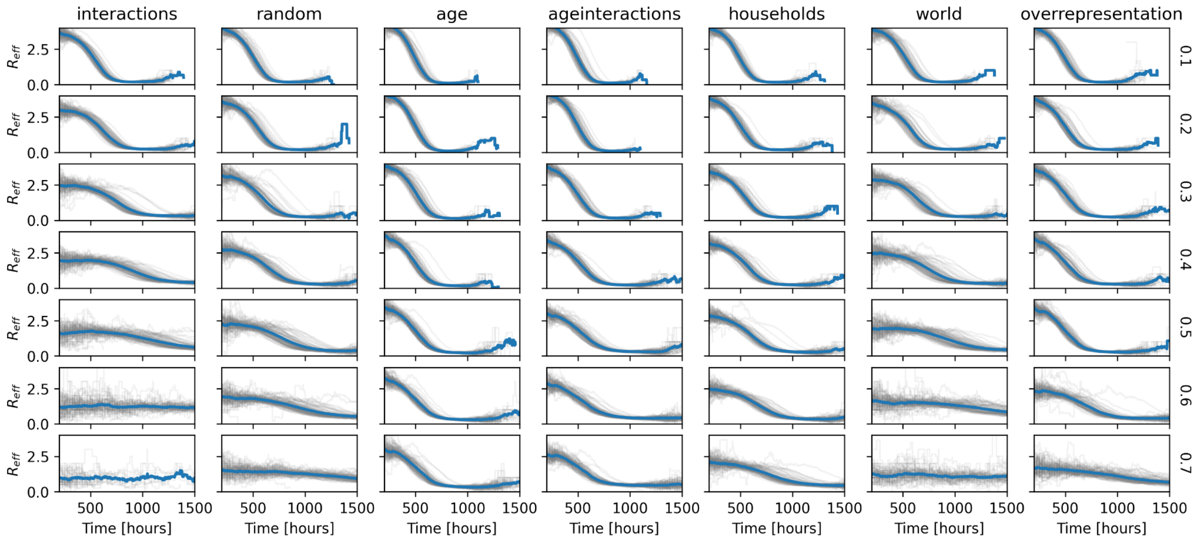

Figure S47: Vaccination strategy impact on R_{eff} (Alpha strain, Gangelt). Reproduction number R_{eff} over time for different strategies and fractions of vaccinated individuals (right y-axis) using RIP+S (97.5% infection reduction + 20% severity reduction, compare **subsection 4.8.3**).

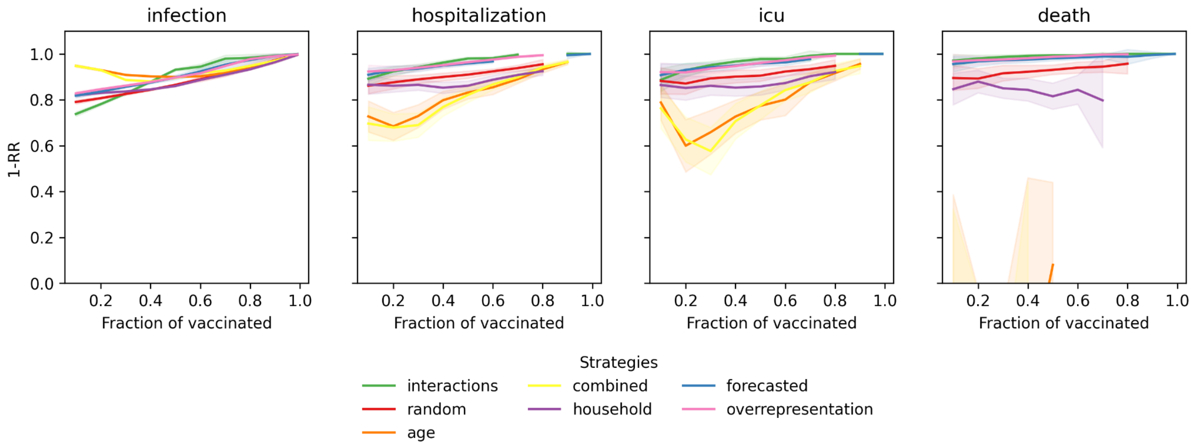

Figure S48: Vaccination fraction scans for different vaccination strategies (Delta strain, Gangelt). Theoretical vaccine efficacy as reduction in risk ratio (1-RR) against infection, hospital admission, ICU admission, and death for different vaccination strategies using reduction of infection probability (RIP+S, 97.5% infection reduction + 20% severity reduction, compare **subsection 4.8.3**).

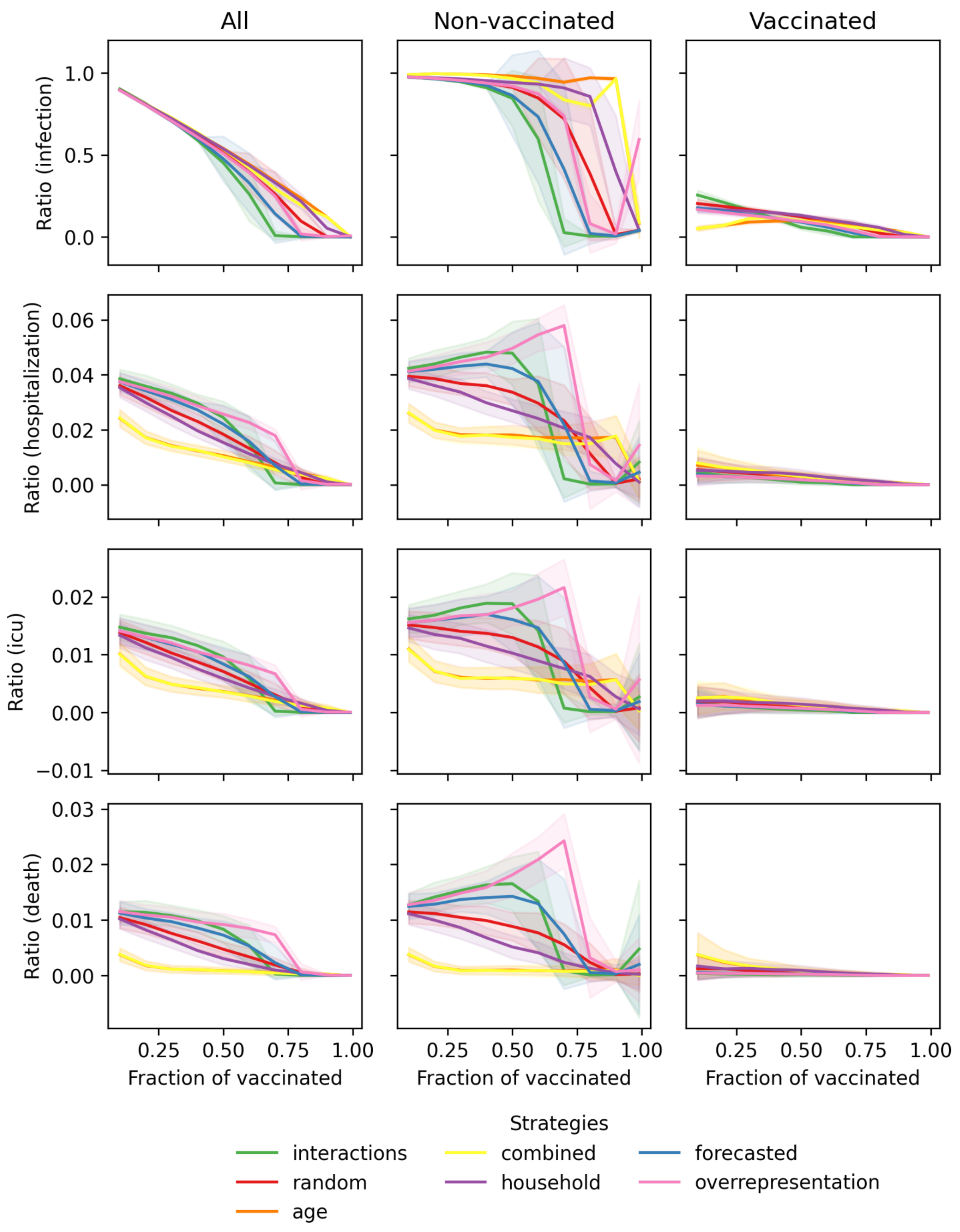

Figure S49: Impact of vaccination strategies on agent health states (Delta strain, Gangelt). Ratios of agents in specific states to population sub-groups (all, non-vaccinate, vaccinated) for different fractions of vaccinated individuals using RIP+S (97.5% infection reduction + 20% severity reduction, compare **subsection 4.8.3**).

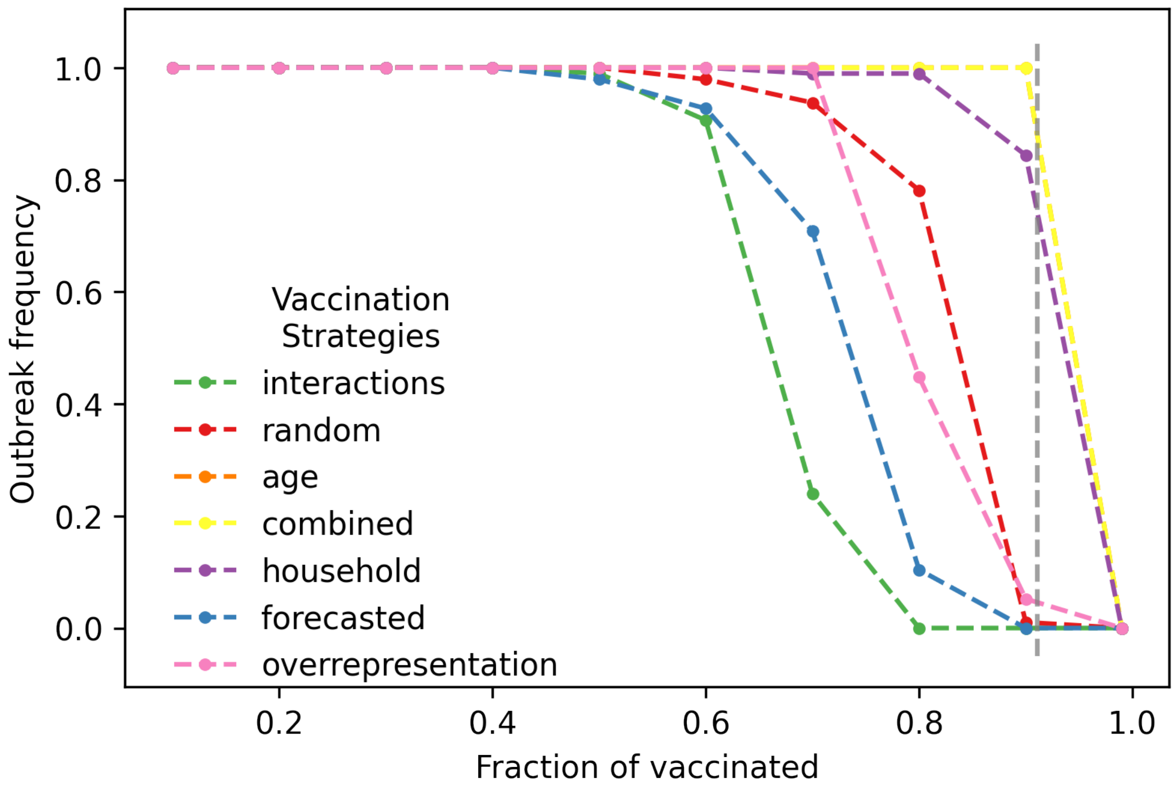

Figure S50: Impact of vaccination strategies on outbreak frequency (Delta strain, Gangelt). Share of simulations with $R_{eff}(0)>1$, i.e. showing an outbreak, for different fractions of vaccinated individuals using RIP+S (97.5% infection reduction + 20% severity reduction, compare **subsection 4.8.3**). Grey dotted line represents the theoretical population immunity threshold for $R_0=5.58$ and VE=0.9.

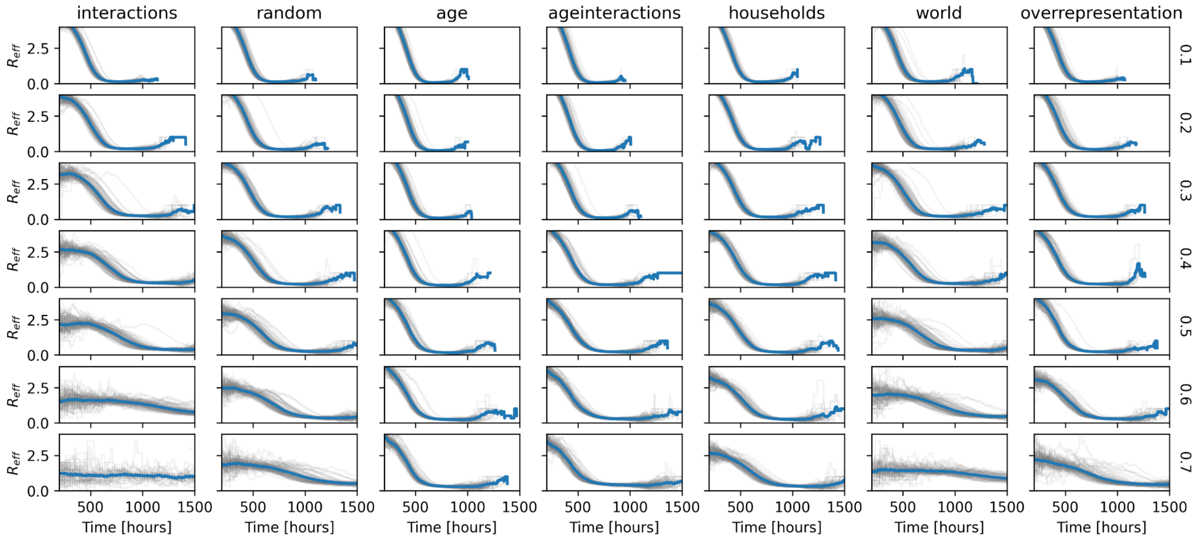

Figure S51: Vaccination strategy impact on R_{eff} (Delta strain, Gangelt). Reproduction number R_{eff} over time for different strategies and fractions of vaccinated individuals (right y-axis) using RIP+S (97.5% infection reduction + 20% severity reduction, compare **subsection 4.8.3**).

4.8.2 Impact of different strains on vaccination scenarios

We used different infection transmission probabilities to reflect different SARS-CoV-2 strains. In particular, we used the wild type (WT) strain (infectivity = 0.14), alpha strain (infectivity = 0.24), and delta strain (infectivity = 0.34). We checked the effect of the different strains on the risk reduction for infection and disease progression after vaccination (**Fig. S52**). Also, we compared vaccinated and non-vaccinated subgroups to the entire population (**Fig. S53**). As expected, the higher the infectivity, i.e. the more aggressive the strain, the higher the probability of new outbreaks even for highly vaccinated fractions.

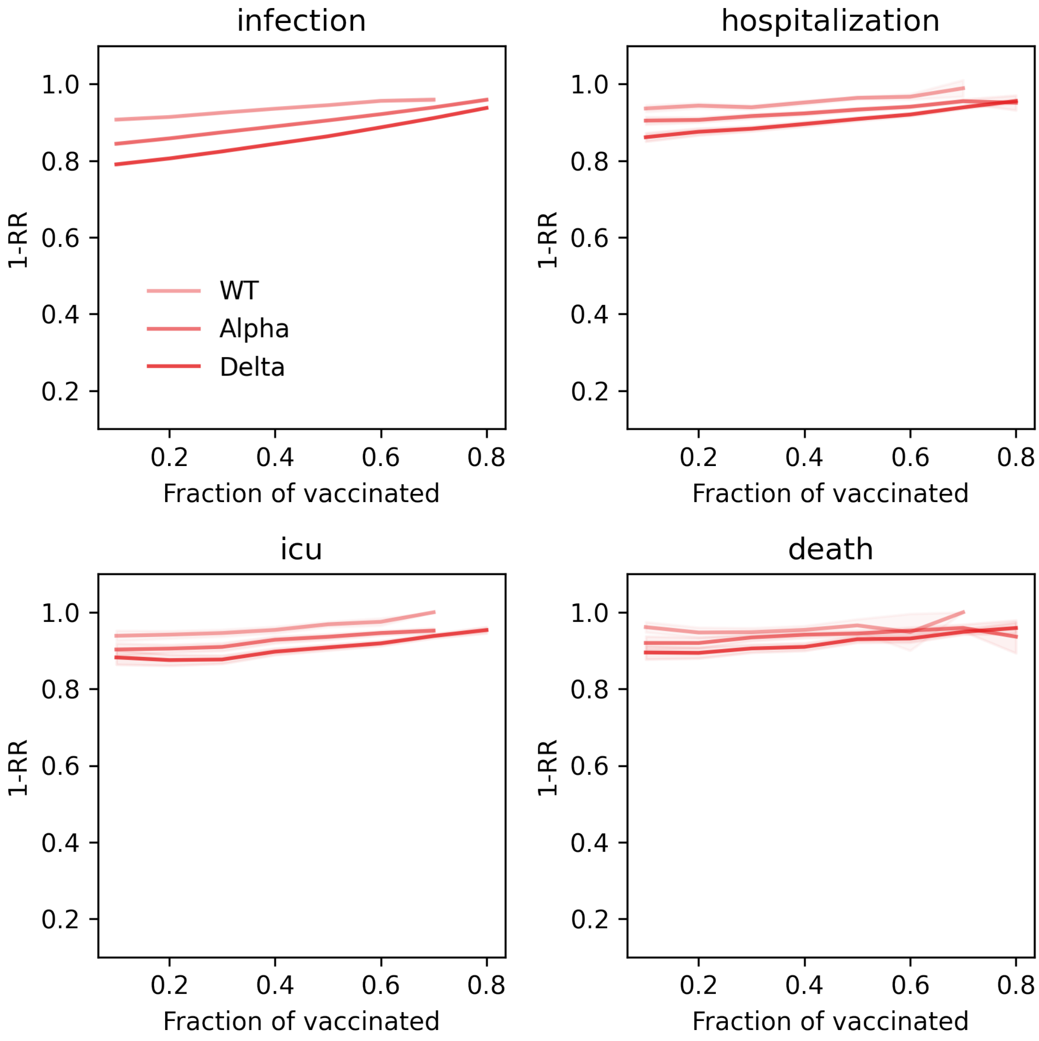

Figure S52: Vaccination fraction scans for different SARS-CoV-2 strains (random, GangeIt). Theoretical vaccine efficacy as reduction in risk ratio (1-RR) against infection, hospital admission, ICU admission, and death for different infection transmission probabilities values using RIP. Infection transmission probabilities represent the strains wild type (WT), alpha, and delta; random vaccination strategy has been applied.

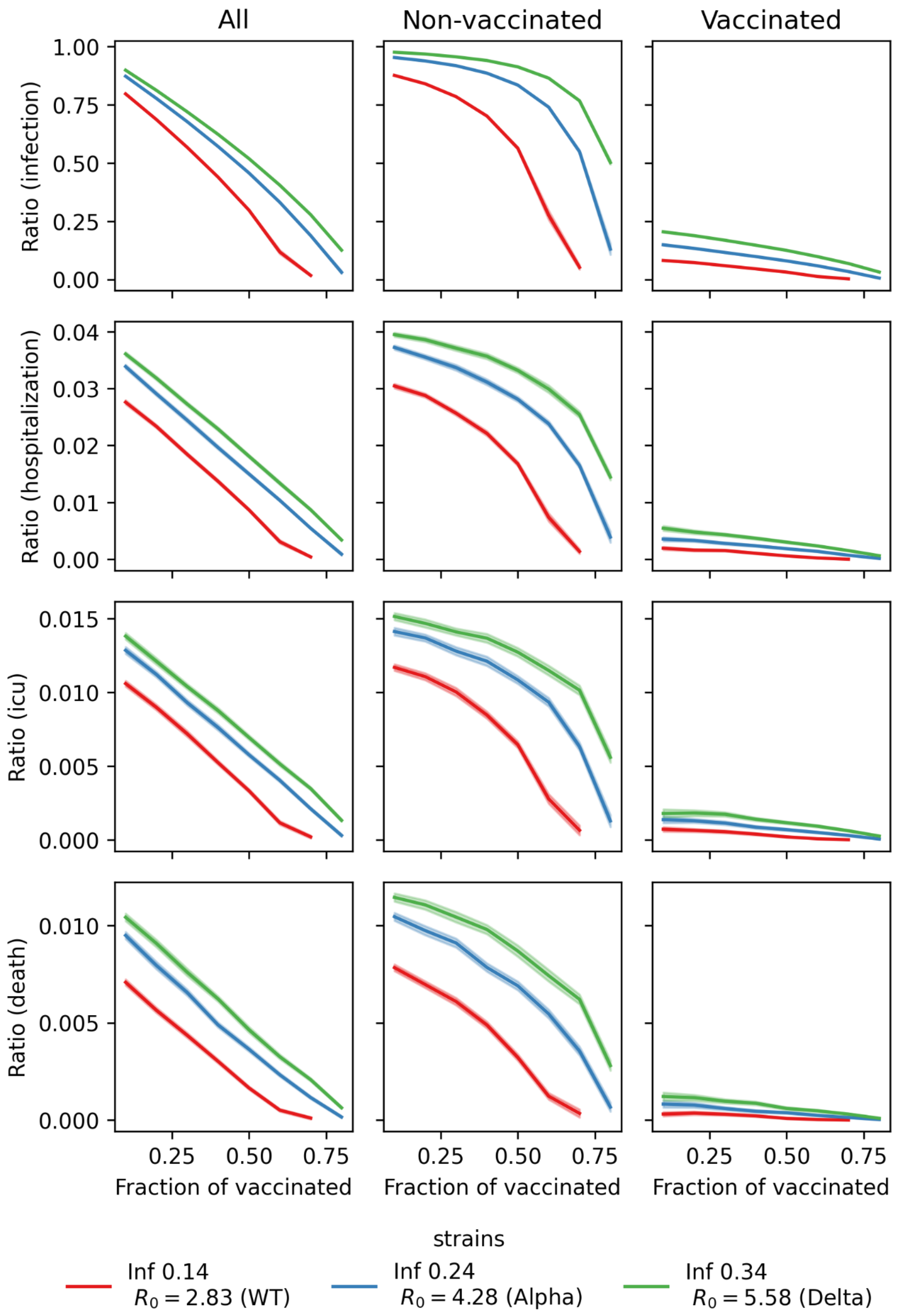

Figure S53: Impact of strains on agent health states (random, GangeIt). Ratios of agents in specific states to population (sub-groups) for different fractions of vaccinated individuals. WT, alpha, and delta strains are analyzed; random vaccination strategy has been applied.

4.8.3 Vaccination effects

We defined four scenarios to test different vaccination models:

- **Reduction of all probabilities (RAP)**
  Reduction of all state transition probabilities except recovery probabilities (but including infection probability) by 90% for vaccinated agents to represent vaccination effects on infection with SARS-CoV-2 and all stages of COVID-19 progression.

- **90% success (90S)**
  90% of vaccinated agents get completely immunized (infection probability is reduced by 100%), while for 10% vaccination has no effect.

- **Reduction of infection probability (RIP)**
  Infection probability of vaccinated agents gets reduced by 90% to represent an incomplete immunization.

- **RIP and severeness (RIP+S)**
  Infection probability of vaccinated agents gets reduced by 90% and hospitalization probability gets reduced by 20% to represent less severe COVID-19 progressions.

We analyzed the impact of the different vaccination models on the risk reduction for infection and disease progression (**Fig. S54**). Furthermore, we compared vaccinated and non-vaccinated subgroups to the entire population (**Fig. S55**). Interestingly, the tested vaccine mechanisms did not differ with respect to the outbreak frequency (**Fig. S56**).

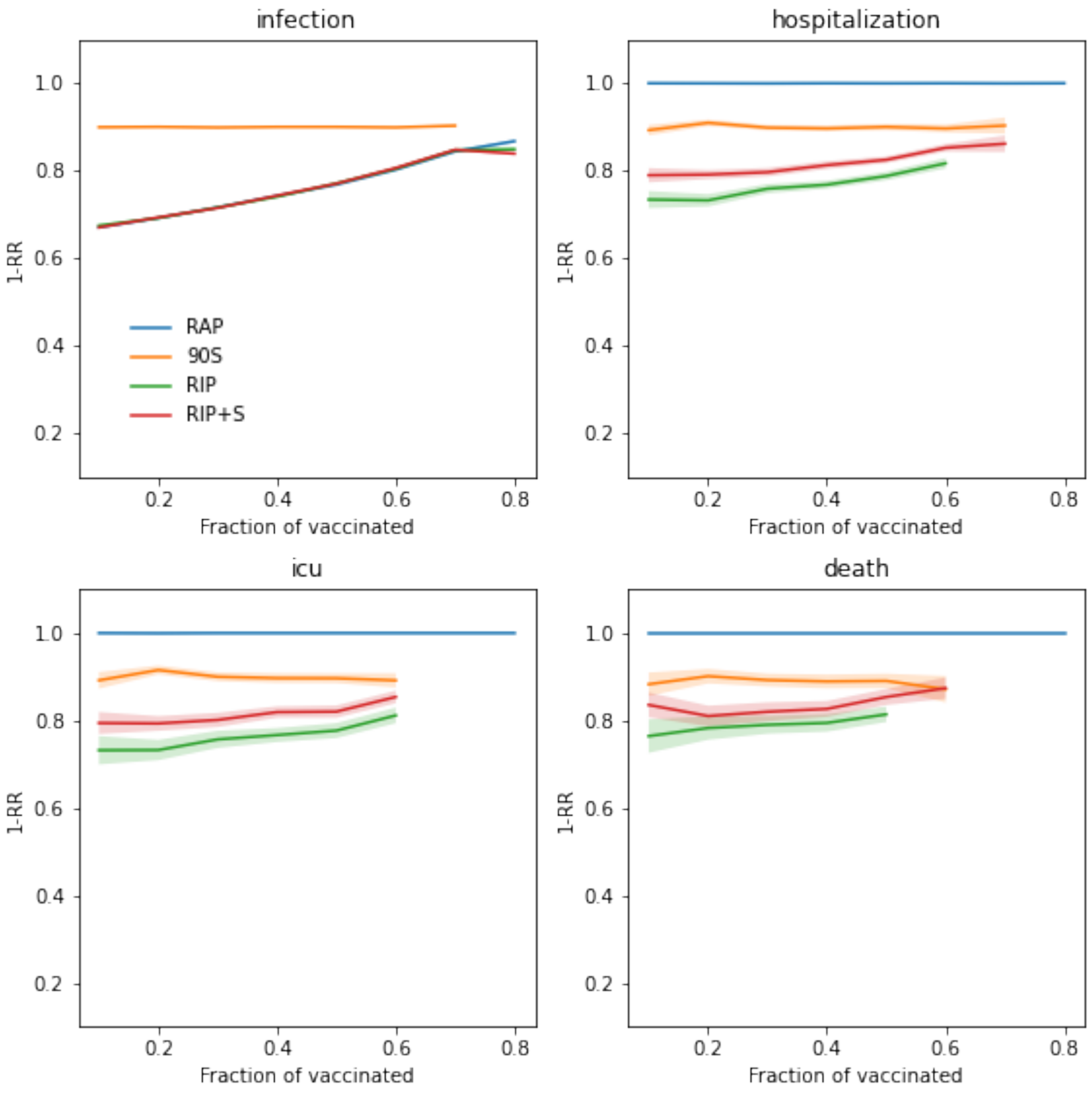

Figure S54: Vaccination model comparison (WT, Gangelt). Theoretical vaccine efficacy as reduction in risk ratio (1-RR) against infection, hospital admission, ICU admission, and death for different fractions of vaccinated individuals comparing the four tested vaccination models RAP, 90S, RIP, and RIP+S; random vaccination strategy has been applied.

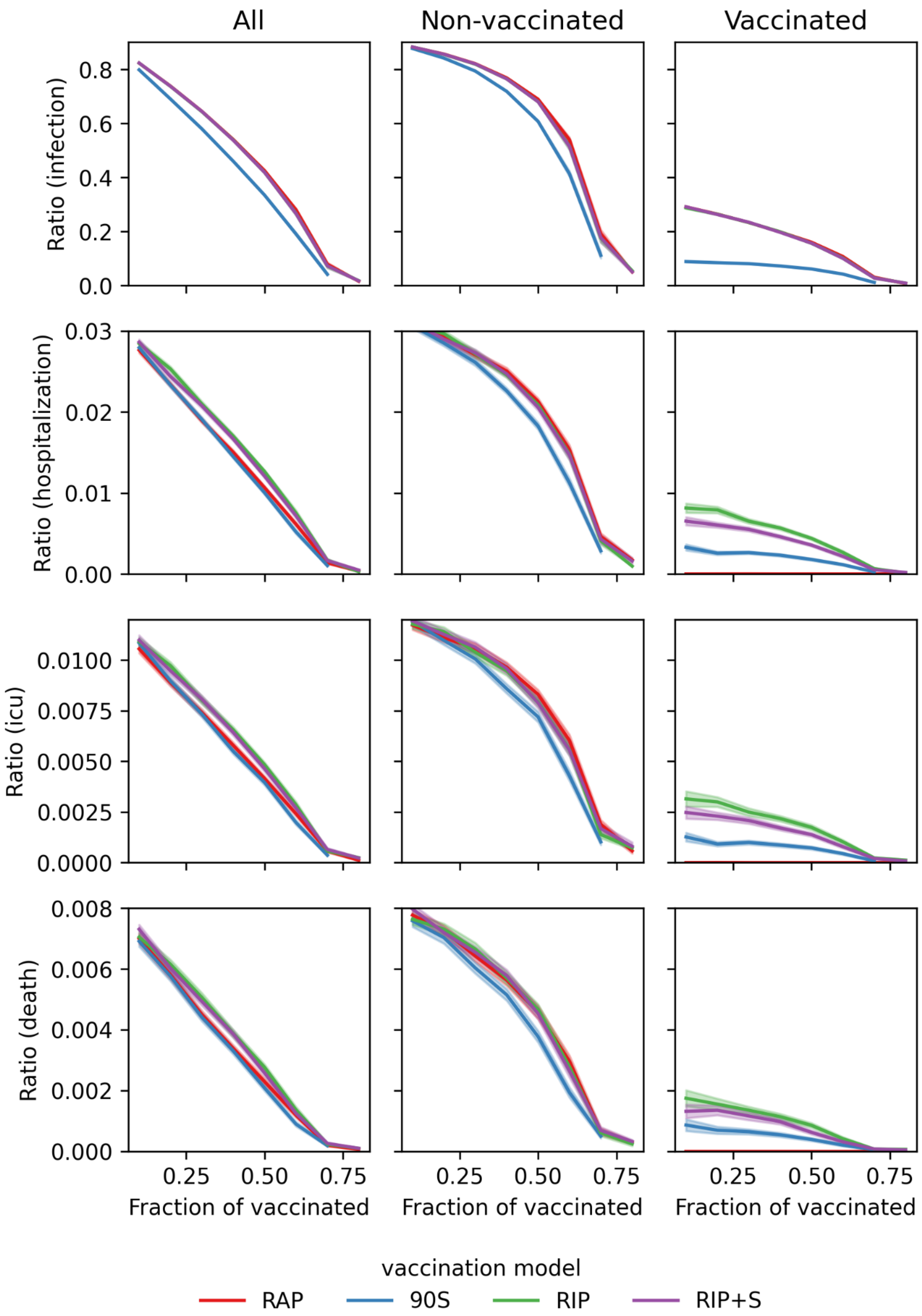

All
Non-vaccinated
Vaccinated
Ratio (infection)
Ratio (hospitalization)
Ratio (icu)
Ratio (death)
0.0
0.2
0.4
0.6
0.8
0.00
0.01
0.02
0.03
0.0000
0.0025
0.0050
0.0075
0.0100
0.000
0.002
0.004
0.006
0.008
0.25
0.50
0.75
Fraction of vaccinated
vaccination model
RAP
90S
RIP
RIP+S

Figure S55: Impact of vaccination models on agent health states (WT, Gangelt). Ratios of agents in specific states to population (sub-groups) for different fractions of vaccinated individuals; random vaccination strategy has been applied.

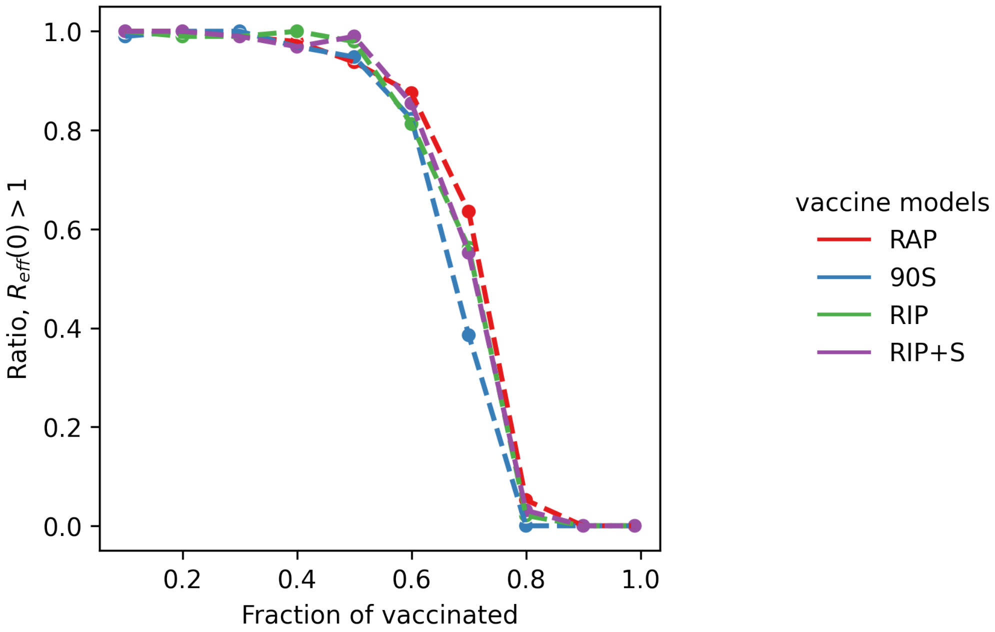

Figure S56: Impact of vaccination models on outbreak frequency (WT, Gangelt). Fraction of simulations with Reff(0)>1, i.e. showing an outbreak, for different fractions of vaccinated individuals.

4.8.4 Vaccine efficacies

To simulate different vaccines, we compared four vaccine efficacies (VE) which reduce infection probability between 90% and 97.5%. For low fractions of vaccinated individuals the VE against infection was 0.75 and 0.91, at 0.925 and .975 reduction infection probability (IP), which is comparable to the reported values for AstraZeneca vaccine (74.5 against alpha variant, Bernal et al. 2021) and for Pfizer/Biontech vaccine (93.7 against alpha variant, Bernal et al. 2021) (**Fig. S57, infection panel**). The reported values for vaccine efficacy of the Biontech vaccine against infection 92% and hospital admission 87% (Dagan et al. 2021) are reflected by 97.5% reduction of infection transmission probability (**Fig. S57, hospitalization and icu panels**).

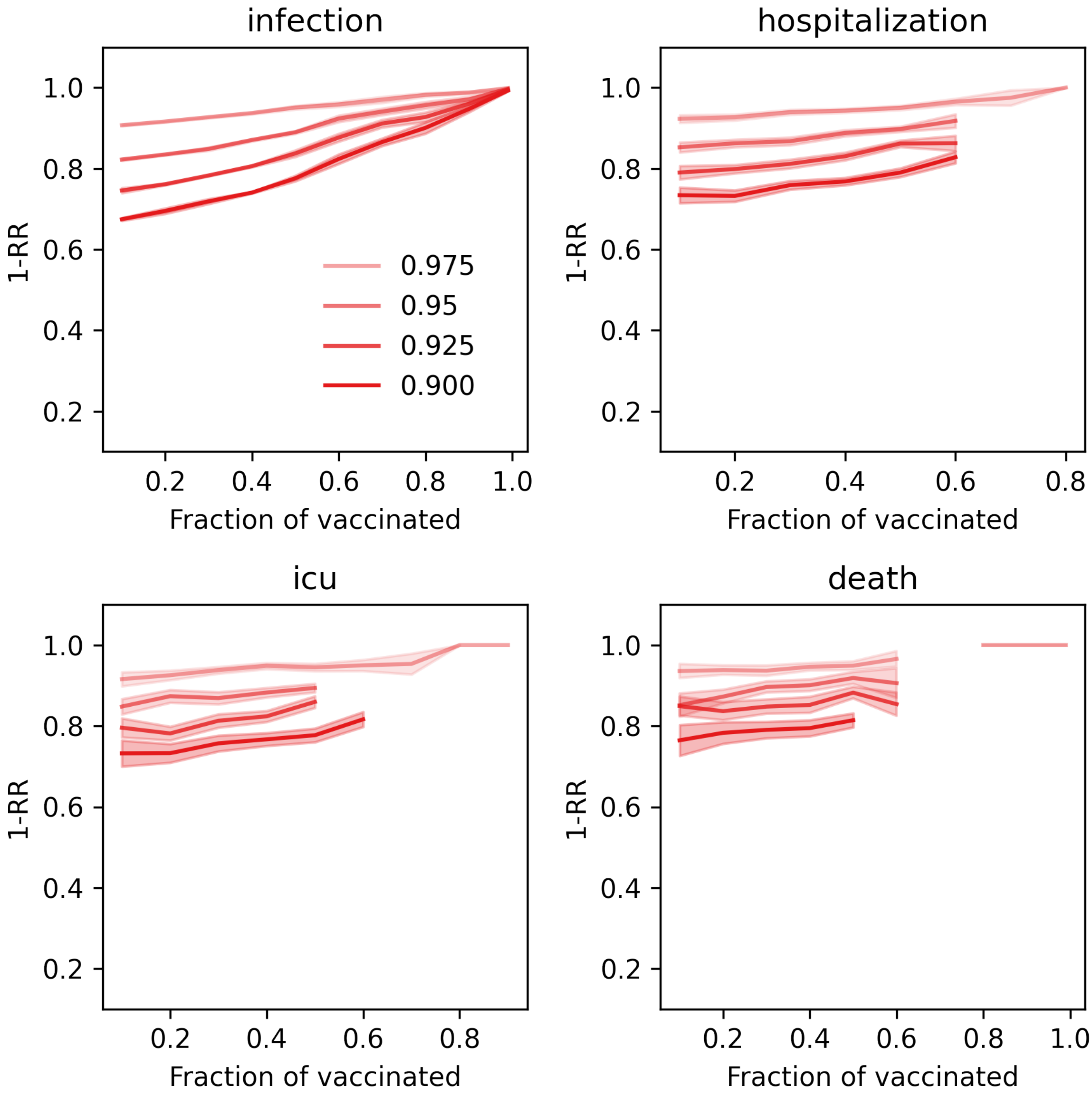

Figure S57: Comparison of different vaccines (WT strain, Gangelt). Theoretical vaccine efficacy as reduction in risk ratio (1-RR) against infection, hospital admission, ICU admission, and death for different fractions of vaccinated using RIP. Shaded area represents CI(95%) for N=96 simulations.

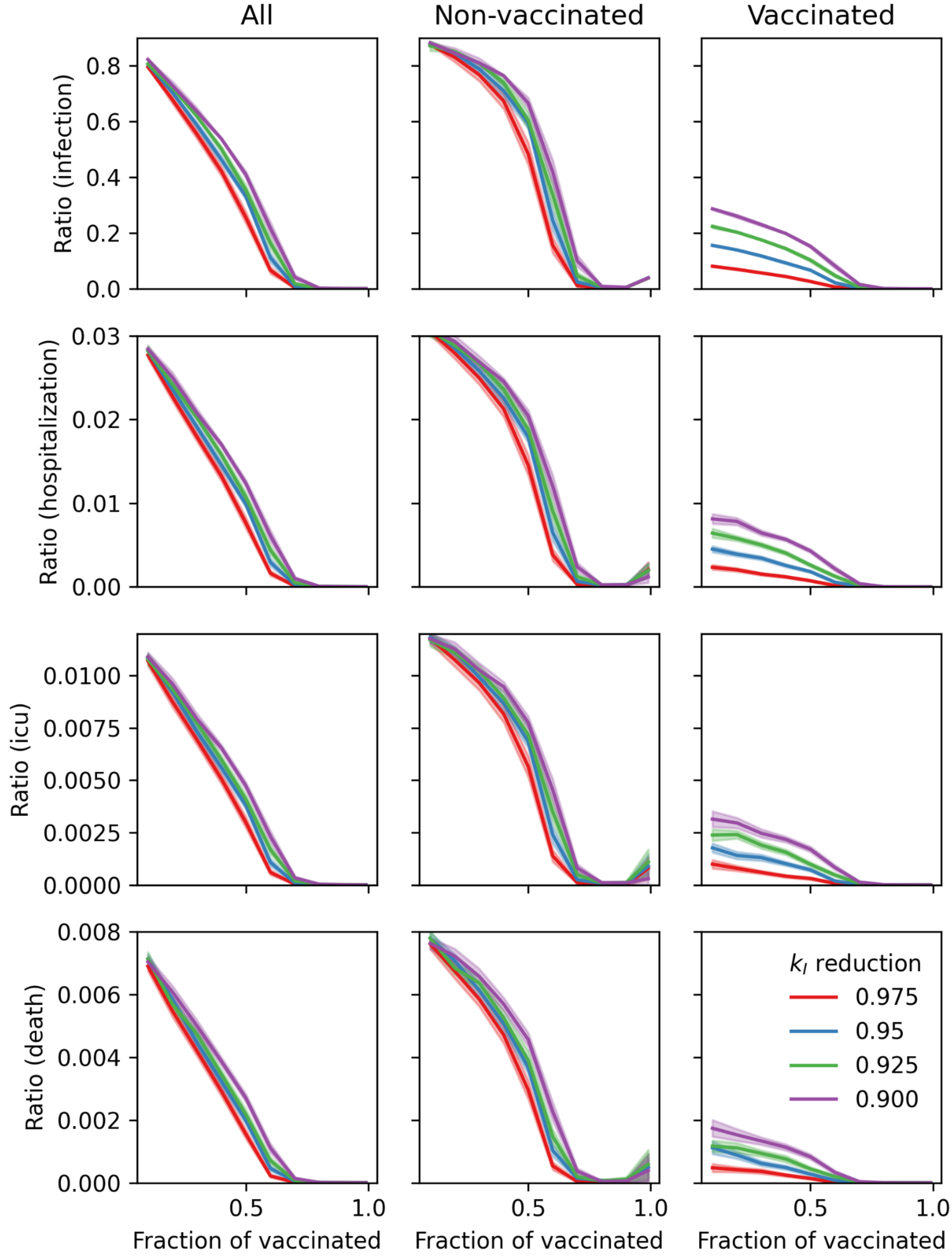

Figure S58: Impact of vaccine efficacies on agent health states (Gangelt). Ratios of agents in specific states to population (sub-groups) for different fractions of vaccinated individuals.

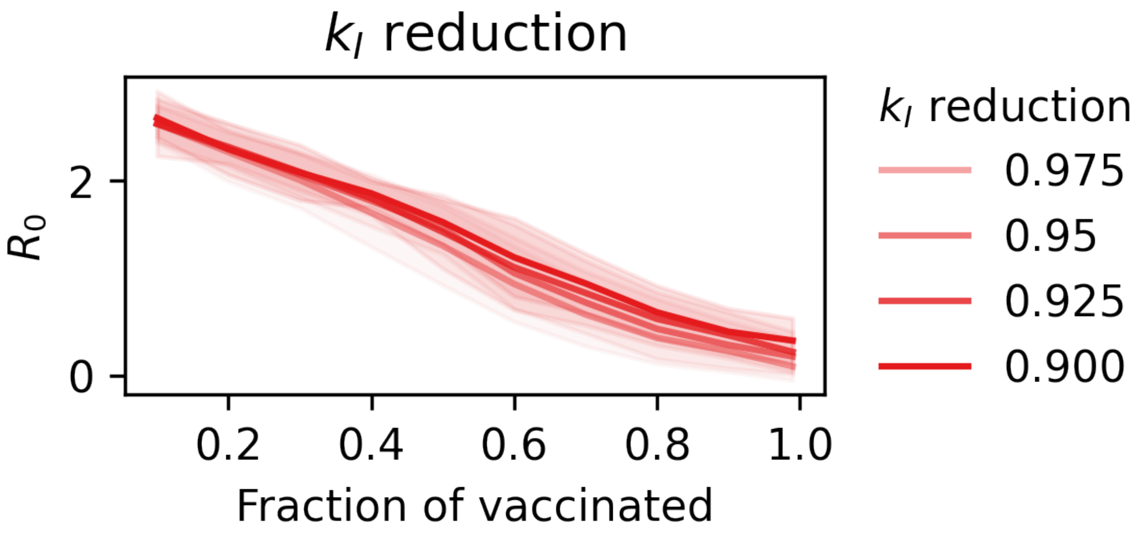

Figure S59: R$_{eff}$(0) for different vaccine efficacies (Gangelt). R$_{eff}$(0) values for fractions of vaccinated individuals did not differ in the tested range for vaccine efficacy.

4.9 Comparison to Homogeneous Mixing Approach

Classically, SIR modeling uses a homogeneous mixing (HM) approach, i.e. the modeled population is not distinguished by age, locations, or any other attribute that would entitle them to show distinguishable behavior. Our geospatial, demography-based agent-based approach reflects heterogeneity in the behavior of individuals, which is essentially different from the homogeneous mixing approach. This is also reflected in the outcome of various scenarios. The baseline scenario in Gerda shows greater variety in dynamics than the HM (**Fig. S60**).

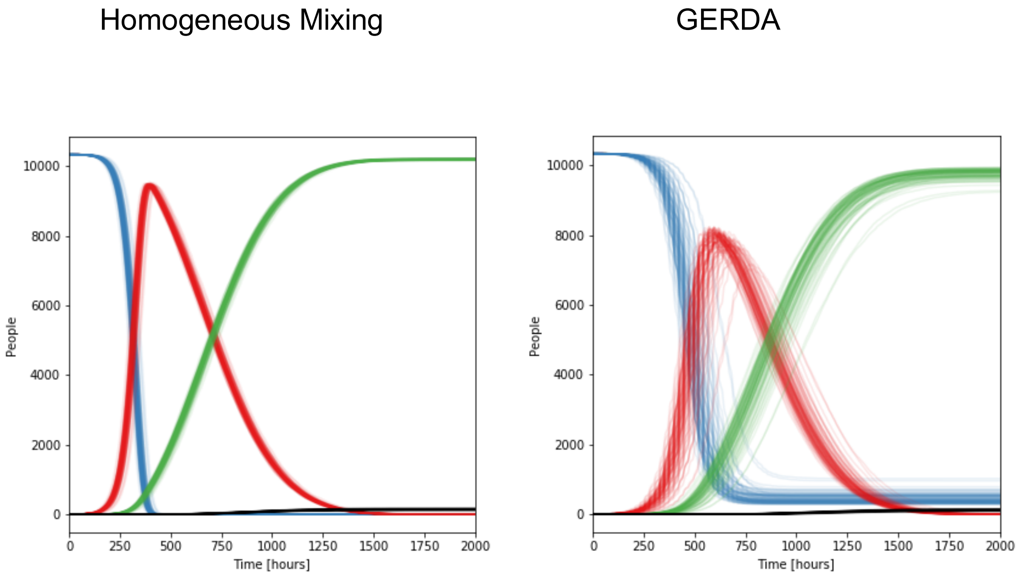

Figure S60: Health states homogeneous mixing baseline scenario (left) and in the original GERDA baseline scenario (right) for Gangelt. Resulting time courses for agent health states S (blue), I (red), R (green), and D (black) from 96 simulations for both scenarios for Gangelt.

Interactions between individuals as recorded in the HHIN as well as infections (iHHIN) also differ significantly between the two approaches. We show total and unique numbers of interactions as well as daily interactions between age groups and daily infections as heat maps (**Fig. S61**).

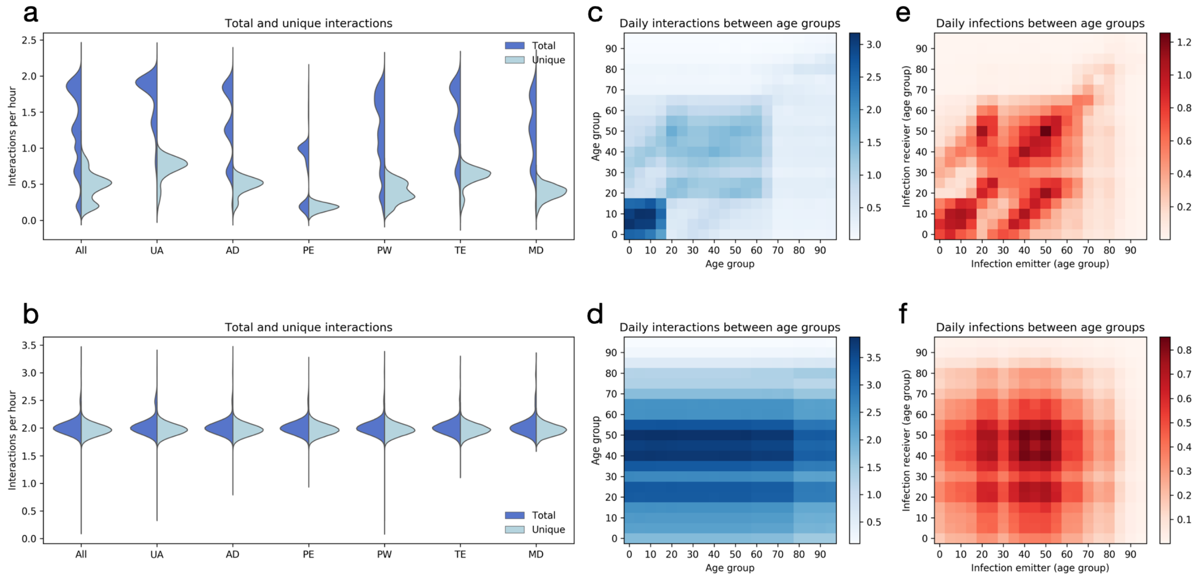

Figure S61: Comparison of interaction- and infection patterns between homogeneous heterogeneous simulations. (a) Distributions of average total and unique interactions among individuals of different occupations, in the heterogeneous model. **(b)** See description of (a), results obtained for the homogeneous mixing model where the age-distribution is still contained. **(c)** Mean daily unique interactions between different age-cohorts for the heterogeneous model. **(d)** See description of (c), results obtained for the homogeneous model. **(e)** Mean daily infection-transmissions between different age-cohorts for the heterogeneous model. **(f)** See description of (e), results obtained for the homogeneous model.

Also, the over- or underrepresentation of occupation groups looks different between homogeneous and heterogeneous approaches (**Fig. S44**). Homogeneous approaches don't show over- or underrepresentation (number not exactly zero result from stochastic noise).

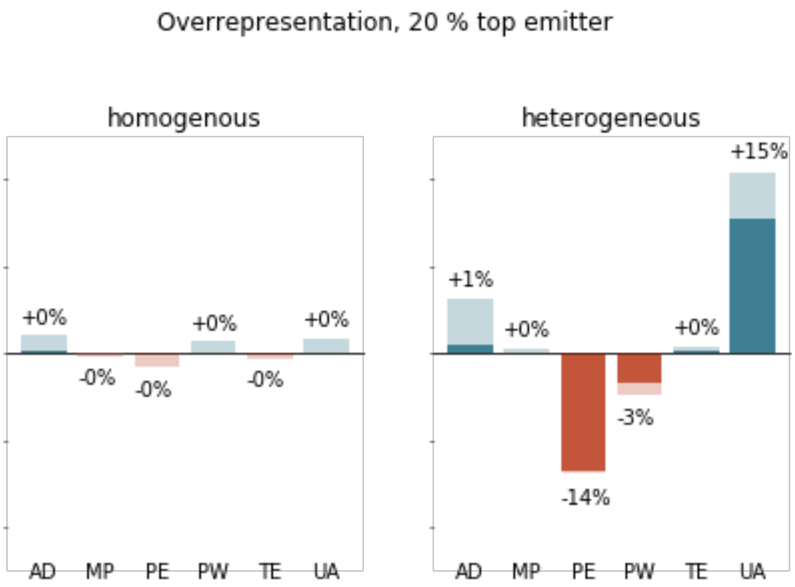

Figure S62: Comparison between homogenous and heterogenous mixing with respect to the over and under representation of agents with specific schedule types in the fractions of top spreaders. Difference in the fraction of spreaders with specific schedule type to the fraction of this type in the population.

4.10 Waning immunity in historical simulation

Waning immunity was implemented according to results obtained from Nordström et al. [52] and included in the long scale simulation presented in the main text (**Fig 5**). A vaccine efficacy of 97.5% against infection was considered, which posed a remaining infection risk of 2.5% for vaccinated individuals. 90 days after immunization the waning of immunity started at a constant daily rate, incrementing the baseline risk of infection by 0.32% per day. Although the registered cases in simulations that consider waning immunity tend to be slightly higher, especially for later time points, this difference is very small, particularly compared to the variability between simulations.

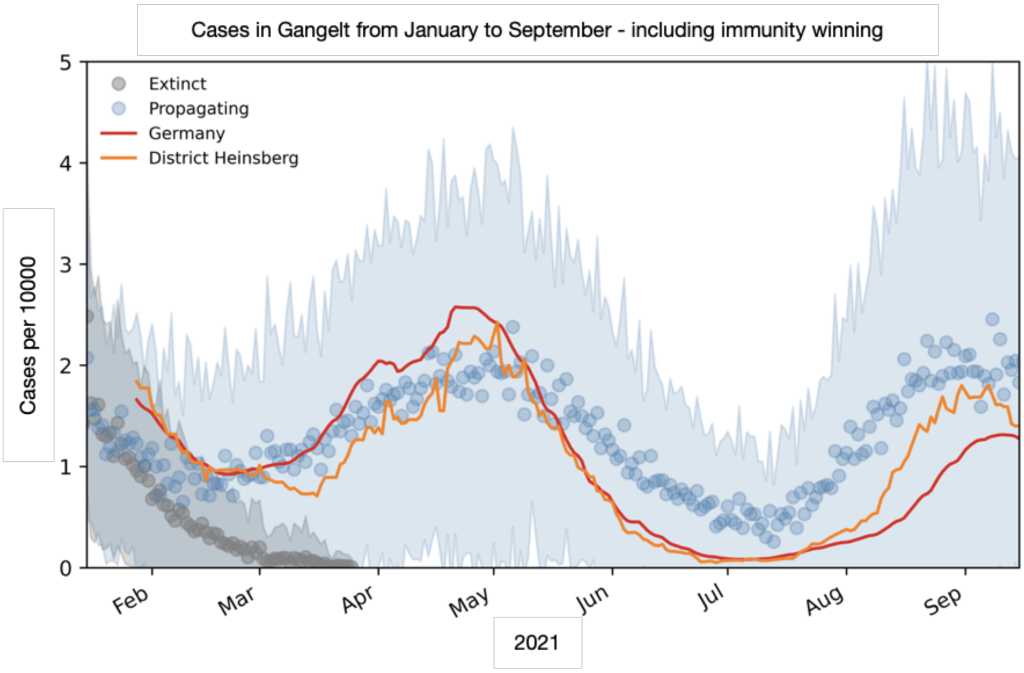

Figure S63: Evolution of cases when waning immunity is considered. The simulated registered cases are shown as light blue dots for simulations in which the virus did not vanish. The simulations with virus extinction are indicated by the grey dots. Both solid lines show real live data for Germany (red) or the district Heinsberg (orange) obtained from[53].

4.11 Heterogeneity of interaction within locations

Airborne transmission of viruses can be modeled via a random graph of the human-human interactions within a location as we defined in section 2.2.4. To test our results against this assumption, we analyzed the influence of heterogeneous mixing within a location by allowing for agent-specific distributions of the interactivities x_i for the i-th agent instead of using identical x_i for all agents. x_i was drawn from a distribution before the start of the simulation and kept fixed during the simulation. For comparison between different model versions, we adapted Eq.(1). We scaled μ with the average of the squared interactivities $< x^2 >$ to conserve the average interaction frequency .

$$P_{ab}^{int} = \frac{Minimum\left(n_{loc}-1, x_a x_b \frac{\mu}{<x^2>}\right)}{n_{loc}-1} \quad (12)$$

We tested x_i-values that are lognormally distributed or distributed according to a power law The obtained distributions are shown in **Figure S64**. To reflect in addition a potentially higher interactivity of children, we also tested the case of two different distributions for x_i depending on the age of the individuals with slightly different mean values. Results are shown below. Note that, we kept the scaling factor $n_{loc} - 1$ to ensure that the hourly probability to not have any significant contact does not vanish in locations with high agent numbers.

The effect on degree distributions is shown in **Figure S65**. The impact on interaction and infection patterns is represented in **Figure S66**.

We find that the heterogeneity in individual interactivities does not change the qualitative behavior with respect to age-specific infection and interaction patterns and the occurrence of bimodality after lifting of lockdown measures as shown in **Figure S67**. Heterogeneous mixing within locations, however, reduces the attack rates and broadens the distribution of total interactions or node degrees sorted according to archetype. **Figure S68** provides the unique and total interactions recorded for the different input distributions. **Figure S69** shows the mean degree for the hourly network per location type.

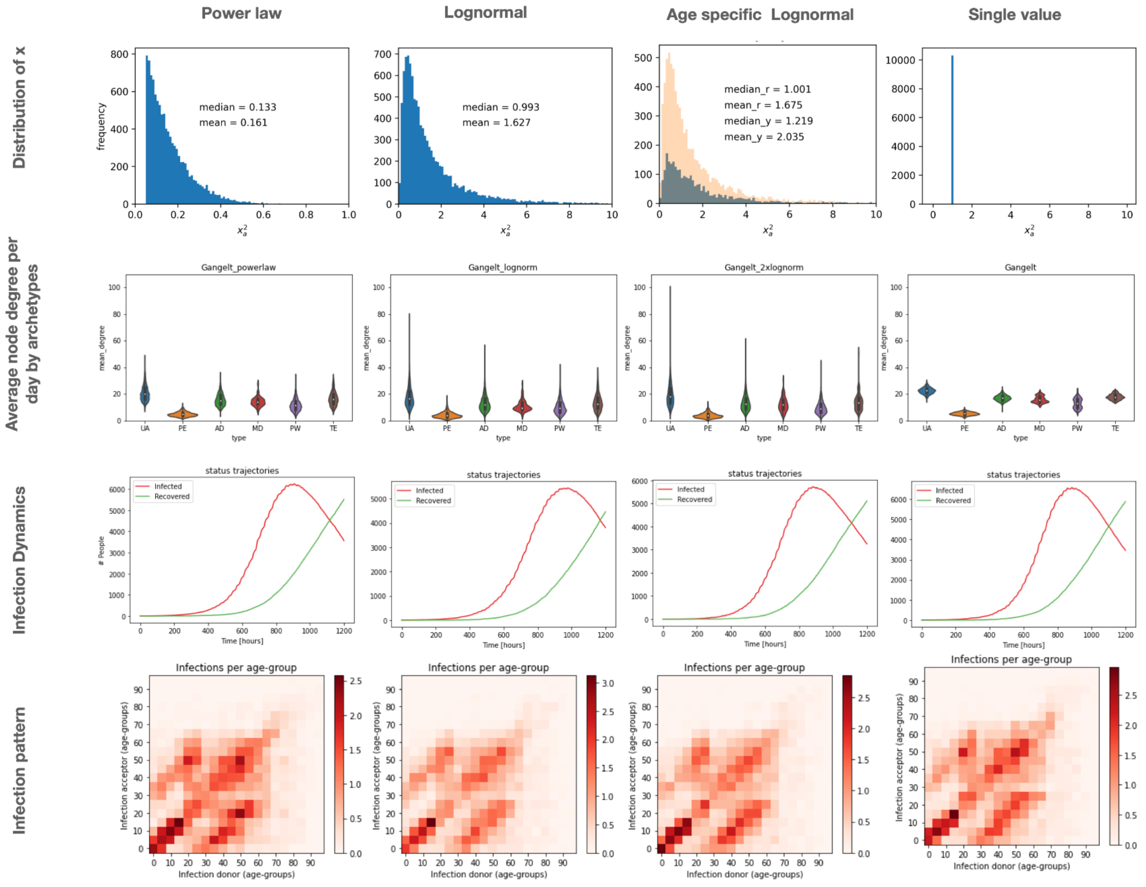

Figure S64. Impact of the choice for the agent-specific interaction modifier x_i. Columns: different input distributions, rows: simulation outputs. The first row shows the input distributions for the x_i, i.e. a power law distribution (slightly shifted to prevent an interactivity of exactly zero), a lognormal distribution, two different age-specific lognormal distributions, and finally the original choice of x_i=1. For the age-specific interactions, x_i was drawn for young individuals (y) between 3 years and 25 years of age from a lognormal distribution with mean of 0.2 and a STD of 1 and for the remaining individuals from a lognormal distribution with a mean of 0 and a STD of 1, respectively. The second row displays the resulting average node degrees within locations by archetype. The third row shows exemplary infection dynamics (single run, I red, R green). The bottom row presents the resulting age-specific infection patterns.

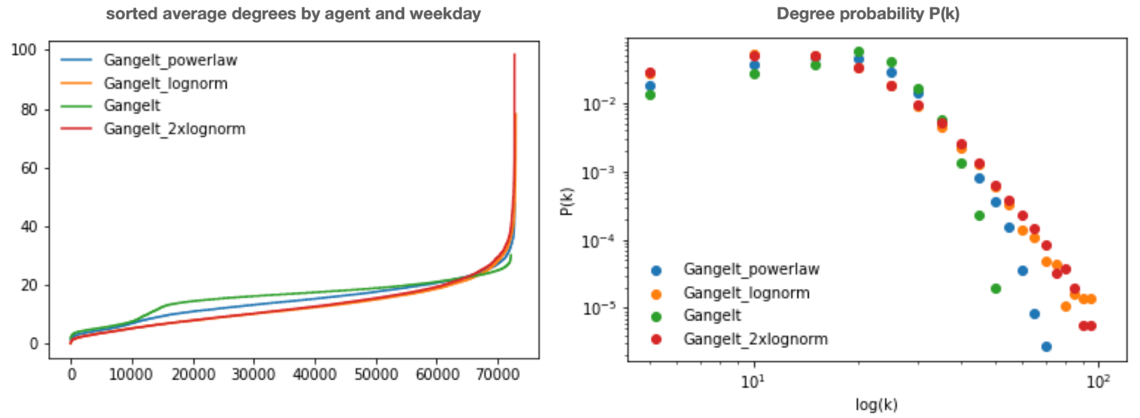

Figure S65. Degree distribution. Sorted degrees (individuals are sorted according to the degree they realize) and probabilities of degrees are presented for the input distributions used in **Figure S64**. For the left figure, the degrees for all days of a week and all agents are recorded and analyzed, resulting in 7x10.296 values on the x-axis.

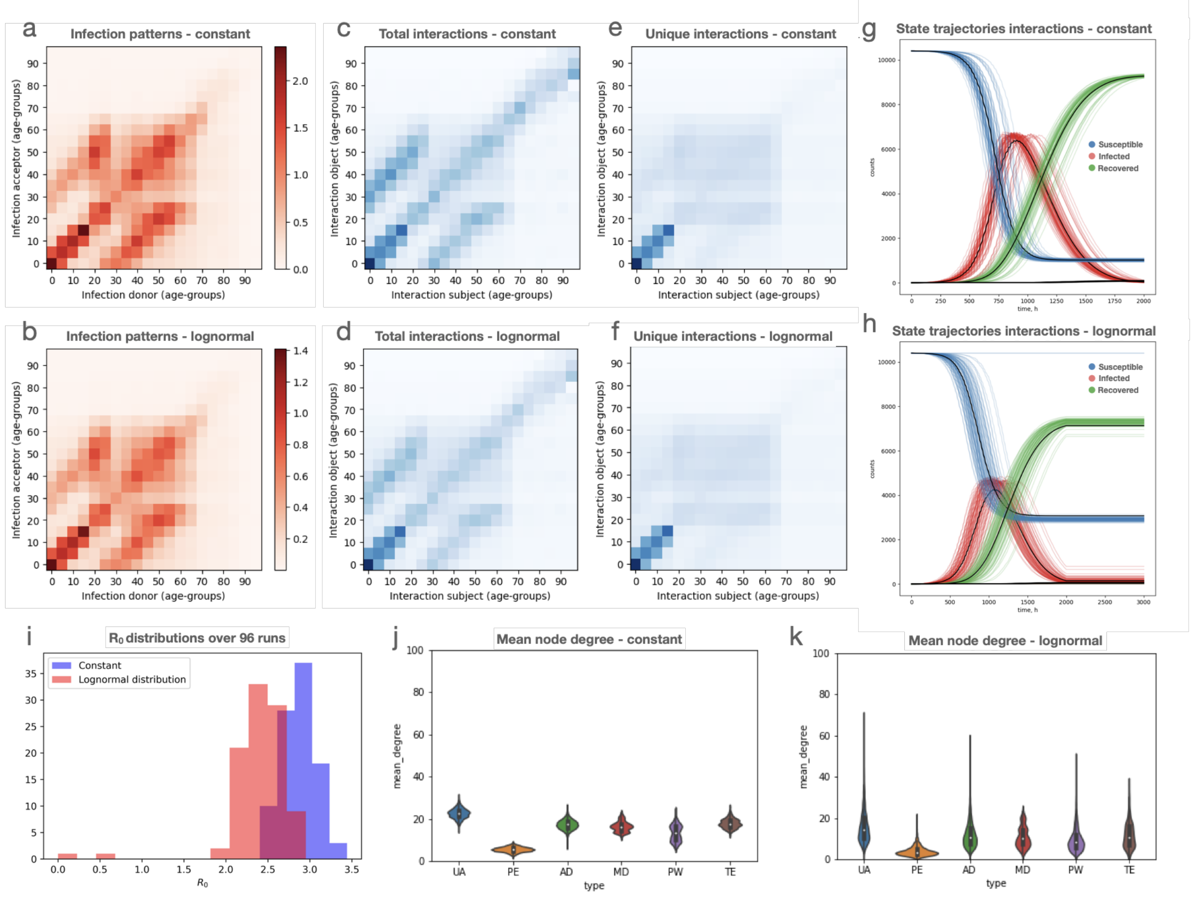

Figure S66. Comparison of repeated (N=96) simulations with either constant or lognormally distributed agent-specific interaction modifiers x_i. **a,b** Age-dependent infection patterns, **c,d** Age-dependent total interaction patterns, **e,f** Age-dependent unique contact patterns. g,h Infection dynamics for susceptibles (S) blue, infected (I) and recovered (R). **i** Distribution of R_0 for constant and lognormally distributed x_i. **j,k** Average node degrees by archetype for networks within locations.

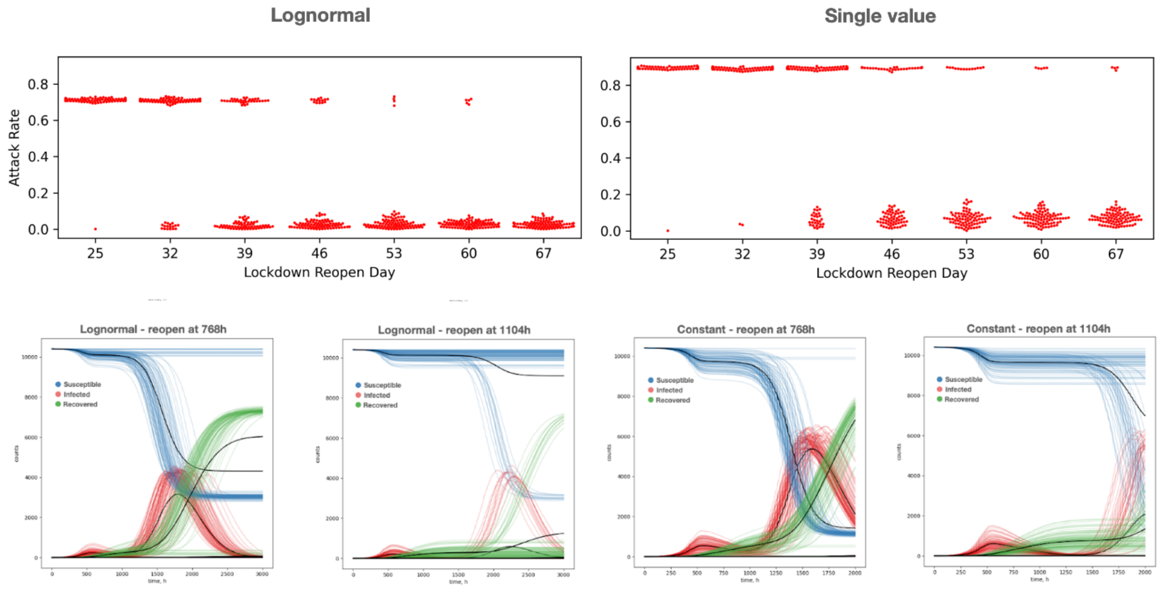

Figure S67. Bimodality after lifting of lockdown measures remains if heterogeneous mixing within locations is considered. Upper row: Attack rates for simulation of the baseline scenario with a lockdown at 18 days after first infection and a reopening after different days. AR is given for different reopening times (25-67 days). The lower row shows exemplary time courses for susceptible and infected individuals for N=96 repetitions for reopening days 32 and 46. AR is reduced when heterogeneous mixing is considered. (Left: lognormal input distribution, right: x_i =1)

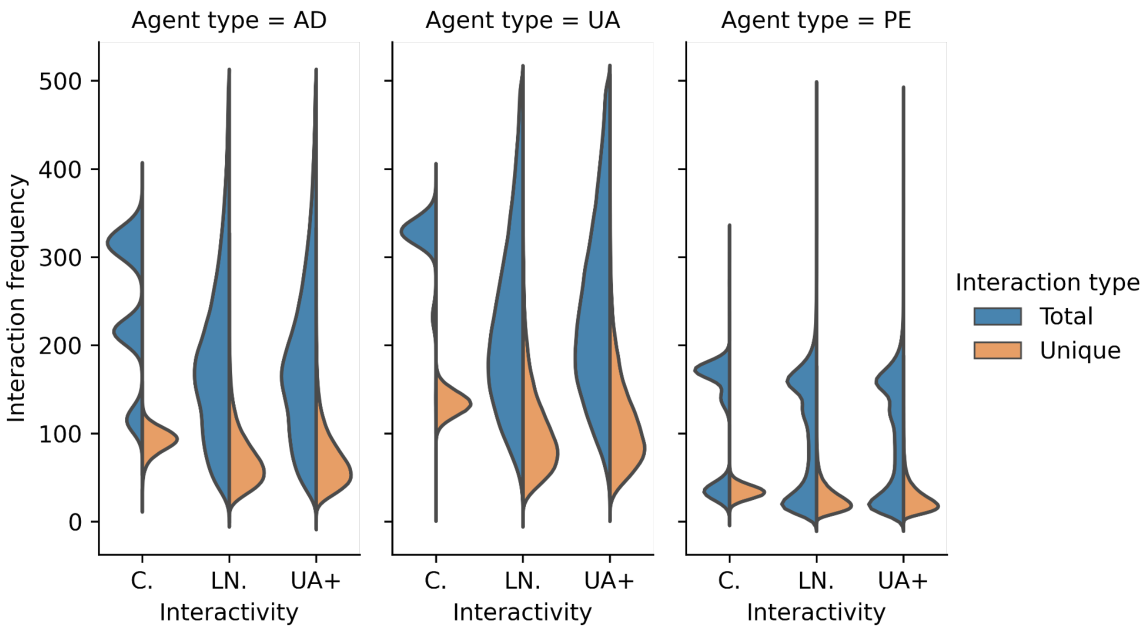

Figure S68. Distribution of total and unique weekly interactions. The distributions of weekly interactions (total and unique) for different agent types are displayed for different interactivity distribution methods (C. : constant single value for all agents, LN.: lognormally distributed interactivities, UA+ : lognormally distributed with higher values for underaged agents).

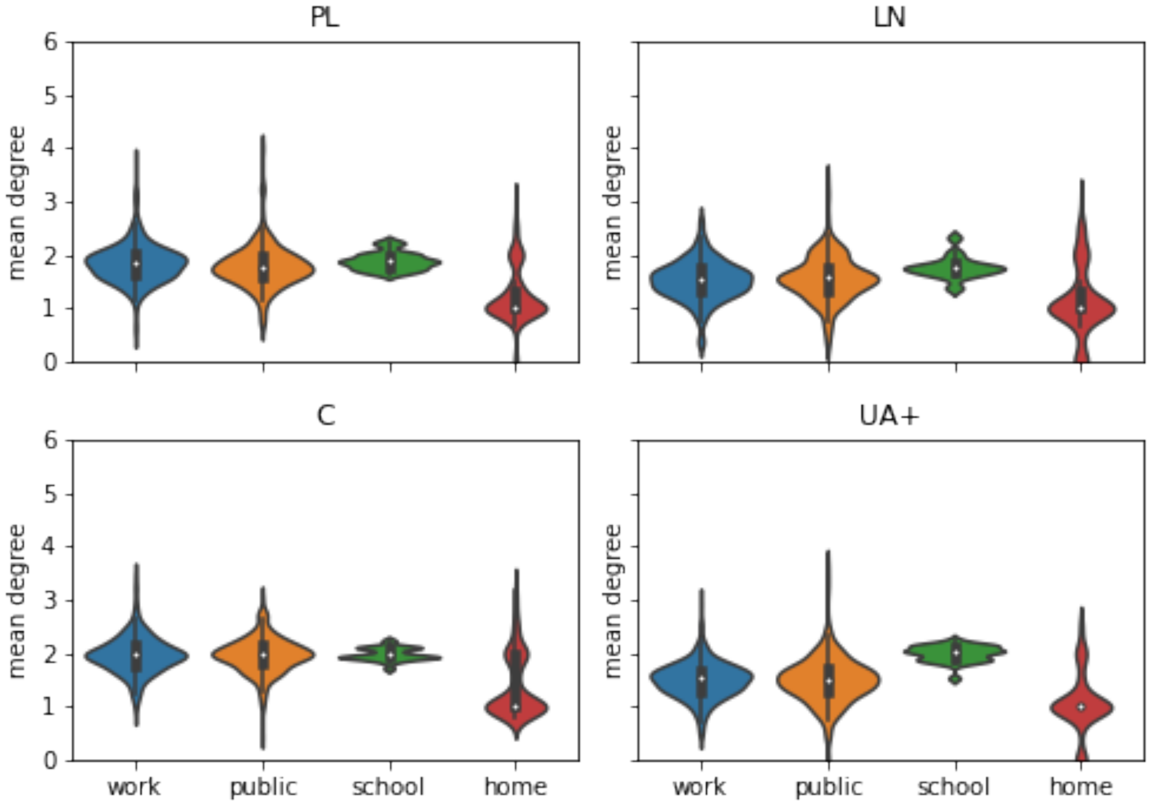

Figure S69. Mean degree for the hourly network per location type. The mean degree is shown for different interactivity distribution methods (C. : constant single value for all agents, LN.: lognormally distributed interactivities, UA+ : lognormally distributed with higher values for underaged agents)

4.12 The movie

Movie S1 [**https://www.tbp-klipp.science/GERDA-model/Movie-1.mp4**] visualizes the temporal and spatial dynamics of infection spreading resulting from the simulation of the baseline scenario with a still image in **Fig. 1b**. Note that the daily rhythm shown in the movie results from individuals moving between their respective homes and workplaces. The infection starts with two infected individuals in one household at time 0 h. At times 100 h and 200 h more and more infected agents are observed, especially at the geospatial hubs (e.g., center of the town).

5 References

1. Ahn, D.-G. *et al.* Current Status of Epidemiology, Diagnosis, Therapeutics, and Vaccines for Novel Coronavirus Disease 2019 (COVID-19). *J. Microbiol. Biotechnol.* **30**, 313–324 (2020).
2. Esakandari, H. *et al.* A comprehensive review of COVID-19 characteristics. *Biol. Proced. Online* **22**, 19 (2020).
3. Pascarella, G. *et al.* COVID-19 diagnosis and management: a comprehensive review. *J. Intern. Med.* **288**, 192–206 (2020).
4. Zhou, M.-Y. *et al.* From SARS to COVID-19: What we have learned about children infected with COVID-19. *Int. J. Infect. Dis.* (2020).
5. Vellas, C., Delobel, P., de Souto Barreto, P. & Izopet, J. COVID-19, Virology and Geroscience: A Perspective. *J. Nutr. Health Aging* **24**, 685–691 (2020).
6. Britton, T., Ball, F. & Trapman, P. A mathematical model reveals the influence of population heterogeneity on herd immunity to SARS-CoV-2. *Science* eabc6810 (2020) doi:10.1126/science.abc6810.
7. Zhang, J. *et al.* Changes in contact patterns shape the dynamics of the COVID-19 outbreak in China. *Science* **368**, 1481–1486 (2020).
8. Karin, O. *et al.* Cyclic exit strategies to suppress COVID-19 and allow economic activity. (2020) doi:10.1101/2020.04.04.20053579.
9. Jonkers, A. R. T., Sharkey, K. J. & Christley, R. M. Preventable H5N1 avian influenza epidemics in the British poultry industry network exhibit characteristic scales. *J. R. Soc. Interface* **7**, 695–701 (2010).
10. Li, H. *et al.* Updated Approaches against SARS-CoV-2. *Antimicrob. Agents Chemother.* **64**, (2020).
11. Salzberger, B. *et al.* Epidemiologie von SARS-CoV-2-Infektion und COVID-19. *Internist* **61**, 782–788 (2020).
12. Deeks, J. J. *et al.* Antibody tests for identification of current and past infection with SARS-CoV-2. *Cochrane Database Syst. Rev.* **6**, CD013652 (2020).
13. Van den Broeck, W. *et al.* The GLEaMviz computational tool, a publicly available software to

explore realistic epidemic spreading scenarios at the global scale. *BMC Infect. Dis.* **11**, 37 (2011).

14. Bouffanais, R. & Lim, S. S. Cities — try to predict superspreading hotspots for COVID-19. *Nature* **583**, 352–355 (2020).
15. Kermack, W. O., McKendrick, A. G. & Walker, G. T. A contribution to the mathematical theory of epidemics. *Proceedings of the Royal Society of London. Series A, Containing Papers of a Mathematical and Physical Character* **115**, 700–721 (1927).
16. Maier, B. F. & Brockmann, D. Effective containment explains subexponential growth in recent confirmed COVID-19 cases in China. *Science* **368**, 742–746 (2020).
17. Stonedahl, F. & Wilensky, U. NetLogo Virus on a Network model. *Center for Connected Learning and Computer-Based Modeling, Northwestern University, Evanston, IL* (2008).
18. Ferguson, N. M. *et al.* Strategies for containing an emerging influenza pandemic in Southeast Asia. *Nature* **437**, 209–214 (2005).
19. Chao, D. L., Halloran, M. E., Obenchain, V. J. & Longini, I. M., Jr. FluTE, a publicly available stochastic influenza epidemic simulation model. *PLoS Comput. Biol.* **6**, e1000656 (2010).
20. Gomez, J., Prieto, J., Leon, E. & Rodriguez, A. INFEKTA: A General Agent-based Model for Transmission of Infectious Diseases: Studying the COVID-19 Propagation in Bogotá-Colombia. *medRxiv* (2020).
21. Lai, S. *et al.* Effect of non-pharmaceutical interventions to contain COVID-19 in China. *Nature* **585**, 410–413 (2020).
22. Quilty, B. J. *et al.* Quarantine and testing strategies in contact tracing for SARS-CoV-2: a modelling study. *Lancet Public Health* **6**, e175–e183 (2021).
23. Kissler, S. M., Tedijanto, C., Goldstein, E., Grad, Y. H. & Lipsitch, M. Projecting the transmission dynamics of SARS-CoV-2 through the postpandemic period. *Science* **368**, 860–868 (2020).
24. Ferretti, L. *et al.* Quantifying SARS-CoV-2 transmission suggests epidemic control with digital contact tracing. *Science* **368**, (2020).
25. Pullano, G. *et al.* Underdetection of cases of COVID-19 in France threatens epidemic control. *Nature* **590**, 134–139 (2021).
26. Di Domenico, L., Pullano, G., Sabbatini, C. E., Boëlle, P.-Y. & Colizza, V. Modelling safe protocols for reopening schools during the COVID-19 pandemic in France. *Nat. Commun.* **12**, 1073 (2021).

27. Mistry, D. *et al.* Inferring high-resolution human mixing patterns for disease modeling. *Nat. Commun.* **12**, 323 (2021).
28. Sadanand, S. Vaccination: the present and the future. *Yale J. Biol. Med.* **84**, 353–359 (2011).
29. Dignum, F. *et al.* Analysing the Combined Health, Social and Economic Impacts of the Corovanvirus Pandemic Using Agent-Based Social Simulation. *Minds Mach.* **30**, 177–194 (2020).
30. Karpov, Y. G. Simulation modeling systems. Introduction to modeling with AnyLogic 5. *St. Petersburg Math. J.* (2005).
31. Wilensky, U. NetLogo, Center for Connected Learning and Computer-Based Modeling, Northwestern University. *Evanston, IL* (1999).
32. Collier, N., Howe, T. & North, M. Onward and upward: The transition to Repast 2.0. (01 2003).
33. Horni, A., Nagel, K. & Axhausen, K. W. *The Multi-Agent Transport Simulation MATSim*. (Ubiquity Press, 2016).
34. Müller, S. A. *et al.* Using mobile phone data for epidemiological simulations of lockdowns: government interventions, behavioral changes, and resulting changes of reinfections. *Research Square* (2020) doi:10.21203/rs.3.rs-47857/v1.
35. Chang, S. L., Harding, N., Zachreson, C., Cliff, O. M. & Prokopenko, M. Modelling transmission and control of the COVID-19 pandemic in Australia. *Nat. Commun.* **11**, 5710 (2020).
36. Kerr, C. C. *et al.* Covasim: An agent-based model of COVID-19 dynamics and interventions. *PLoS Comput. Biol.* **17**, e1009149 (2021).
37. Mossong, J. *et al.* Social contacts and mixing patterns relevant to the spread of infectious diseases. *PLoS Med.* **5**, e74 (2008).
38. Boeing, G. OSMnx: New methods for acquiring, constructing, analyzing, and visualizing complex street networks. *Comput. Environ. Urban Syst.* **65**, 126–139 (2017).
39. Coronavirus Disease 2019 (COVID-19) Daily Situation Report of the Robert Koch Institute. *2020-04-13-en.pdf*. (2020).
40. Karagiannidis, C. *et al.* Case characteristics, resource use, and outcomes of 10 021 patients with COVID-19 admitted to 920 German hospitals: an observational study. *The Lancet Respiratory Medicine* **8**, 853–862 (2020).
41. Streeck, H. *et al.* Infection fatality rate of SARS-CoV-2 infection in a German community with a super-spreading event. *Infectious Diseases (except HIV/AIDS)* (2020)

doi:10.1101/2020.05.04.20090076.

42. an der Heiden, M. & Buchholz, U. Modellierung von Beispielszenarien der SARS-CoV-2-Epidemie 2020 in Deutschland. (2020) doi:10.25646/6571.2.
43. He, X. *et al.* Temporal dynamics in viral shedding and transmissibility of COVID-19. *Nat. Med.* **26**, 672–675 (2020).
44. Ferretti, L. *et al.* The timing of COVID-19 transmission. *bioRxiv* (2020) doi:10.1101/2020.09.04.20188516.
45. Hart, W. S. *et al.* Inference of SARS-CoV-2 generation times using UK household data. *bioRxiv* (2021) doi:10.1101/2021.05.27.21257936.
46. Ganyani, T. *et al.* Estimating the generation interval for coronavirus disease (COVID-19) based on symptom onset data, March 2020. *Euro Surveill.* **25**, (2020).
47. Du, Z. *et al.* Serial interval of COVID-19 among publicly reported confirmed cases. *Emerg. Infect. Dis.* **26**, 1341–1343 (2020).
48. MacQueen, J. & Others. Some methods for classification and analysis of multivariate observations. in *Proceedings of the fifth Berkeley symposium on mathematical statistics and probability* vol. 1 281–297 (Oakland, CA, USA, 1967).
49. Lloyd, S. P. Least squares quantization in pcm. technical note. *Bell Laboratories* (1957).
50. Tavenard, R. *et al.* Tslearn, A Machine Learning Toolkit for Time Series Data. *J. Mach. Learn. Res.* **21**, 1–6 (2020).
51. Rousseeuw, P. J. Silhouettes: A graphical aid to the interpretation and validation of cluster analysis. *J. Comput. Appl. Math.* **20**, 53–65 (1987).
52. Nordström, P., Ballin, M. & Nordström, A. Effectiveness of Covid-19 Vaccination Against Risk of Symptomatic Infection, Hospitalization, and Death Up to 9 Months: A Swedish Total-Population Cohort Study. (2021) doi:10.2139/ssrn.3949410.
53. COVID-19 Datenhub. https://npgeo-corona-npgeo-de.hub.arcgis.com.
